# Quantization of the energy for the inhomogeneous Allen–Cahn mean curvature

**DOI:** 10.1007/s00208-024-02909-6

**Published:** 2024-06-14

**Authors:** Huy The Nguyen, Shengwen Wang

**Affiliations:** School of Mathematical Sciences, Queen Mary University of London, Mile End Road, London, E1 4NS UK

## Abstract

We consider the varifold associated to the Allen–Cahn phase transition problem in ${\mathbb {R}}^{n+1}$(or $n+1$-dimensional Riemannian manifolds with bounded curvature) with integral $L^{q_0}$ bounds on the Allen–Cahn mean curvature (first variation of the Allen–Cahn energy) in this paper. It is shown here that there is an equidistribution of energy between the Dirichlet and Potential energy in the phase field limit and that the associated varifold to the total energy converges to an integer rectifiable varifold with mean curvature in $L^{q_0}, q_0 > n$. The latter is a diffused version of Allard’s convergence theorem for integer rectifiable varifolds.

## Introduction

Let $\Omega \subset \mathbb (M^{n+1},g)$ be an open subset in a Riemannian manifold with bounded curvature. Consider $u\in W^{2,p}(\Omega )$ satisfying the following equation

1.1 $$\begin{aligned} \varepsilon \Delta u_\varepsilon -\frac{W'(u_\varepsilon )}{\varepsilon }=f_\varepsilon , \end{aligned}$$

where $W(t)=\frac{(1-t^2)^2}{2}$ is a double-well potential. The Eq. (1.1) can be viewed as a prescribed first variation problem to the Allen–Cahn energy

$$\begin{aligned} E_\varepsilon (u_\varepsilon )=\int _\Omega \left( \frac{\varepsilon |\nabla u_\varepsilon |^2}{2}+\frac{W(u_\varepsilon )}{\varepsilon }\right) dx. \end{aligned}$$

For any compactly supported test vector field $\eta \in C_c^\infty (\Omega ,{\mathbb {R}}^{n+1})$, we have a variation $u_s(x)=u\left( x+s\eta (x)\right)$ and the first variation formula at $u_0=u_\varepsilon$ is given by

1.2 $$\begin{aligned} \begin{aligned} \frac{d}{ds}\bigg |_{s=0}E_\varepsilon \left( u_s\right)&=\int _\Omega \left( -\varepsilon \Delta u_\varepsilon +\frac{W'(u_\varepsilon )}{\varepsilon }\right) \langle \nabla u_\varepsilon , \eta \rangle dx\\&=-\int _\Omega \left( \frac{f_\varepsilon }{\varepsilon |\nabla u_\varepsilon |}\right) \langle \nu ,\eta \rangle \varepsilon |\nabla u_\varepsilon |^2dx, \end{aligned}\nonumber \\ \end{aligned}$$

where $\nu =\frac{\nabla u_\varepsilon }{|\nabla u_\varepsilon |}$ is a unit normal to the level sets at non-critical points of *u*.

By [5, 6, 9] using the framework of [3], the sequence of functionals $E_\varepsilon$ $\Gamma$-converges to the *n*-dimensional area functional as $\varepsilon \rightarrow 0$. This shows that minimizing solutions to (1.1) with $f_\varepsilon =0$ converge as $\varepsilon \rightarrow 0$ to area minimizing hypersurfaces. For general critical points ($f_\varepsilon =0$) a deep theorem of Hutchinson–Tonegawa [4, Theorem 1] shows the diffuse varifold obtained by smearing out the level sets of *u* converges to limit which is a stationary varifold with *a*.*e*. integer density. The main result of this paper is to prove Hutchinson–Tonegawa’s Theorem [4, Theorem 1] in the context of natural integrability conditions on the first variation of $E_\varepsilon$. Under suitable controls on the first variation of the energy functional $E_\varepsilon$ (the diffuse mean curvature) we can show comparable behaviour for the limit. In the case where $n=2,3$ Röger–Schätzle [8] have shown under the assumption

$$\begin{aligned} \liminf _{\varepsilon \rightarrow 0^+} \left( E_\varepsilon (u_\varepsilon )+ \frac{1}{\varepsilon } \Vert f_\varepsilon \Vert ^2_{L^2(\Omega )} \right) < \infty \end{aligned}$$

that the limit is an integer rectifiable varifold with $L^2$ generalised mean curvature.

The main focus of this paper is to generalise this result to higher dimensions. Before we state our main theorem, we give a choice of the diffused analogue of “mean curvature" in the Allen–Cahn setting, which will be used to state our bounded $L^{q_0}$ Allen–Cahn mean curvature condition in the theorem.

Recall that for an embedded hypersurface $\Sigma ^n\subset \Omega \subset {\mathbb {R}}^{n+1}$ restricted to a bounded domain $\Omega$ and a compactly supported variation $\Sigma _s$ with $\Sigma _0=\Sigma$, we have the first variation area at $s=0$ given by

1.3 $$\begin{aligned} \frac{d}{ds}\bigg |_{s=0}{\text {Area}}(\Sigma _s\cap \Omega )=-\int _{{\mathbb {R}}^{n+1}} \langle {\textbf{H}},\eta \rangle d\mu _\Sigma =\int _{{\mathbb {R}}^{n+1}} H\langle \nu ,\eta \rangle d\mu _\Sigma , \end{aligned}$$

where *H* is the mean curvature scalar, ${\textbf{H}}=-H\nu$ is the mean curvature vector, $\nu$ is a unit normal vector field, $\eta$ is the variation vector field, and $d\mu _\Sigma$ is the hypersurface measure. By comparing the first variation formula (1.2) for Allen–Cahn energy and the first variation formula (1.3) for area , we can see that $\left( \frac{f_\varepsilon }{\varepsilon |\nabla u|}\right)$ roughly plays the role of the mean curvature scalar in the Allen–Cahn setting. In [1], a result of Allard implies that if a sequence of integral varifolds has $L^{q_0}$ integrable mean curvature scalar with $q_0>n$, then after passing to a subsequence, there is a limit varifold which is also integer rectifiable.

Under similar conditions on $L^{q_0}$ integrability of the term $\left( \frac{f_\varepsilon }{\varepsilon |\nabla u|}\right)$ with $q_0>n$, we prove the integer rectifiability of the limit of sequences of Allen–Cahn varifolds :

### Theorem 1.1

Let $u_{\varepsilon }\in W^{1,2}(\Omega ), \Omega \subset {\mathbb {R}}^{n+1}$ satisfy Eq. (1.1) with $\varepsilon \rightarrow 0$ and $f_\varepsilon \in L^1(\Omega )$. If any one of the following holds:

1. Bounds on the total energy
  1.4 $$\begin{aligned} \int _\Omega \left( \frac{\varepsilon |\nabla u_\varepsilon |^2}{2}+\frac{W(u_\varepsilon )}{\varepsilon }\right) dx\le E_0; \end{aligned}$$
2. Uniform $$L^\infty $$ bounds
  1.5 $$\begin{aligned} \Vert u_\varepsilon \Vert _{L^\infty (\Omega )}\le c_0; \end{aligned}$$
3. $L^{q_0}$ bounds on the diffuse mean curvature
  1.6 $$\begin{aligned} \int _\Omega \left( \frac{|f_\varepsilon |}{\varepsilon |\nabla u_\varepsilon |}\right) ^{q_0}\varepsilon |\nabla u_\varepsilon |^2dx \le \Lambda _0 \end{aligned}$$
  for some $$q_0> n$$;

then after passing to a subsequence, we have for the associated varifolds (see Sect. 2 for the definition) $V_{u_\varepsilon }\rightarrow V_\infty$ weakly and

1. $V_\infty$ is an integral *n*-rectifiable varifold;
2. For any $B_r(x_0)\subset \subset \Omega$, the $L^{q_0}$ norm of the generalized mean curvature of $V_\infty$ is bounded by $\Lambda _0$;
3. The discrepancy measure $\left( \frac{\varepsilon |\nabla u_\varepsilon |^2}{2}-\frac{W(u_\varepsilon )}{\varepsilon }\right) \rightarrow 0$ in $L^1_{loc}$ as $\varepsilon \rightarrow 0$ (c.f. Proposition 4.4).

This theorem shows we can prove a result analagous to Hutchinson–Tonegawa [4], Tonegawa [10] and show as $\varepsilon \rightarrow 0$, the diffuse varifold associated to the Allen–Cahn functional converges to an integer rectifiable varifold. This has some similarities with Allard’s compactness theorem for rectifiable varifolds and for integral varifolds but here the sequence consists of diffuse varifolds and hence we require stronger conditions on the proposed mean curvature. As we shall see in a later paper, these conditions are exactly what is required to prove a version of Allard’s regularity theorem for Allen–Cahn varifolds.

In the proof of Theorem 1.1, we also obtained a variational approximation of a class of integral mean curvature functional via $\Gamma$ - convergence by a sequence functionals from the phase-field model.

### Corollary 1.2

Let $u\in W^{1,2}(\Omega ), \Omega \subset {\mathbb {R}}^{n+1}$ satisfy Eq. (1.1) with $u_\varepsilon =u$ and ${\mathcal {F}}_\varepsilon :L^1(\Omega )\rightarrow {\mathbb {R}}$ be a sequence of functionals defined by

$$\begin{aligned} {\mathcal {F}}_\varepsilon (u)=\int _\Omega \left( \frac{\varepsilon |\nabla u|^2}{2}+\frac{W(u)}{\varepsilon }\right) dx+\int _\Omega \left( \frac{|\varepsilon \Delta u -\frac{W'(u)}{\varepsilon }|}{\varepsilon |\nabla u|}^{q_0}\right) \varepsilon |\nabla u|^2dx, \end{aligned}$$

for any $q_0>n$. Then for any $\chi =2\chi _E-1$ with $E\subset \Omega ,\partial E\cap \Omega \in C^2$ where $\chi _E$ is the characteristic function for *E*, there holds

$$\begin{aligned} \Gamma (L^1(\Omega )) - \lim _{\varepsilon \rightarrow 0}{\mathcal {F}}_\varepsilon (\chi )={\mathcal {F}}(\chi )=:\alpha {\mathcal {H}}^n(\partial E\cap \Omega )+\alpha \int _{\partial E\cap \Omega }|{\textbf{H}}_{\partial E}|^{q_0}d{\mathcal {H}}^n, \end{aligned}$$

where $\alpha =\int _{-\infty }^\infty (\tanh ' x)^2dx$ is the total energy for the 1-d heteroclinic solution Allen-Cahn equation, ${\mathcal {H}}^n$ is the *n*-dimensional Hausdorff measure, and ${\textbf{H}}_{\partial E}$ is the mean curvature of $\partial E$.

Our result can also imply some previous convergence results under various integrability conditions for the inhomogeneous term and its derivatives. (Notice that we do not require any integrability condition on the derivative of the inhomogeneous term *f* in Theorem 1.1).

### Corollary 1.3

If $u_\varepsilon$ satisfies (1.1) and of one of the following conditions holds:

1. $$\begin{aligned}&\Vert f_\varepsilon \Vert _{L^s(\Omega )}\le C_1\varepsilon ^\frac{1}{2},&\quad \text { for some }2<s<n\\&\left\| \frac{f_\varepsilon }{\varepsilon |\nabla u_\varepsilon |}\right\| _{L^t(\Omega )}\le C_2,&\quad \text { for some }t>\frac{n-2}{s-2}s>\max \{s,n-2\}; \end{aligned}$$
2. $$\begin{aligned} \left\| \frac{f_\varepsilon }{\varepsilon |\nabla u_\varepsilon |}\right\| _{W^{1,p}(\Omega )}\le C,\quad \text { for some }p>\frac{n+1}{2},\quad \text {(c.f. [11])}; \end{aligned}$$
3. $$\begin{aligned}&\Vert f_\varepsilon \Vert _{L^2(\Omega )}\le C_1\varepsilon ^\frac{1}{2},&\quad \text { if the ambient dimension }n+1=2,\quad \text {(c.f. [8])}\\&\left\| \frac{f_\varepsilon }{\varepsilon |\nabla u_\varepsilon |}\right\| _{L^\infty (\Omega )}\le C_2,&\quad \text { if the ambient dimension }n+1\ge 3; \end{aligned}$$

then after passing to a subsequence as $\varepsilon \rightarrow 0$, the associated varifolds $V_\varepsilon$ converge to an integral *n*-rectifiable varifold with generalized mean curvature in $L^{q_0}$ for some $q_0>n$.

Here we give an overview of our proof. In Sect. 2, we gather together some standard notation on varifolds and the first variation. In Sect. 3, we prove the main estimates required for the proof of the integrality and rectifiability. Specifically we will need a monotonicity formula. For the homogeneous Allen–Cahn equation and Allen–Cahn flow, a strict monotonicity formula can be proven due to Modica’s estimate showing the discrepancy is negative. This estimate is not true without a homogeneous left hand side to Eq. (1.1). Instead we will use the integral bound (1.6) to derive a decay bound for $L^1$ norm of the discrepancy which we eventually show vanishes in the limit $\varepsilon \rightarrow 0$. This estimate constitutes one of the main advances of this paper. In Sect. 4 we show the limiting varifold we obtain as $\varepsilon \rightarrow 0$ is a rectifiable set and in Sect. 5 we show the limiting varifold is in addition integral. In Sect. 6, we prove Corollary 1.3 and Corollary 1.2.

## Preliminaries and notations

Throughout the paper, we will denote a constant by *C* if it only depends on the constants $n,E_0,c_0,\Lambda _0$ which appear in the conditions of Theorem 1.1. At certain points we may increase this constant in some steps of the argument, but we will not relabel the constant unless there is a risk of confusion from the context. We associate to each solution of (1.1) a varifold in the following way : let $G(n+1,n)$ denote the Grassmannian (the space of unoriented *n*-dimensional subspaces in ${\mathbb {R}}^{n+1}$). We regard $S\in G(n+1,n)$ as the $(n+1)\times (n+1)$ matrix representing orthogonal projection of ${\mathbb {R}}^{n+1}$ onto *S*, that is

$$\begin{aligned} S^2=S, \quad S^TS=I \end{aligned}$$

and write $S_1\cdot S_2 = {{\,\textrm{tr}\,}}(S_1^T\cdot S_2)$. We say *V* is an *n*-varifold in $\Omega \subset {\mathbb {R}}^{n+1}$ if *V* is a Radon measure on $G_n(\Omega ) = \Omega \times G(n+1,n)$. Varifold convergence means convergence of Radon measures or weak-$*$ convergence. We let $V\in {\mathbb {V}}_n(\Omega )$ and let $\Vert V\Vert$ denote the weight measure of *V* and we define the first variation of *V* by

$$\begin{aligned} \delta V(\eta ) \equiv \int _{G_n(\Omega )}\nabla \eta (x) \cdot S dV(x,S) \quad \forall \eta \in C_c^1(\Omega ;{\mathbb {R}}^{n+1}). \end{aligned}$$

We let $\Vert \delta V\Vert$ be the total variation of $\delta V$. If $\Vert \delta V\Vert$ is absolutely continuous with respect to $\Vert \delta V\Vert$ then the Radon–Nikodym derivative $\frac{\delta V}{\Vert V\Vert }$ exists as vector valued measure. We denote by $H_V = -\frac{\delta V}{\Vert V\Vert }$, the generalised mean curvature.

Let $u=u_\varepsilon$ be a function in Theorem 1.1, we define the associated energy measure as a Radon measure given by

$$\begin{aligned} d\mu _\varepsilon \equiv \left( \frac{\varepsilon |\nabla u_\varepsilon |^2}{2}+ \frac{W(u_\varepsilon )}{\varepsilon }\right) d {\mathcal {L}}^{n+1} \end{aligned}$$

where ${\mathcal {L}}^{n+1}$ is the $(n+1)$ dimensional Lebesgue measure. We also denote the energy of the 1 dimensional solution by

$$\begin{aligned} \sigma = \int _{-1}^1\sqrt{2 W(s)} ds. \end{aligned}$$

There is an associated varifold $V \in {\mathbb {V}}_n(\Omega )$ to the functions *u* given by

$$\begin{aligned} V(\phi )&= \int _{\{| \nabla u |\ne 0\}} \phi \left( x,\left( \frac{\nabla u(x)}{|\nabla u(x)|}\right) ^\perp \right) d\mu (x) \\&= \int _{\{| \nabla u |\ne 0\}} \phi \left( x,I - \frac{\nabla u(x)}{|\nabla u(x)|}\otimes \frac{\nabla u(x)}{|\nabla u(x)|}\right) d\mu (x), \quad \phi \in C_c(G_n(\Omega )). \end{aligned}$$

where *I* is the $(n+1)\times (n+1)$ identity matrix and

$$\begin{aligned} I - \frac{\nabla u(x)}{|\nabla u(x)|}\otimes \frac{\nabla u(x)}{|\nabla u(x)|} \end{aligned}$$

is orthogonal projection onto the space orthogonal to $\nabla u(x)$, that is $\{a\in {\mathbb {R}}^{n+1}\mid \langle a, \nabla u(x)\rangle = 0 \}.$ By definition $\Vert V \Vert = \mu \llcorner _{\{|\nabla u| \ne 0 \} }$ and the first variation may be computed as

2.1 $$\begin{aligned} \delta V( \eta )&= \int _{\{| \nabla u |\ne 0\}} \nabla \eta \cdot \left( I - \frac{\nabla u(x)}{|\nabla u(x)|}\otimes \frac{\nabla u(x)}{|\nabla u(x)|}\right) d\mu (x), \forall \eta \in C_c^1(\Omega ; {\mathbb {R}}^{n+1}). \end{aligned}$$

## Discrepancy bounds and monotonicity formula

In this section, we deduce integral bounds on the discrepancy. There exists an almost monotonicity formula for the Allen–Cahn energy functional, we will give estimates on the terms appearing in the almost monotonicity formulas under the assumptions in Theorem 1.1 and obtain a monotonicity formula for the *n*-dimensional volume ratio. It will be used in the next section to deduce rectifiability and integrality of the limit varifold as $\varepsilon \rightarrow 0$. Conditions (1)–(3) in Theorem 1.1 are assumed to hold throughout this section.

The *n*-dimensional volume ratio of the energy measure satisfies the following almost monotonicity formula.

### Proposition 3.1

(Almost Monotonicity Formula) If $u_\varepsilon$ satisfies (1.1) in $B_1\subset {\mathbb {R}}^{n+1}$, then for $r<1$, we have

3.1 $$\begin{aligned} \frac{d}{dr}\left( \frac{\mu _\varepsilon (B_r)}{r^n}\right)&=-\frac{1}{r^{n+1}}\xi (B_r)+\frac{\varepsilon }{r^{n+2}}\int _{\partial B_r}\langle x,\nabla u_\varepsilon \rangle ^2\nonumber \\ {}&\quad -\frac{1}{r^{n+1}}\int _{B_r}\langle x,\nabla u_\varepsilon \rangle f_\varepsilon . \end{aligned}$$

Here $\mu _\varepsilon (B_r)=\int _{B_r}d\mu _\varepsilon =\int _{B_r}\left( \frac{\varepsilon |\nabla u_\varepsilon |^2}{2}+\frac{W(u_\varepsilon )}{\varepsilon }\right)$ is the total energy and $\xi (B_r)=\int _{B_r}\left( \frac{\varepsilon |\nabla u_\varepsilon |^2}{2}-\frac{W(u_\varepsilon )}{\varepsilon }\right)$ is the discrepancy measure (difference between the Dirichlet and potential energy) in $B_r$.

### Proof

Multiplying Eq. (1.1) by $\langle x,\nabla u_\varepsilon \rangle$ and integrating by parts on $B_r$, we get

$$\begin{aligned}&\int _{B_r}\langle x,\nabla u_\varepsilon \rangle f_\varepsilon =\int _{B_r}\varepsilon \Delta u_\varepsilon \langle x,\nabla u_\varepsilon \rangle -\int _{B_r}\left\langle \frac{\nabla (W(u_\varepsilon ))}{\varepsilon }, x\right\rangle \\&\quad =\int _{\partial B_r}\left( \varepsilon r\left| \frac{\partial u_\varepsilon }{\partial \nu }\right| ^2-r\frac{W(u_\varepsilon )}{\varepsilon }\right) \\&\qquad -\int _{B_r}\left( \varepsilon \delta _{ij}u_{x_i}u_{x_j}+\varepsilon \nabla ^2u(\nabla u_\varepsilon ,x)-\frac{(n+1)W(u_\varepsilon )}{\varepsilon }\right) \\&\quad =\int _{\partial B_r}\left( \varepsilon r\left| \frac{\partial u_\varepsilon }{\partial \nu }\right| ^2-r\frac{W(u_\varepsilon )}{\varepsilon }\right) \\&\qquad -\int _{B_r}\left( \varepsilon |\nabla u_\varepsilon |^2+\varepsilon \left\langle \nabla \frac{|\nabla u_\varepsilon |^2}{2}, x\right\rangle -\frac{(n+1)W(u_\varepsilon )}{\varepsilon }\right) \\&\quad =r\int _{\partial B_r}\left( \varepsilon \left| \frac{\partial u_\varepsilon }{\partial \nu }\right| ^2-\frac{W(u_\varepsilon )}{\varepsilon }-\varepsilon \frac{|\nabla u_\varepsilon |^2}{2}\right) \\&\qquad +\int _{B_r}\left( \varepsilon \frac{(n-1)|\nabla u_\varepsilon |^2}{2}+\frac{(n+1)W(u_\varepsilon )}{\varepsilon }\right) \\&\quad =n\int _{B_r}\left( \frac{\varepsilon |\nabla u_\varepsilon |^2}{2}+\frac{W(u_\varepsilon )}{\varepsilon }\right) -r\int _{\partial B_r}\left( \frac{\varepsilon |\nabla u_\varepsilon |^2}{2}+\frac{W(u_\varepsilon )}{\varepsilon }\right) \\&\qquad +\frac{\varepsilon }{r}\int _{\partial B_r}\langle x,\nabla u_\varepsilon \rangle ^2-\xi (B_r). \end{aligned}$$

The conclusion then follows by dividing both sides by $r^{n+1}$ and noticing

$$\begin{aligned} \frac{d}{dr}\left( \frac{\mu _\varepsilon (B_r)}{r^n}\right) =-\frac{n}{r^{n+1}}\int _{B_r}\left( \frac{\varepsilon |\nabla u_\varepsilon |^2}{2}+\frac{W(u_\varepsilon )}{\varepsilon }\right) +\frac{1}{r^n}\int _{\partial B_r}\left( \frac{\varepsilon |\nabla u_\varepsilon |^2}{2}+\frac{W(u_\varepsilon )}{\varepsilon }\right) . \end{aligned}$$

$$\square $$

Integrating the almost monotonicity formula (3.1) from $\varepsilon$ to $r_0$ for $0<\varepsilon<r_0<1$, we have

3.2 $$\begin{aligned}&\frac{\mu _\varepsilon (B_{r_0})}{r_0^n}-\frac{\mu _\varepsilon (B_\varepsilon )}{\varepsilon ^n}\nonumber \\&\quad =\int _\varepsilon ^{r_0}\left( -\frac{1}{r^{n+1}}\xi (B_r)+\frac{\varepsilon }{r^{n+2}}\int _{\partial B_r}\langle x,\nabla u_\varepsilon \rangle ^2-\frac{1}{r^{n+1}}\int _{B_r}\langle x,\nabla u_\varepsilon \rangle f_\varepsilon \right) dr\nonumber \\&\quad \ge -r_0\sup _{B_{r_0}}\omega _{n+1}\left( \frac{\varepsilon |\nabla u_\varepsilon |^2}{2}-\frac{W(u_\varepsilon )}{\varepsilon }\right) _+ + \int _{B_{r_0}\setminus B_\varepsilon }\frac{\varepsilon \langle x,\nabla u_\varepsilon \rangle ^2}{|x|^{n+2}}\nonumber \\&\qquad -\int _\varepsilon ^{r_0}\frac{1}{r^{n+1}}\int _{B_r}\langle x,\nabla u_\varepsilon \rangle f_\varepsilon dr, \end{aligned}$$

where $\omega _{n+1}$ denotes the volume of unit ball in ${\mathbb {R}}^{n+1}$.

We need to estimate the first and third term on the right hand side to obtain a monotonicity formula. In order to estimate the third term, we derive an a priori gradient bound for *u*. Condition (3) of Theorem 1.1 states a combined integrability for the inhomogeneity $f_\varepsilon$ and $|\nabla u|$. The following theorem allows us to obtain separate integrability and regularity for each quantity.

### Theorem 3.2

There exists $C, \varepsilon _0>0$ depending on $E_0,c_0,\Lambda _0$ as defined in Theorem 1.1 such that if $u_\varepsilon$ satisfies (1.1) in $B_1\subset {\mathbb {R}}^{n+1}$ with $\varepsilon <\varepsilon _0$ and if $q_0>n+1$, then

3.3 $$\begin{aligned} \sup _{B_{1-\varepsilon }}\varepsilon |\nabla u_\varepsilon |\le C, \end{aligned}$$

and

3.4 $$\begin{aligned} \varepsilon ^{2-\frac{n+1}{q_0}}\Vert u_\varepsilon \Vert _{C^{1,1-\frac{n+1}{q_0}}(B_{1-\varepsilon })}\le C. \end{aligned}$$

If $$n<q_0\le n+1$$, then

3.5 $$\begin{aligned} \varepsilon ^\frac{1}{2}\Vert u_\varepsilon \Vert _{C^{0,\frac{1}{2}}(B_{1-\varepsilon })}\le C. \end{aligned}$$

Furthermore, there exists a $\delta _0>0$ so that *f* has the following improved integrability

3.6 $$\begin{aligned} \Vert f_\varepsilon \Vert _{L^{\frac{n+1}{2}+\delta _0}(B_{1-\varepsilon }(x_0))}\le C\varepsilon ^{-\frac{n}{q_0}}. \end{aligned}$$

### Proof

We first consider the case $q_0>n+1$: Define the rescaled solution ${\tilde{u}}(x):=u(\varepsilon x)$ and ${\tilde{f}}(x)=\varepsilon f_\varepsilon (\varepsilon x)$ which satisfies the equation

3.7 $$\begin{aligned} \Delta {\tilde{u}}-W'({\tilde{u}})={\tilde{f}},\quad \text { in } B_\frac{1}{\varepsilon }\subset {\mathbb {R}}^{n+1}. \end{aligned}$$

By condition (3) in Theorem 1.1, we have by rescaling

3.8 $$\begin{aligned} \int _{B_\frac{1}{\varepsilon }}{\tilde{f}}^{q_0}\varepsilon ^{n-q_0}|\nabla {\tilde{u}}|^{2-q_0}&=\int _{B_{\frac{1}{\varepsilon }}}\varepsilon ^{-2q_0}{\tilde{f}}^{q_0}\varepsilon |\nabla {\tilde{u}}|^{2-q_0}\varepsilon ^{q_0-2}\varepsilon ^{n+1}\nonumber \\&=\int _{B_1}\varepsilon ^{-q_0}f^{q_0}\varepsilon |\nabla u|^{2-q_0}\le \Lambda _0. \end{aligned}$$

$$\square $$

### Claim

For any ${\bar{B}}_1(x_0)\subset B_{\frac{1}{\varepsilon }-1}$, we have

$$\begin{aligned} \Vert \nabla {\tilde{u}}\Vert _{L^2(B_1(x_0))}\le C(c_0,\Lambda _0,q_0,n). \end{aligned}$$

### Proof of Claim

By the hypothesis ${\bar{B}}_1(x_0)\subset B_{\frac{1}{\varepsilon }-1}$ we have $B_2(x_0)\subset B_\frac{1}{\varepsilon }$. We choose a smooth cutoff function $\phi \in C_c^\infty \left( B_2(x_0)),[0,1]\right)$ with $\phi \equiv 1$ in $B_1(x_0)$ and $|\nabla \phi |\le 4$. By integration by parts and Young’s inequality, we obtain

3.9 $$\begin{aligned} \begin{aligned}&\int _{B_2(x_0)}|\nabla {\tilde{u}}|^2\phi ^2 \le \int _{B_2(x_0)}2c_0|\nabla {\tilde{u}}||\phi ||\nabla \phi |+\int _{B_2(x_0)}c_0\phi ^2|\Delta {\tilde{u}}|\\&\quad \le \int _{B_2(x_0)}2c_0|\nabla {\tilde{u}}||\phi ||\nabla \phi |+\int _{B_2(x_0)}c_0\phi ^2|W'({\tilde{u}})|\\&\qquad +\int _{B_2(x_0)}c_0\phi ^2|{\tilde{f}}|\\&\quad \le \frac{1}{2}\int _{B_2(x_0)}|\nabla {\tilde{u}}|^2\phi ^2 +\int _{B_2(x_0)}2c_0^2|\nabla \phi |^2+\int _{B_2(x_0)}c_0\phi ^2 C_{c_0}+\int _{B_2(x_0)}c_0\phi ^2|{\tilde{f}}|. \end{aligned}\nonumber \\ \end{aligned}$$

We write $c_0 \phi ^2 | {\tilde{f}}| = c_0| {\tilde{f}} |\varepsilon ^{\frac{n}{q_0}-1}|\nabla {\tilde{u}}|^{\frac{2}{q_0}-1} \times \phi ^2\varepsilon ^{1-\frac{n}{q_0}}|\nabla {\tilde{u}}|^{1-\frac{2}{q_0}}$ and use Young’s inequality with exponent $q_0$ to get

$$\begin{aligned} \int _{B_2(x_0)}c_0\phi ^2|{\tilde{f}}|&\le \frac{1}{\delta q_0}\int _{B_2(x_0)}\left| c_0|{\tilde{f}}| \varepsilon ^{\frac{n}{q_0}-1}|\nabla {\tilde{u}}|^{\frac{2}{q_0}-1}\right| ^{q_0}\\&\quad +\frac{\delta (q_0-1)}{q_0}\int _{B_2(x_0)}\left| \phi ^2\varepsilon ^{1-\frac{n}{q_0}}|\nabla {\tilde{u}}|^{1-\frac{2}{q_0}}\right| ^\frac{q_0}{q_0-1}\\&\le \frac{c_0^{q_0}}{\delta q_0}\Lambda _0+\frac{\delta (q_0-1)}{q_0}\int _{B_2(x_0)}\phi ^\frac{2q_0}{q_0-1}|\nabla {\tilde{u}}|^\frac{q_0-2}{q_0-1}\\&\le \frac{c_0^{q_0}}{\delta q_0}\Lambda _0+\frac{C_n\delta (q_0-1)}{q_0}\left( \int _{B_2(x_0)}\phi ^\frac{4q_0}{q_0-2}|\nabla {\tilde{u}}|^2\right) ^\frac{q_0-2}{2(q_0-1)}\\&\le \frac{4C_n(q_0-1)c_0^n}{q_0^2}\Lambda _0+\frac{1}{4}\max \left\{ \left[ \int _{B_2(x_0)}\phi ^2|\nabla {\tilde{u}}|^2\right] ^\frac{4q_0}{q_0-2},1\right\} . \end{aligned}$$

Here we used (3.8) to bound $\int _{B_2(x_0)}\left| c_0{\tilde{f}}\varepsilon ^{\frac{n}{q_0}-1}|\nabla {\tilde{u}}|^{\frac{2}{q_0}-1}\right| ^{q_0}$ and the fact that $\varepsilon ^{1-\frac{n}{q_0}}<1$ in the second inequality, Hölder’s inequality with exponent $\frac{2(q_0-1)}{q_0-2}$ in the third inequality. And the fourth inequality is obtained from the third by choosing $\delta$ to be $\frac{q_0}{4C_n(q_0-1)}$. We assume $\int _{B_2(x_0)}\phi ^2|\nabla {\tilde{u}}|^2\ge 1$, otherwise the desired bound holds trivially. Inserting the above inequality into (3.9), we get

$$\begin{aligned} \int _{B_2(x_0)}|\nabla {\tilde{u}}|^2\phi ^2&\le \frac{1}{2}\int _{B_2(x_0)}|\nabla {\tilde{u}}|^2\phi ^2+\int _{B_2(x_0)}2c_0^2|\nabla \phi |^2+\int _{B_2(x_0)}c_0\phi ^2 C_{c_0}\\&\quad +\frac{4C_n(q_0-1)c_0^n}{q_0^2}\Lambda _0+\frac{1}{4}\max \left\{ \int _{B_2(x_0)}\phi ^2|\nabla {\tilde{u}}|^2,1\right\} . \end{aligned}$$

Then by moving the first term $\frac{1}{2}\int _{B_2(x_0)}|\nabla {\tilde{u}}|^2\phi ^2$ and the fifth term $\int _{B_2(x_0)}\phi ^2|\nabla {\tilde{u}}|^2$ on the right to the left, we prove the claim. $\square$

Now suppose $\Vert \nabla {\tilde{u}}\Vert _{L^{p_0}(B_1(x_0))}\le C(c_0,\Lambda _0,q_0,n)$ (independent of $\varepsilon$) for some $p_0>1$ ($p_0$ can be chosen to be 2 by the claim above). For any $B_2(x_0)\in B_{\frac{1}{\varepsilon }}(0)$, we have by Hölder’s inequality

3.10 $$\begin{aligned} \Vert {\tilde{f}}\Vert _{L^\frac{p_0q_0}{p_0+q_0-2}(B_1(x_0))}&=\left( \int _{B_1(x_0)} |{\tilde{f}}|^\frac{p_0q_0}{p_0+q_0-2}\right) ^\frac{p_0+q_0-2}{p_0q_0}\nonumber \\&\quad \le \left[ \left\| \left| {\tilde{f}}\varepsilon ^{\frac{n-q_0}{q_0}}|\nabla {\tilde{u}}|^{\frac{2}{q_0}-1}\right| ^\frac{p_0q_0}{p_0+q_0-2}\right\| _{L^\frac{p_0+q_0-2}{p_0}(B_1(x_0))}\right. \nonumber \\&\qquad \left. \cdot \left\| \left( \varepsilon ^{\frac{q_0-n}{q_0}}|\nabla {\tilde{u}}|^{1-\frac{2}{q_0}}\right) ^\frac{p_0q_0}{p_0+q_0-2}\right\| _{L^\frac{p_0+q_0-2}{q_0-2}(B_1(x_0))}\right] ^\frac{p_0+q_0-2}{p_0q_0}\nonumber \\&\quad \le \left[ \Lambda _0^\frac{p_0}{p_0+q_0-2}\varepsilon ^\frac{(q_0-n)p_0}{p_0+q_0-2}\cdot \left( \int _{B_1(x_0)}|\nabla {\tilde{u}}|^{p_0}\right) ^\frac{q_0-2}{p_0+q_0-2}\right] ^\frac{p_0+q_0-2}{p_0q_0}\nonumber \\&\quad =\Lambda _0^\frac{1}{q_0}\cdot \varepsilon ^\frac{q_0-n}{q_0}\cdot \left( \int _{B_1(x_0)}|\nabla {\tilde{u}}|^{p_0}\right) ^\frac{q_0-2}{p_0q_0}\nonumber \\&\quad \le C(c_0,\Lambda _0,q_0,n)\varepsilon ^\frac{q_0-n}{q_0}\le C(c_0,\Lambda _0,q_0,n). \end{aligned}$$

### Remark 3.3

Here $q_0>n$ will make the scaling subcritical and ensures a uniform bound of $\Vert {\tilde{f}}\Vert _{L^\frac{p_0q_0}{p_0+q_0-2}(B_1(x_0))}$ independent of $\varepsilon$.

Thus ${\tilde{f}}$ is uniformly bounded in $L^\frac{p_0q_0}{p_0+q_0-2}(B_1(x_0))$ independent of $\varepsilon$. By applying the Sobolev inequality to (3.7), standard Calderon–Zygmund estimates and finally using the $L^\infty$ bound of *u* in condition (2) of Theorem 1.1, we have

3.11 $$\begin{aligned} \Vert \nabla {\tilde{u}}\Vert _{L^{\frac{p_0q_0}{p_0+q_0-2-p_0\frac{q_0}{n+1}}}({B_1(x_0)})}&\le \Vert {\tilde{u}}\Vert _{W^{1,\frac{p_0q_0}{p_0+q_0-2-p_0\frac{q_0}{n+1}}}({B_1(x_0)})}\nonumber \\&\le C\Vert {\tilde{u}}\Vert _{W^{2,\frac{p_0q_0}{p_0+q_0-2}}(B_1(x_0))}\nonumber \\&\le C\Vert {\tilde{f}}\Vert _{L^\frac{p_0q_0}{p_0+q_0-2}(B_1(x_0))}+C\Vert W'({\tilde{u}})\Vert _{L^\frac{p_0q_0}{p_0+q_0-2}(B_1(x_0))}\nonumber \\&\le C\Lambda _0^\frac{1}{q_0}\cdot \varepsilon ^\frac{q_0-n}{q_0}\cdot \left( \int _{B_1(x_0)}|\nabla {\tilde{u}}|^{p_0}\right) ^\frac{q_0-2}{p_0q_0}\nonumber \\&\quad +C\Vert W'({\tilde{u}})\Vert _{L^\infty (B_1(x_0))}\nonumber \\&\le C(c_0,\Lambda _0,q_0,n)\left( \varepsilon ^\frac{q_0-n}{q_0}+1\right) \le {\tilde{C}}(c_0,\Lambda _0,q_0,n). \end{aligned}$$

We remark that $$ q_0 > n$$ ensures the coefficient $$\varepsilon ^\frac{q_0-n}{q_0}$$ stays uniformly bounded as $$ \varepsilon \rightarrow 0$$.

In the case $\frac{p_0q_0}{p_0+q_0-2}>n+1$, by Calderon–Zygmund estimates we have

$$\begin{aligned} \Vert {\tilde{u}}\Vert _{W^{2,\frac{p_0q_0}{p_0+q_0-2}}(B_1(x_0))}\le C(c_0,\Lambda _0,q_0,n)\left( \varepsilon ^\frac{q_0-n}{q_0}+1\right) \le {\tilde{C}}(c_0,\Lambda _0,q_0,n). \end{aligned}$$

The Sobolev inequality then gives $\Vert \nabla {\tilde{u}}\Vert _{L^\infty }\le C$.

In the case $\frac{p_0q_0}{p_0+q_0-2}\le n+1$, using $q_0>n+1$, we have $p_0<p_0\frac{q_0}{n+1}$. Namely

3.12 $$\begin{aligned} \frac{q_0}{p_0+q_0-2-p_0\frac{q_0}{n+1}}p_0=\frac{q_0}{(p_0-p_0\frac{q_0}{n+1})+(q_0-2)}p_0\ge \frac{q_0}{q_0-2}p_0. \end{aligned}$$

So we improved $\nabla {\tilde{u}}$ from $L^{p_0}$ to $L^{\frac{q_0}{q_0-2}p_0}$. Define $p_i=\frac{q_0}{q_0-2}p_{i-1}$. Using $q_0>n+1$, we iterate finitely many times until $p_i>\frac{(n+1)(q_0-2)}{q_0-(n+1)}$, i.e. $\frac{p_iq_0}{p_i+q_0-2}>n+1$. The Sobolev inequality gives $\nabla {\tilde{u}}\in L^\infty$. So if $q_0>n+1$, we get $\nabla {\tilde{u}}\in L^\infty$. Rescaling back, we get (3.3). By (3.8) where $(q_0> n+1\ge 2)$ and $\nabla {\tilde{u}}\in L^\infty$, we have ${\tilde{f}}\in L^{q_0}$. Standard Calderon–Zygmund estimates give

$$\begin{aligned} \Vert \nabla {\tilde{u}}\Vert _{C^{0,1-\frac{n+1}{q_0}}(B_1(x_0))}&\le \Vert {\tilde{u}}\Vert _{W^{2,q_0}(B_1(x_0))}\le \Vert {\tilde{f}}\Vert _{L^{q_0}(B_1(x_0))}\\ {}&\quad +\Vert W'({\tilde{u}})\Vert _{L^{q_0}(B_1(x_0))}<\infty , \end{aligned}$$

which gives (3.4).

Consider now the case $n<q_0\le n+1$. For any

3.13 $$\begin{aligned} p_i\le \frac{2(n+1)}{n+1-q_0}-\delta , \end{aligned}$$

we have

$$\begin{aligned} p_i+q_0-2-p_i\frac{q_0}{n+1}&=p_i\frac{n+1-q_0}{n+1}+q_0-2\\&=\left( \frac{2(n+1)}{n+1-q_0}-\delta \right) \frac{n+1-q_0}{n+1}+q_0-2\\&=q_0-\frac{n+1-q_0}{n+1}\delta . \end{aligned}$$

And thus

3.14 $$\begin{aligned} \frac{q_0}{p_i+q_0-2-p_i\frac{q_0}{n+1}}p_i\ge \frac{q_0}{q_0-\frac{n+1-q_0}{n+1}\delta }p_i\ge p_i. \end{aligned}$$

So (3.11) increases the integrability of $\nabla {\tilde{u}}$ from $L^{p_i}$ to $L^{\frac{q_0}{q_0-\frac{n+1-q_0}{n+1}\delta }p_i}$. And we can iterate until (3.13) fails, namely

3.15 $$\begin{aligned} \Vert \nabla {\tilde{u}}\Vert _{L^{\frac{2(n+1)}{n+1-q_0}-\delta }(B_1(x_0))}\le C(c_0,\Lambda _0,q_0,n)\varepsilon ^\frac{q_0-n}{q_0}\le C(c_0,\Lambda _0,q_0,n), \end{aligned}$$

for any $x_0\in B_{\frac{1}{\varepsilon }-2}$(so that the condition in the claim above is satisfied). By Sobolev inequalities, we then have for any $x_0\in B_{\frac{1}{\varepsilon }-2}$

$$\begin{aligned} \Vert {\tilde{u}}\Vert _{C^{0,\frac{1}{2}}(B_1(x_0))}&\le C\Vert {\tilde{u}}\Vert _{W^{1,2(n+1)}(B_1(x_0))}\\&\le C\Vert {\tilde{u}}\Vert _{W^{1,\frac{2(n+1)}{n+1-q_0}-\delta }(B_1(x_0))}\\&\le C(c_0,\Lambda ,0,q_0,n)\varepsilon ^\frac{q_0-n}{q_0}\le C(c_0,\Lambda ,0,q_0,n). \end{aligned}$$

Rescaling back gives

$$\begin{aligned} \varepsilon ^\frac{1}{2}\Vert u\Vert _{C^{0,\frac{1}{2}}(B_{1-\varepsilon })}\le \Vert {\tilde{u}}\Vert _{C^{0,\frac{1}{2}}\big (B_{\frac{1}{\varepsilon }-1}\big )}\le C(c_0,\Lambda _0,q_0,n)\varepsilon ^\frac{q_0-n}{q_0}\le C(c_0,\Lambda _0,q_0,n), \end{aligned}$$

which is (3.5). By (3.10) we improve the integrability of ${\tilde{f}}$ in (3.10) up to

$$\begin{aligned} \Vert {\tilde{f}}\Vert _{L^\frac{p_iq_0}{p_i+q_0-2}(B_1(x_0))}\le C\varepsilon ^\frac{q_0-n}{q_0}, \end{aligned}$$

for $p_i\le \frac{2(n+1)}{n+1-q_0}-\delta$. So if $q_0\in (n,n+1]$, by choosing $p_i=2(n+1)$, we have

3.16 $$\begin{aligned} \frac{p_iq_0}{p_i+q_0-2}=\frac{p_i}{\frac{p_i-2}{q_0}+1}>\frac{p_i}{\frac{p_i-2}{n}+1}=\frac{2(n+1)}{\frac{2(n+1)-2}{n}+1}=\frac{2(n+1)}{3}, \end{aligned}$$

rearranging gives $\frac{p_iq_0}{p_i+q_0-2}>\frac{2(n+1)}{3}\ge \frac{n+1}{2}+\delta _0$ for some $\delta _0>0$. On the other hand, if $q_0>n+1$, using (3.8) and the uniform gradient bound of *u* in Theorem 3.2, we have $\Vert {\tilde{f}}\Vert _{L^{q_0}(B_1(x_0))}\le C\varepsilon ^\frac{q_0-n}{q_0}$, where $q_0>n+1>\frac{n+1}{2}+\delta _0$. Combining both cases, for any $q_0>n$

3.17 $$\begin{aligned} \Vert {\tilde{f}}\Vert _{L^{\frac{n+1}{2}+\delta _0}(B_1(x_0))}\le C\varepsilon ^\frac{q_0-n}{q_0}. \end{aligned}$$

and

3.18 $$\begin{aligned} \Vert {\tilde{f}}\Vert _{L^{\frac{n+1}{2}+\delta _0}\big (B_{\frac{1}{\varepsilon }-1}(x_0)\big )}\le C\varepsilon ^\frac{q_0-n}{q_0}\varepsilon ^{-n-1}, \end{aligned}$$

Rescaling back gives the bound on *f*,

$$\begin{aligned} \Vert f\Vert _{L^{\frac{n+1}{2}+\delta _0}(B_{1-\varepsilon }(x_0))}\le C\varepsilon ^{-\frac{n}{q_0}}. \end{aligned}$$

$$\square $$

Since in the case $q_0\in (n,n+1]$, we lack gradient bounds of *u* as in the case $q_0>n+1$. In order to get better estimates of the discrepancy terms in the almost monotonicity formula, we use some ideas from [8]. We will apply the following Lemma to (3.7) for $\varepsilon$ sufficiently small such that $C\varepsilon ^\frac{q_0-n}{q_0}\le \omega$.

### Lemma 3.4

(cf [8, Lemma 3.2]) Let $n+1\ge 3, 0<\delta \le \delta _1$ and $R(\delta )= \frac{1}{\delta ^{p_1}}, \omega (\delta ) = \delta ^{p_2}$, where $p_1=5, p_2=35$. If ${\tilde{u}}\in C^2(B_R), {\tilde{f}}\in C^0(B_R), B_R=B_R(0)\subset {\mathbb {R}}^{n+1}$ where

$$\begin{aligned} \begin{array}{cc} -\Delta {\tilde{u}} +W'({\tilde{u}})={\tilde{f}}&{}\text { in }B_R, \\ |{\tilde{u}}| \le c_0&{}\text { in }B_R, \\ \Vert {\tilde{f}}\Vert _{L^{\frac{n+1}{2}+\delta _0}(B_R)}\le \omega , \end{array} \end{aligned}$$

$c_0$ is as assumed in condition (2) of Theorem 1.1 and $\delta _0$ is as in Theorem 3.2. Then

3.19 $$\begin{aligned} \int _{B_1} \left( \frac{|\nabla {\tilde{u}}|^2}{2} - W({\tilde{u}})\right) _+ \le C\delta . \end{aligned}$$

And for $\tau = \delta ^{p_3}$, where $p_3=\frac{2\delta _0}{(n+1)^2+(n+1)\delta _0+6\delta _0}$ , we get

3.20 $$\begin{aligned} \int _{B_{\frac{1}{2}}}\left( \frac{|\nabla {\tilde{u}}|^2}{2} - W({\tilde{u}}) \right) _+&\le c\tau \int _{B_{\frac{1}{2}}}\left( \frac{|\nabla {\tilde{u}}|^2}{2} + W({\tilde{u}}) \right) \nonumber \\&\quad +\int _{B_{\frac{1}{2}}\cap \{|{\tilde{u}}| \ge 1- \tau \} }\frac{|\nabla {\tilde{u}}|^2}{2}. \end{aligned}$$

### Proof

Let us consider the auxiliary function $\psi$ which solves the Dirichlet problem

3.21 $$\begin{aligned} \begin{array}{*{20}l} {\Delta \psi = - \tilde{f},} &{} {\quad {\text { in }}B_R } \\ {\psi = 0,} &{} {\quad {\text { on }}\partial B_R .} \\ \end{array} \end{aligned}$$

The auxiliary function will allows us to control the inhomogeneous part of the equation. $\square$

### Claim

The function $\psi$ defined in (3.21) satisfies the bounds

3.22 $$\begin{aligned} \Vert \psi \Vert _{L^\infty (B_R)}&\le C\delta ^{25+5\frac{n+1}{\frac{n+1}{2}+\delta _0}}\ll 1, \end{aligned}$$

3.23 $$\begin{aligned} \Vert \nabla \psi \Vert _{L^\frac{(n+1)(n+1+2\delta _0)}{n+1-2\delta _0}(B_R)}&\le C\omega =C\delta ^{35}. \end{aligned}$$

### Proof

Rescaling by $\frac{1}{R}$, we have

3.24 $$\begin{aligned} \begin{array}{ll} \Delta \psi _R={\tilde{f}}_R,&{} \quad \text { in }B_1\\ \psi _R=0, &{}\quad \text { on }\partial B_1, \end{array} \end{aligned}$$

where $\psi _R(x)=\psi (Rx), {\tilde{f}}_R(x)=R^2{\tilde{f}}(Rx)$. Standard Calderon–Zygmund estimates give

$$\begin{aligned} \Vert \psi _R\Vert _{W^{2,\frac{n+1}{2}+\delta _0}(B_1)}&\le \Vert {\tilde{f}}_R\Vert _{L^{\frac{n+1}{2}+\delta _0}(B_1)}=R^{2-\frac{n+1}{\frac{n+1}{2}+\delta _0}}\Vert {\tilde{f}}\Vert _{L^{\frac{n+1}{2}+\delta _0}(B_R)}\\&\le CR^{2-\frac{n+1}{\frac{n+1}{2}+\delta _0}}\omega , \end{aligned}$$

where $2-\frac{n+1}{\frac{n+1}{2}+\delta _0}>0$. Rescaling back yields

$$\begin{aligned}&\Vert \psi \Vert _{L^{\frac{n+1}{2}+\delta _0}(B_R)}+R\Vert \nabla \psi \Vert _{L^{\frac{n+1}{2}+\delta _0}(B_R)}+R^2\Vert \nabla ^2\psi \Vert _{L^{\frac{n+1}{2}+\delta _0}(B_R)}\\&\quad =R^\frac{n+1}{\frac{n+1}{2}+\delta _0}\Vert \psi _R\Vert _{L^{\frac{n+1}{2}+\delta _0}(B_1)}+R^\frac{n+1}{\frac{n+1}{2}+\delta _0}\Vert \nabla \psi _R\Vert _{L^{\frac{n+1}{2}+\delta _0}(B_1)}\\&\qquad +R^\frac{n+1}{\frac{n+1}{2}+\delta _0}\Vert \nabla ^2\psi _R\Vert _{L^{\frac{n+1}{2}+\delta _0}(B_1)}\\&\quad = R^\frac{n+1}{\frac{n+1}{2}+\delta _0}\Vert \psi _R\Vert _{W^{2,\frac{n+1}{2}+\delta _0}(B_1)}\\&\quad \le CR^\frac{n+1}{\frac{n+1}{2}+\delta _0}R^{2-\frac{n+1}{\frac{n+1}{2}+\delta _0}}\omega \\&\quad =CR^2\omega \\&\quad = C\delta ^{25}. \end{aligned}$$

*Here we prove* (3.22): by the Sobolev inequality since $\delta _0>0 \implies \frac{n+1}{2} + \delta _0 >\frac{n+1}{2}$, we have

$$\begin{aligned} \Vert \psi \Vert _{L^\infty (B_R)}&=\Vert \psi _R\Vert _{L^\infty (B_1)}\le C\Vert \psi _R\Vert _{W^{2,\frac{n+1}{2}+\delta _0}(B_1)}\\ {}&\le CR^{2-\frac{n+1}{\frac{n+1}{2}+\delta _0}}\omega \\&=C\delta ^{25+5\frac{n+1}{\frac{n+1}{2}+\delta _0}}\ll 1, \end{aligned}$$

due to the choice of $\omega$, where we used $\frac{(n+1)(n+1+2\delta _0)}{n+1-2\delta _0}>n+1$. *Here we prove the gradient bound* (3.23):

$$\begin{aligned} \Vert \nabla \psi \Vert _{L^\frac{(n+1)(n+1+2\delta _0)}{n+1-2\delta _0}(B_R)}&\le R^{\frac{n+1-2\delta _0}{n+1+2\delta _0}-1}\Vert \nabla \psi _R\Vert _{L^\frac{(n+1)(n+1+2\delta _0)}{n+1-2\delta _0}(B_1)}\\&\le CR^{\frac{n+1-2\delta _0}{n+1+2\delta _0}-1}\Vert \psi _R\Vert _{W^{2,\frac{n+1}{2}+\delta _0}(B_1)}\\&\le CR^{\frac{n+1-2\delta _0}{n+1+2\delta _0}-1}R^{2-\frac{n+1}{\frac{n+1}{2}+\delta _0}}\omega \\&=CR^0\omega \\&=C\omega =C\delta ^{35}. \end{aligned}$$

$$\square $$

We define ${\tilde{u}}_0:={\tilde{u}}+\psi \in W^{2,\frac{n+1}{2}+\delta _0}(B_R)$. By (3.21), (3.22), ${\tilde{u}}_0$ satisfies

3.25 $$\begin{aligned} |{\tilde{u}}_0|&\le c_0+1,\nonumber \\ \Delta {\tilde{u}}_0&=W'({\tilde{u}}). \end{aligned}$$

We compute for any $\beta >0$,

$$\begin{aligned} \frac{|\nabla {\tilde{u}}|^2}{2}-W({\tilde{u}})&=\frac{|\nabla {\tilde{u}}_0-\nabla \psi |^2}{2}-W({\tilde{u}}_0-\psi )\\&\le \left( \frac{1}{2}+\beta \right) |\nabla {\tilde{u}}_0|^2+\left( \frac{1}{2}+\frac{1}{\beta }\right) |\nabla {\tilde{\psi }}|^2-W({\tilde{u}}_0)+C|\psi |, \end{aligned}$$

for some $C>0$. Thus by (3.22) and (3.23), we have

$$\begin{aligned}&\int _{B_1}\left( \frac{|\nabla {\tilde{u}}|^2}{2}-W({\tilde{u}})\right) _+\\&\quad \le \int _{B_1}\left( \frac{|\nabla {\tilde{u}}_0|^2}{2}-W({\tilde{u}}_0)\right) _+ + \int _{B_1}\left( \beta |\nabla {\tilde{u}}_0|^2+C|\psi |+\left( \frac{1}{2}+\frac{1}{\beta }\right) |\nabla \psi |^2\right) \\&\quad \le \int _{B_1}\left( \frac{|\nabla {\tilde{u}}_0|^2}{2}-W({\tilde{u}}_0)\right) _++C\left( \beta +R^{2-\frac{n+1}{\frac{n+1}{2}+\delta _0}}\omega +\left( \frac{1}{2}+\frac{1}{\beta }\right) \omega ^2\right) . \end{aligned}$$

By choosing $\beta =\omega \le \delta ^{p_2}$ and using our hypothesis on $\omega : R^{2-\frac{n+1}{\frac{n+1}{2}+\delta _0}}\omega =\delta ^{25+\frac{5(n+1)}{\frac{n+1}{2}+\delta _0}}$. By our choice of $p_1=2,p_2=15$, we ensure

$$\begin{aligned} \beta&=\delta ^{35}\le C\delta ,\\ R^{2-\frac{n+1}{\frac{n+1}{2}+\delta _0}}\omega&=\delta ^{25+5\frac{n+1}{\frac{n+1}{2}+\delta _0}}\le C\delta ,\\ \left( \frac{1}{2}+\frac{1}{\beta }\right) \omega ^2&=\frac{1}{2}\delta ^{70}+\delta ^{35}\le C\delta , \end{aligned}$$

for $$n\ge 2$$. Thus

$$\begin{aligned} \int _{B_1}\left( \frac{|\nabla {\tilde{u}}|^2}{2}-W({\tilde{u}})\right) _+\le \int _{B_1}\left( \frac{|\nabla {\tilde{u}}_0|^2}{2}-W({\tilde{u}}_0)\right) _+ +C\delta . \end{aligned}$$

To prove (3.19), it suffices to show

3.26 $$\begin{aligned} \int _{B_1}\left( \frac{|\nabla {\tilde{u}}_0|^2}{2}-W({\tilde{u}}_0)\right) _+\le C\delta . \end{aligned}$$

Here we estimate ${\tilde{u}}$. Define ${\tilde{u}}_R(x)={\tilde{u}}(Rx)$ then by the Calderon–Zygmund estimates we have

3.27 $$\begin{aligned} \Vert {\tilde{u}}_R\Vert _{W^{2,{\frac{n+1}{2}+\delta _0}}(B_\frac{1}{2})}&\le C\Vert \Delta {\tilde{u}}_R\Vert _{L^{\frac{n+1}{2}+\delta _0}(B_1)}+C\Vert {\tilde{u}}_R\Vert _{L^{\frac{n+1}{2}+\delta _0}(B_1)}\nonumber \\&\le C\left( R^{2-\frac{n+1}{\frac{n+1}{2}+\delta _0}}\Vert \Delta {\tilde{u}}\Vert _{L^{\frac{n+1}{2}+\delta _0}(B_R)}+1\right) \nonumber \\&\le C\left( R^{2-\frac{n+1}{\frac{n+1}{2}+\delta _0}}\left( \Vert W'({\tilde{u}})\Vert _{L^{\frac{n+1}{2}+\delta _0}(B_R)}+\Vert {\tilde{f}}\Vert _{L^{\frac{n+1}{2}+\delta _0}(B_R)}\right) +1\right) \nonumber \\&\le C\left( R^{2-\frac{n+1}{\frac{n+1}{2}+\delta _0}}(R^\frac{n+1}{\frac{n+1}{2}+\delta _0}+\omega )+1\right) \nonumber \\&\le CR^2. \end{aligned}$$

By the Sobolev embedding

3.28 $$\begin{aligned} \Vert \nabla {\tilde{u}}\Vert _{L^\frac{(n+1)(n+1+2\delta _0)}{n+1-2\delta _0}(B_\frac{R}{2})}&\le R^{\frac{n+1-2\delta }{n+1+2\delta }-1}\Vert \nabla {\tilde{u}}_R\Vert _{L^\frac{(n+1)(n+1+2\delta _0)}{n+1-2\delta _0}(B_\frac{1}{2})}\nonumber \\&\le R^{\frac{n+1-2\delta _0}{n+1+2\delta _0}-1}\Vert {\tilde{u}}_R\Vert _{W^{2,{\frac{n+1}{2}+\delta _0}}(B_\frac{1}{2})}\nonumber \\&\le R^{\frac{n+1-2\delta _0}{n+1+2\delta _0}-1}\cdot CR^2=C R^{\frac{n+1-2\delta }{n+1+2\delta }+1}. \end{aligned}$$

We define

$$\begin{aligned} {\tilde{f}}_0&:=-\Delta {\tilde{u}}_0+W'({\tilde{u}}_0)\\&=-\Delta \psi -\Delta {\tilde{u}}+W'({\tilde{u}})+W'^\prime ({\tilde{u}})\psi +\frac{1}{2}W^{(3)}({\tilde{u}})\psi ^2+\frac{1}{6}W^{(4)}({\tilde{u}})\psi ^3\\&=W'^\prime ({\tilde{u}})\psi +\frac{1}{2}W^{(3)}({\tilde{u}})\psi ^2+\frac{1}{6}W^{(4)}({\tilde{u}})\psi ^3, \end{aligned}$$

since the derivatives of order 5 or higher of the potential $W(u)=\frac{(1-u^2)^2}{2}$ vanish. By (3.22), (3.23) and (3.28), we have

3.29 $$\begin{aligned} \Vert {\tilde{f}}_0\Vert _{L^\infty (B_R)}\le C\Vert {\tilde{\psi }}\Vert ^3_{L^\infty (B_R)}\le C\left( R^{2-\frac{n+1}{\frac{n+1}{2}+\delta _0}}\omega \right) ^3\le C\delta ^{75+15\frac{n+1}{\frac{n+1}{2}+\delta _0}}\ll 1, \end{aligned}$$

and

3.30 $$\begin{aligned}&\Vert \nabla {\tilde{f}}_0\Vert _{L^\frac{(n+1)(n+1+2\delta _0)}{n+1-2\delta _0}(B_R)}\nonumber \\&\quad \le C\left( \Vert \nabla {\tilde{u}}\Vert _{L^\frac{(n+1)(n+1+2\delta _0)}{n+1-2\delta _0}(B_R)}\cdot \Vert \psi \Vert _{L^\infty (B_R)}+\Vert \nabla \psi \Vert _{L^\frac{(n+1)(n+1+2\delta _0)}{n+1-2\delta _0}(B_R)}\right) \nonumber \\&\quad \le C\left( R^{\frac{n+1-2\delta _0}{n+1+2\delta _0}+1}\cdot R^{2-\frac{n+1}{\frac{n+1}{2}+\delta _0}}\omega +\omega \right) \nonumber \\&\quad =C(R^2\omega +\omega )\nonumber \\&\quad \le CR^2\omega \nonumber \\&\quad =C\delta ^{25}\ll 1. \end{aligned}$$

Since we have $|{\tilde{u}}_0| \le c_0$, we apply Calderon–Zygmund to (3.25), for any $B_1(x)\subset B_R$ and $1<r<\infty$ and we get

3.31 $$\begin{aligned} \Vert {\tilde{u}}_0\Vert _{W^{2,r}\big (B_\frac{1}{2}(x)\big )}\le C_r. \end{aligned}$$

Hence by the Morrey embedding

$$\begin{aligned} \Vert \nabla {\tilde{u}}_0\Vert _{L^\infty (B_{R-1})}\le C. \end{aligned}$$

We define a modified discrepancy

3.32 $$\begin{aligned} \xi _G:=\frac{|\nabla {\tilde{u}}_0|^2}{2}-W({\tilde{u}}_0)-G({\tilde{u}}_0)-\varphi , \end{aligned}$$

for some function $G\in C^\infty ({\mathbb {R}})$ and $\varphi \in W^{2,2}(B_R)$ that we choose as in the following claims

### Claim

If we make the following choice of *G*,

3.33 $$\begin{aligned} G_\delta (r):=\delta \left( 1+\int _{-c_0-1}^r\exp \left( -\int _{-c_0-1}^t\frac{|W'(s)|+\delta }{2(W(s)+\delta )}ds\right) dt\right) \end{aligned}$$

then we have the properties

3.34 $$\begin{aligned} \begin{aligned} \delta&\le G_\delta ({\tilde{u}}_0)\le C\delta ,\\ 0&<G_\delta '({\tilde{u}}_0)\le \delta ,\\ 0&<-G_{\delta }''({\tilde{u}}_0)=G_\delta '({\tilde{u}}_0)\frac{|W'({\tilde{u}}_0)|+\delta }{2(W({\tilde{u}}_0)+\delta )}\le C. \end{aligned} \end{aligned}$$

Furthermore we have

3.35 $$\begin{aligned} G_\delta ' W'-2G''_\delta (W+G_\delta )&\ge \delta G'_\delta \end{aligned}$$

and

3.36 $$\begin{aligned} G_\delta '({\tilde{u}}_0)&\ge C\delta ^3. \end{aligned}$$

### Proof of Claim

The first three equations of (3.34) follow from the direct computations. For (3.35), since $G_\delta \ge \delta$, we obtain

$$\begin{aligned} G_\delta ' W'-2G''_\delta (W+G_\delta )&=G_\delta '\left( W'+\frac{|W'|+\delta }{(W+\delta )}(W+G_\delta )\right) \\&\ge G_\delta '\left( W'+\frac{|W'|+\delta }{(W+\delta )}(W+\delta )\right) \\&= G_\delta '\left( W'+|W'| + \delta \right) \\&\ge \delta G'_\delta . \end{aligned}$$

For (3.36), from the definition of $G_\delta$ (3.33) and the bound $|{\tilde{u}}_0|\le c_0+1$, we compute

$$\begin{aligned} \begin{aligned} G_\delta '({\tilde{u}}_0)&\ge \delta \exp \left( -\int _{-c_0-1}^{c_0+1}\frac{|W'(s)|+\delta }{2(W(s)+\delta )}ds\right) \\&\ge \delta \exp \left( -\int _{-c_0-1}^{-1}\left| \frac{d}{ds}\log (W(s)+\delta )\right| ds\right. \\&\quad \left. -\int _{-1}^0\left| \frac{d}{ds}\log (W(s)+\delta )\right| ds-(c_0+1)\right) \\&\ge \delta \exp \left( -\left( \log (W(-c_0-1)+\delta )-\log \delta \right) -\left( \log (1+\delta )-\log \delta \right) -(c_0+1)\right) \\&\ge \delta \exp \left( {\tilde{C}}-\log (\delta ^2)\right) \\&\ge C\delta ^3, \end{aligned} \end{aligned}$$

where we used *W* is an even function, increasing in $[-1,0]$ and decreasing in $[-c_0-1,-1]$. $\square$

### Claim

If we choose $\varphi$ to satisfy the Dirichlet problem

3.37 $$\begin{aligned} \begin{aligned} -\Delta \varphi&=|\langle \nabla {\tilde{u}}_0,\nabla {\tilde{f}}_0\rangle -(W'+G_\delta '){\tilde{f}}_0|>0 \quad \text { in }B_\frac{R}{2},\\ \varphi&=0 \quad \text { on }\partial B_\frac{R}{2} \end{aligned} \end{aligned}$$

then we have

3.38 $$\begin{aligned} \varphi \ge 0 \quad \text { in }B_\frac{R}{2} \end{aligned}$$

and

3.39 $$\begin{aligned} \Vert \varphi \Vert _{W^{1,\infty }(B_\frac{R}{2})}&\le CR^{4-\frac{n+1-2\delta _0}{n+1+2\delta _0}}\omega =C\delta ^{15+5\frac{n+1-2\delta _0}{n+1+2\delta _0}}. \end{aligned}$$

### Proof

Since we have $\varphi \ge 0$ in $\partial B_\frac{R}{2}$ by applying the maximum principle, we have $\varphi \ge 0$ in $B_\frac{R}{2}$ which gives us (3.38). The estimates (3.31), (3.29) and (3.30) bound the right hand side of (3.37), that is

$$\begin{aligned} \Vert \Delta \varphi \Vert _{L^\frac{(n+1)(n+1+2\delta _0)}{n+1-2\delta _0}(B_\frac{R}{2})}&=\left| \langle \nabla {\tilde{u}}_0,\nabla {\tilde{f}}_0\rangle -(W'+G_\delta '){\tilde{f}}_0\right| _{L^\frac{(n+1)(n+1+2\delta _0)}{n+1-2\delta _0}\big (B_\frac{R}{2}\big )}\\&\le CR^2\omega =C\delta ^{25}. \end{aligned}$$

Denote by $\varphi _R(x)=\varphi (\frac{Rx}{2})$, then the Calderon–Zygmund estimates give

$$\begin{aligned} \Vert \varphi \Vert _{W^{1,\infty }(B_\frac{R}{2})}&=\Vert \varphi _R\Vert _{W^{1,\infty }(B_1)}\le C\Vert \varphi _R\Vert _{W^{2,\frac{(n+1)(n+1+2\delta _0)}{n+1-2\delta _0}}(B_1)}\\&\le C\Vert \Delta \varphi _R\Vert _{L^\frac{(n+1)(n+1+2\delta _0)}{n+1-2\delta _0}(B_1)}\\&\le CR^{2-\frac{n+1-2\delta _0}{n+1+2\delta _0}}\Vert \Delta \varphi \Vert _{L^\frac{(n+1)(n+1+2\delta _0)}{n+1-2\delta _0}\big (B_\frac{R}{2}\big )}\\&\le CR^{4-\frac{n+1-2\delta _0}{n+1+2\delta _0}}\omega \\&=C\delta ^{15+5\frac{n+1-2\delta _0}{n+1+2\delta _0}} \end{aligned}$$

and hence we obtain (3.39). $\square$

We choose $\varphi$ according to (3.37). Notice if $\xi _G> 0$, then we have $\nabla {\tilde{u}}_0\ne 0$ and

3.40 $$\begin{aligned} W({\tilde{u}}_0)\le \frac{1}{2}|\nabla {\tilde{u}}_0|^2. \end{aligned}$$

The case $\xi _G\le 0$ immediately gives us our desired estimate since we are seeking an upper bound.

### Claim

For the choice of *G* as in (3.33) and $\varphi$ as in (3.37) we have the differential inequality

3.41 $$\begin{aligned} \Delta \xi _G&\ge -C\left( 1+\frac{\delta }{|\nabla {\tilde{u}}_0|}\right) \left( |\nabla \xi _G|+R^{4-\frac{n+1-2\delta _0}{n+1+2\delta _0}}\omega \right) +C(\delta ^6+\delta ^4) \end{aligned}$$

in $$B_\frac{R}{2}\cap \{\xi _G>0\}\cap \{\nabla {\tilde{u}}_0\ne 0\}$$.

### Proof

We compute the Laplacian of the modified discrepancy

3.42 $$\begin{aligned} \begin{aligned} \Delta \xi _G&=|\nabla ^2{\tilde{u}}_0|^2+\langle \nabla {\tilde{u}}_0,\nabla \Delta {\tilde{u}}_0\rangle -\Delta \varphi -(W'+G')\Delta {\tilde{u}}_0-(W'^\prime +G'^\prime )|\nabla {\tilde{u}}_0|^2\\&=|\nabla ^2{\tilde{u}}_0|^2+\langle \nabla {\tilde{u}}_0,W'^\prime \nabla {\tilde{u}}_0-\nabla {\tilde{f}}_0\rangle -\Delta \varphi -(W'+G')(W'({\tilde{u}}_0)-{\tilde{f}}_0)\\&\quad -(W'^\prime +G'^\prime )|\nabla {\tilde{u}}_0|^2\\&=|\nabla ^2{\tilde{u}}_0|^2-\langle \nabla {\tilde{u}}_0,\nabla {\tilde{f}}_0\rangle -\Delta \varphi -(W'+G')(W'({\tilde{u}}_0)-{\tilde{f}}_0)-G'^\prime |\nabla {\tilde{u}}_0|^2. \end{aligned} \end{aligned}$$

By differentiating (3.32), we have

$$\begin{aligned} \nabla \xi _G=\nabla ^2{\tilde{u}}_0\nabla {\tilde{u}}_0-(W'+G')\nabla {\tilde{u}}_0-\nabla \varphi , \end{aligned}$$

and thus

$$\begin{aligned} |\nabla ^2{\tilde{u}}_0|^2|\nabla {\tilde{u}}_0|^2&\ge |\nabla ^2{\tilde{u}}_0\nabla {\tilde{u}}_0|^2\\&\ge |\nabla \xi _G+(W'+G')\nabla {\tilde{u}}_0+\nabla \varphi |^2\\&\ge 2(W'+G')\left\langle \nabla {\tilde{u}}_0,\nabla (\xi _G+\varphi )\right\rangle +(W'+G')^2|\nabla {\tilde{u}}_0|^2. \end{aligned}$$

Dividing by $|\nabla {\tilde{u}}_0|^2$, the first term in (3.42), $|\nabla ^2{\tilde{u}}_0|^2$ , is bounded as follows

$$\begin{aligned} |\nabla ^2{\tilde{u}}_0|^2\ge \frac{2(W'+G')}{|\nabla {\tilde{u}}_0|^2}\langle \nabla {\tilde{u}}_0,\nabla (\xi _G+\varphi )\rangle +(W'+G')^2. \end{aligned}$$

The last term in (3.42) is

$$\begin{aligned} |\nabla {\tilde{u}}_0|^2=2(\xi _G+W+G+\varphi ). \end{aligned}$$

Substituting these into (3.42) and rearranging, we have in $B_R\subset \{\nabla {\tilde{u}}_0=0\}$

$$\begin{aligned}&\Delta \xi _G-\frac{2(W'+G')}{|\nabla {\tilde{u}}_0|^2}\langle \nabla {\tilde{u}}_0,\nabla \xi _G\rangle +2G'^\prime \xi _G\\&\quad \ge (W'+G')^2-W'(W'+G')-2G'^\prime (W+G)+\frac{2(W'+G')}{|\nabla {\tilde{u}}_0|^2}\langle \nabla {\tilde{u}}_0,\nabla \varphi \rangle \\&\qquad -2G'^\prime \varphi -\Delta \varphi -\langle \nabla {\tilde{u}}_0,\nabla {\tilde{f}}_0\rangle +(W'+G'){\tilde{f}}_0\\&\quad =(G')^2+\left( G'W'-2G'^\prime (W+G)\right) +\frac{2(W'+G')}{|\nabla {\tilde{u}}_0|^2}\langle \nabla {\tilde{u}}_0,\nabla \varphi \rangle -2G'^\prime \varphi -\Delta \varphi \\&\qquad -\langle \nabla {\tilde{u}}_0,\nabla {\tilde{f}}_0\rangle +(W'+G'){\tilde{f}}_0. \end{aligned}$$

We choose *G* to be (3.33) which allows us to apply the estimates (3.34) and (3.35) so that $\xi _G$ satisfies

3.43 $$\begin{aligned} \Delta \xi _G&\ge \frac{2(W'+G')}{|\nabla {\tilde{u}}_0|^2}\left\langle \nabla {\tilde{u}}_0,(\nabla \xi _G+\nabla \varphi )\right\rangle -2G_{\delta }''\xi _G\nonumber \\&\quad +(G_\delta ')^2+\delta G_\delta '-2G_\delta ''\varphi -\Delta \varphi -\langle \nabla {\tilde{u}}_0,\nabla {\tilde{f}}_0\rangle +(W'+G_\delta '){\tilde{f}}_0, \end{aligned}$$

in $B_R\cap \{\nabla {\tilde{u}}_0\ne 0\}$. Furthermore we have by (3.40)

$$\begin{aligned} |W'({\tilde{u}}_0)|^2 =|{\tilde{u}}_0|^2(1-|{\tilde{u}}_0|^2)^2 \le C W({\tilde{u}}_0) \le C |\nabla {\tilde{u}}_0|^2. \end{aligned}$$

From (3.34), the bounds on $G_\delta$ and its derivatives, we get

3.44 $$\begin{aligned} \frac{|(W'+G_\delta ')({\tilde{u}}_0)\nabla {\tilde{u}}_0|}{|\nabla {\tilde{u}}_0|^2}\le \frac{\frac{1}{2}|\nabla {\tilde{u}}_0|^3+\delta |\nabla {\tilde{u}}_0|}{|\nabla {\tilde{u}}_0|^2}\le C\left( 1+\frac{\delta }{|\nabla {\tilde{u}}_0|}\right) . \end{aligned}$$

Substituting in (3.37), (3.39), and (3.44) into (3.43) and using the fact that $G''<0$, we have

$$\begin{aligned} \Delta \xi _G&\ge -C\left( 1+\frac{\delta }{|\nabla {\tilde{u}}_0|}\right) (|\nabla \xi _G|+|\nabla \varphi |)+(G_\delta ')^2+\delta G_\delta '-\Delta \varphi +\Delta \varphi \\&\ge -C\left( 1+\frac{\delta }{|\nabla {\tilde{u}}_0|}\right) \left( |\nabla \xi _G|+R^{4-\frac{n+1-2\delta _0}{n+1+2\delta _0}}\omega \right) +(G_\delta ')^2+\delta G_\delta '. \end{aligned}$$

Thus applying Eq. (3.36) in $B_\frac{R}{2}\cap \{\xi _G>0\}\cap \{\nabla {\tilde{u}}_0\ne 0\}$, we have (3.41)

3.45 $$\begin{aligned} \Delta \xi _G&\ge -C\left( 1+\frac{\delta }{|\nabla {\tilde{u}}_0|}\right) (|\nabla \xi _G|+R^{4-\frac{n+1-2\delta _0}{n+1+2\delta _0}}\omega )+C(\delta ^6+\delta ^4). \end{aligned}$$

$$\square $$

We define

3.46 $$\begin{aligned} \eta :=\sup _{B_1}\xi _G \end{aligned}$$

and consider two cases : **case i)** $\eta :=\sup _{B_1}\xi _G< \delta$. Since

$$\begin{aligned} \xi _G:=\frac{|\nabla {\tilde{u}}_0|^2}{2}-W({\tilde{u}}_0)-G({\tilde{u}}_0)-\varphi <\delta , \end{aligned}$$

by (3.34) and (3.39) this implies

$$\begin{aligned} \frac{|\nabla {\tilde{u}}_0|^2}{2}-W({\tilde{u}}_0)\le \delta +G({\tilde{u}}_0)+\varphi \le \delta + C\delta + CR^{4-\frac{n+1-2\delta _0}{n+1+2\delta _0}}\omega . \end{aligned}$$

Our choices give $CR^{4-\frac{n+1-2\delta _0}{n+1+2\delta _0}}\omega =C\delta ^{15+5\frac{n+1-2\delta _0}{n+1+2\delta _0}} \le C\delta$ so

$$\begin{aligned} \frac{|\nabla {\tilde{u}}_0|^2}{2}-W({\tilde{u}}_0)\le C\delta \end{aligned}$$

which, after integrating proves (3.26).

**case ii)** $\eta :=\sup _{B_1}\xi _G\ge \delta > 0$. We choose a cutoff function $\lambda \in C^2_0(B_\frac{R}{2})$ satisfying $0\le \lambda \le 1$, $\lambda \equiv 1$ on $B_\frac{R}{4}$ and $|\nabla ^j\lambda |\le CR^{-j}$ for $j=1,2$. Then $\exists x_0\in B_\frac{R}{2}$ such that

$$\begin{aligned} (\lambda \xi _G)(x_0)=\max \left\{ (\lambda \xi _G)(x): x\in {\bar{B}}_\frac{R}{2}\right\} \ge \eta >0. \end{aligned}$$

By (3.31) we have $\xi _G\le C$ for some $C(c_0,\Lambda _0, E_0,n)>0$ in $B_{R-1}$, and thus

$$\begin{aligned} \lambda (x_0)\ge \frac{\eta }{C}. \end{aligned}$$

Moreover,

$$\begin{aligned} |\nabla {\tilde{u}}_0(x_0)|^2\ge 2\xi _G(x_0)\ge 2(\lambda \xi _G)(x_0)\ge 2\eta \ge 2\delta >0. \end{aligned}$$

Since $x_0$ is a critical point, $\nabla (\lambda \xi _G)(x_0)=0$, and we get

$$\begin{aligned} |\nabla \xi _G(x_0)|=\lambda (x_0)^{-1}|\nabla \lambda (x_0)|\xi _G(x_0)\le C(R\eta )^{-1}. \end{aligned}$$

At a maximum point $x_0$, the Laplacian of the function $\lambda \xi _G$ satisfies

$$\begin{aligned} 0&\ge \Delta (\lambda \xi _G)(x_0)\\&=\lambda (x_0)\Delta \xi _G(x_0)+2\langle \nabla \lambda (x_0),\nabla \xi _G(x_0)\rangle +\Delta \lambda (x_0)\xi _G(x_0), \end{aligned}$$

and thus

3.47 $$\begin{aligned} \Delta \xi _G(x_0)&\le \lambda (x_0)^{-1}\left( C|\nabla \lambda (x_0)||\nabla \xi _G(x_0)|+|\Delta \lambda (x_0)||\xi _G(x_0)|\right) \nonumber \\&\le C\eta ^{-1}\left( CR^{-1}(R\eta )^{-1}+CR^{-2}\right) \nonumber \\&\le CR^{-2}\eta ^{-1}(1+\eta ^{-1})\nonumber \\ {}&\le CR^{-2}\eta ^{-1}(1+\delta ^{-1})\nonumber \\ {}&\le CR^{-2}\eta ^{-1}\delta ^{-1}, \end{aligned}$$

since $\delta \ll 1$. Combining (3.41) and (3.47) we have

$$\begin{aligned} CR^{-2}\eta ^{-1}\delta ^{-1}&\ge -C\left( 1+\frac{\delta }{|\nabla {\tilde{u}}_0(x_0)|}\right) \left( |\nabla \xi _G|+R^{4-\frac{n+1-2\delta _0}{n+1+2\delta _0}}\omega \right) +C(\delta ^6+\delta ^4)\\&\ge C\left[ \left( 1+\frac{\delta }{2\delta }\right) \left( (R\eta )^{-1}+R^{4-\frac{n+1-2\delta _0}{n+1+2\delta _0}}\omega \right) +\delta ^4\right] . \end{aligned}$$

Thus the last term above is bounded by

$$\begin{aligned} \delta ^4\le C\left( R^{-2}\eta ^{-1}\delta ^{-1}+(R\eta )^{-1}\right) +CR^{4-\frac{n+1-2\delta _0}{n+1+2\delta _0}}\omega . \end{aligned}$$

By our choice of $p_1=2, p_2=15$, we have $R^{4-\frac{n+1-2\delta _0}{n+1+2\delta _0}}\omega =R^{15+5\frac{n+1-2\delta _0}{n+1+2\delta _0}}\ll \delta ^4$. So

$$\begin{aligned} \delta ^4&\le C\left( R^{-2}\eta ^{-1}\delta ^{-1}+(R\eta )^{-1}\right) , \end{aligned}$$

dividing both sides by $\delta ^4\eta ^{-1}$ gives

$$\begin{aligned} \eta&\le C\left( R^{-2}\delta ^{-4}\delta ^{-1}+R^{-1}\delta ^{-4}\right) \\&\le C\delta . \end{aligned}$$

Namely, assuming (3.46) or not, we have

$$\begin{aligned} \xi _G\le C\delta , \end{aligned}$$

and thus by (3.39)

$$\begin{aligned} \frac{|\nabla {\tilde{u}}_0|^2}{2}-W({\tilde{u}}_0)&=\xi _G+G_\delta ({\tilde{u}}_0)+\varphi \\&\le C\delta +R^{4-\frac{n+1-2\delta _0}{n+1+2\delta _0}}\omega \\&\le C\delta +\delta ^{15+5\frac{n+1-2\delta _0}{n+1+2\delta _0}}\\&\le C\delta . \end{aligned}$$

This proves (3.26) and as a consequence (3.19). If $|{\tilde{u}}|\ge 1-\tau$ in $B_\frac{1}{2}$, then (3.20) follows because the left hand side is less than the second term on the right. So we only need to consider the case there exists $x_0\in B_\frac{1}{2}$ with ${\tilde{u}}(x_0)\le 1-\tau$. By the Sobolev inequality and Calderon–Zygmund estimates we bound ${\tilde{u}}$ in the Hölder norm as follows

$$\begin{aligned} \Vert {\tilde{u}}\Vert _{C^{0,\frac{2\delta _0}{(n+1)+\delta _0}}(B_1)}&\le \Vert {\tilde{u}}\Vert _{W^{2,\frac{n+1}{2}+\delta _0}(B_1)}\\&\le {\tilde{C}}\left( \Vert W'({\tilde{u}})\Vert _{L^{\frac{n+1}{2}+\delta _0}(B_1)}+\Vert {\tilde{f}}\Vert _{L^{\frac{n+1}{2}+\delta _0}(B_1)}+\Vert {\tilde{u}}\Vert _{L^{\frac{n+1}{2}+\delta _0}(B_1)}\right) \\&\le C. \end{aligned}$$

Therefore $|{\tilde{u}}|\le 1-\frac{\tau }{2}$ and $W({\tilde{u}})\ge \frac{\tau ^2}{4}$ in $B_{\left( \frac{\tau }{2C}\right) ^\frac{(n+1)+\delta _0}{2\delta _0}}\subset B_1$. So

$$\begin{aligned} \int _{B_\frac{1}{2}}W({\tilde{u}})\ge \frac{\tau ^2}{4}\left( \frac{\tau }{2{\tilde{C}}_2}\right) ^\frac{(n+1)[(n+1)+\delta _0]}{2\delta _0}=C\tau ^\frac{(n+1)^2+(n+1)\delta _0+4\delta _0}{2\delta _0}. \end{aligned}$$

By our choice $p_3=\frac{2\delta _0}{(n+1)^2+(n+1)\delta _0+6\delta _0}$,

$$\begin{aligned} \int _{B_\frac{1}{2}}\left( \frac{|\nabla {\tilde{u}}|^2}{2}-W({\tilde{u}})\right) _+&\le C\delta \\&\le C\tau ^\frac{(n+1)^2+(n+1)\delta _0+6\delta _0}{2\delta _0}\\&\le C\tau \tau ^\frac{(n+1)^2+(n+1)\delta _0+4\delta _0}{2\delta _0}\\&\le C\tau \int _{B_\frac{1}{2}}\left( \frac{|\nabla {\tilde{u}}|^2}{2}+W({\tilde{u}})\right) , \end{aligned}$$

which proves (3.20). $$\square $$

Next we derive energy estimates away from transition regions.

### Proposition 3.5

([8, Proposition 3.4]) For any $n\ge 2$ , $0\le \delta \le \delta _1$, $\varepsilon > 0, u_\varepsilon \in C^2(\Omega ), f_\varepsilon \in C^0(\Omega )$, if

$$\begin{aligned} -\varepsilon \Delta u_\varepsilon + \frac{W'(u_\varepsilon )}{\varepsilon } = f_\varepsilon \quad \text { in } \Omega \end{aligned}$$

and

$$\begin{aligned} \Omega '\subset \subset \Omega , 0 < r \le d(\Omega ',\partial \Omega ) \end{aligned}$$

then

$$\begin{aligned}&\int _{\{|u_\varepsilon |\ge 1 - \delta \} \cap \Omega ' }\left( \varepsilon |\nabla u_\varepsilon |^2 + \frac{W(u_\varepsilon )}{\varepsilon } + \frac{W'(u_\varepsilon )^2}{\varepsilon } \right) \\&\quad \le C\delta \int _{\{| u_\varepsilon | \le 1 -\delta \} \cap \Omega } \varepsilon |\nabla u_\varepsilon |^2 + C \varepsilon \int _\Omega |f_\varepsilon |^2 + C\left( \frac{\delta }{r} + \frac{\delta ^2}{r^2} \right) \varepsilon {\mathcal {L}}^{n+1}(\Omega )\\&\qquad +\frac{C\varepsilon }{r^2} \int _{\{|u_\varepsilon |\ge 1\} \cap \Omega }W'(u_\varepsilon )^2. \end{aligned}$$

(Notice the power of $f_\varepsilon$ in the above inequality will still be 2 instead of $\frac{n+1}{2}+\delta _0$.)

### Proof

Define a continuous function

$$\begin{aligned} g(t)= {\left\{ \begin{array}{ll} W'(t),&{}\text { for }|t|\ge 1-\delta \\ 0,&{}\text { for }|t|\le t_0\\ \text { linear,}&{}\text { for }t\in [-1+\delta ,-t_0]\cup [t_0,1-\delta ], \end{array}\right. } \end{aligned}$$

where $t_0=\frac{1}{\sqrt{3}}$ is chosen to be the number in (0, 1) such that $W''(t_0)=0$. Clearly $|g|\le |W'|$. For $\eta \in C_0^1(\Omega )$ satisfying $0\le \eta \le 1$, $\eta \equiv 1$ on $\Omega '$ and $|\nabla \eta |\le Cr^{-1}$, we get by integration by parts

3.48 $$\begin{aligned} \begin{aligned} \int _{\Omega } f_\varepsilon g(u_\varepsilon )\eta ^2&=\int _{\Omega }\left( -\varepsilon \Delta u_\varepsilon +\frac{W'(u_\varepsilon )}{\varepsilon }\right) g(u_\varepsilon )\eta ^2\\&=\int _{\Omega }\varepsilon g'(u_\varepsilon )|\nabla u_\varepsilon |^2\eta ^2+2\int _{\Omega }\varepsilon g(u_\varepsilon )\eta \langle \nabla u_\varepsilon ,\nabla \eta \rangle \\&\quad +\int _{\Omega }\frac{W'(u_\varepsilon )}{\varepsilon }g(u_\varepsilon )\eta ^2. \end{aligned}\nonumber \\ \end{aligned}$$

The left hand side of (3.48) can be bounded by

3.49 $$\begin{aligned} \int _\Omega f_\varepsilon g(u_\varepsilon )\eta ^2&\le \frac{\varepsilon }{2}\int _\Omega |f_\varepsilon |^2+\frac{1}{2\varepsilon }\int _\Omega g(u_\varepsilon )^2\eta ^2\le \frac{\varepsilon }{2}\int _\Omega |f_\varepsilon |^2\nonumber \\&\quad +\frac{1}{2\varepsilon }\int _\Omega W'(u_\varepsilon )g(u_\varepsilon )\eta ^2. \end{aligned}$$

By the definition of *g* above, we have

$$\begin{aligned} |g(t)|&\le |g(1-\delta )|=W'(1-\delta )\le C\delta ,\\ |g'(t)|&\le \frac{|g(1-\delta )|}{1-\delta }\le \frac{|g(1-\delta )|}{1-\delta _1}\le C\delta , \end{aligned}$$

for $|t|\le 1-\delta$. Applying these estimates to the second term on the right hand side of (3.48) we get the bound

3.50 $$\begin{aligned}&\left| 2\int _{\Omega }\varepsilon g(u_\varepsilon )\eta \langle \nabla u_\varepsilon ,\nabla \eta \rangle \right| \nonumber \\&\quad \le 2\delta \int _{\{|u_\varepsilon |\le 1-\delta \}}\varepsilon \eta |\nabla u_\varepsilon ||\nabla \eta |+\left| \int _{\{|u_\varepsilon |\ge 1-\delta \}}\varepsilon W'(u_\varepsilon )\langle \nabla u_\varepsilon ,\nabla \eta \rangle \right| \nonumber \\&\quad \le C\delta \int _{\{|u_\varepsilon |\le 1-\delta \}}\varepsilon |\nabla u_\varepsilon |^2+\varepsilon \delta r^{-1}{\mathcal {L}}^{n+1}(\Omega )\nonumber \\&\qquad +\tau \int _{\{|u_\varepsilon |\ge 1-\delta \}}\varepsilon |\nabla u_\varepsilon |^2\eta ^2+C\varepsilon \tau ^{-1} r^{-2}\int _{\{|u_\varepsilon |\ge 1-\delta \}}W'(u_\varepsilon )^2, \end{aligned}$$

for $\tau >0$. As $g'(t)=W''(t)\ge C_W>0$ for $|t|\ge 1-\delta$, we obtain from (3.48), (3.49) and (3.50)

$$\begin{aligned}&C_W\int _{\{|u_\varepsilon |\ge 1-\delta \}}\varepsilon |\nabla u_\varepsilon |^2+\frac{1}{2\varepsilon }\int _\Omega W'(u_\varepsilon )g(u_\varepsilon )\eta ^2\\&\quad \le C_W\delta \int _{\{|u_\varepsilon |\le 1-\delta \}}\varepsilon |\nabla u_\varepsilon |^2+\tau \int _{\{|u_\varepsilon |\ge 1-\delta \}}\varepsilon |\nabla u_\varepsilon |^2\eta ^2+\frac{\varepsilon }{2}\int _\Omega |f_\varepsilon |^2\\&\qquad +\left( \delta r^{-1}+C\delta ^2\tau ^{-1}r^{-2}\right) {\mathcal {L}}^{n+1}(\Omega )+C\varepsilon \tau ^{-1}r^{-2}\int _{\{|u_\varepsilon |\ge 1\}}W'(u_\varepsilon )^2. \end{aligned}$$

Choosing $\tau =\frac{C_W}{2}$, and using $W(t)\le C_WW'(t)^2$ for $|t|\ge 1-\delta$ we get

$$\begin{aligned}&\int _{\{|u_\varepsilon |\ge 1-\delta \}\cap \Omega '}\left( \varepsilon |\nabla u_\varepsilon |^2+\frac{W(u_\varepsilon )}{\varepsilon }+\frac{W'(u_\varepsilon )^2}{\varepsilon }\right) \\&\quad \le C\int _{\{|u_\varepsilon |\ge 1-\delta \}\cap \Omega '}\left( \varepsilon |\nabla u_\varepsilon |^2+\frac{W'(u_\varepsilon )^2}{\varepsilon }\right) \\&\quad \le C\delta \int _{\{|u_\varepsilon |\le 1-\delta \}}\varepsilon |\nabla u_\varepsilon |^2+C\varepsilon \int _\Omega |f_\varepsilon |^2+C\varepsilon \left( \delta r^{-1}+\delta ^2r^{-2}\right) {\mathcal {L}}^{n+1}(\Omega )\\&\qquad +C\varepsilon r^{-2}\int _{\{|u_\varepsilon |\ge 1\}}W'(u_\varepsilon )^2, \end{aligned}$$

which completes the proof. $\square$

The following proposition shows for all $\varepsilon$ sufficiently small, if $u_\varepsilon$ satisfies the inhomogeneous Allen–Cahn equation then we can control the last term $\int _{\{|u_\varepsilon |\ge 1\} \cap \Omega '} W'(u_\varepsilon )^2$ in Proposition 3.5 by applying the proposition inductively.

### Proposition 3.6

([8, Proposition 3.5]) For $n\ge 2, \varepsilon > 0, u_\varepsilon \in C^2(\Omega ), f_\varepsilon \in C^0(\Omega )$, if

$$\begin{aligned} -\varepsilon \Delta u_\varepsilon + \frac{W'(u_\varepsilon )}{\varepsilon } = f_\varepsilon \quad \text { in } \Omega \end{aligned}$$

and $$\Omega '\subset \subset \Omega , 0 < r \le d(\Omega ',\partial \Omega )$$ then

$$\begin{aligned} \int _{\{|u_\varepsilon |\ge 1\} \cap \Omega '} W'(u_\varepsilon )^2&\le C_k( 1 + r^{-2k}\varepsilon ^{2k}) \varepsilon ^2 \int _{\Omega '_{i-1}} |f_\varepsilon |^2 \\ {}&\quad + C_k r^{-2k} \varepsilon ^{2k} \int _{\{|u_\varepsilon | \ge 1\} \cap \Omega } W'(u_\varepsilon )^2 \end{aligned}$$

for all $$ k \in {\mathbb {N}}_0$$.

### Proof

For any $k\in {\mathbb {N}}^+$ we choose a sequence of open sets

$$\begin{aligned} \Omega _i':={\left\{ \begin{array}{ll} \Omega &{}\text { for }i=0\\ \left\{ x\in \Omega |d(x,\Omega ')<\frac{(k-i)r}{k}\right\} &{}\text { for } i=1,...,k-1,\\ \Omega '&{}\text { for } i=k. \end{array}\right. } \end{aligned}$$

This sequence satisfies

$$\begin{aligned} \Omega '=\Omega _k'\subset \subset \Omega _{k-1}'\subset \subset ...\subset \subset \Omega _0'=\Omega , \end{aligned}$$

with $d(\Omega _i',\Omega _{i-1}')\ge \frac{r}{k}$ for $i=1,...,k$. Applying Proposition (3.5) with $\delta =0$, we have

$$\begin{aligned} \int _{\{|u_\varepsilon |\ge 1\}\cap \Omega _i'}W'(u_\varepsilon )^2\le C\varepsilon ^2\int _{\Omega '_{i-1}} |f_\varepsilon |^2+Ck^2r^{-2}\varepsilon ^2\int _{\{|u_\varepsilon |\ge 1\}\cap \Omega _{i-1}'}W'(u_\varepsilon )^2, \end{aligned}$$

for $i=1,...,k$. The conclusion is obtained by applying the above inequality inductively *k* times. $\square$

We conclude with the following integral bound for positive part of discrepancy measure.

### Lemma 3.7

([8, Lemma 3.1] for all *n*) Let $n\ge 2$, $0<\delta \le \delta _1$ (where $\delta _1$ given as in Lemma 3.4), $0<\varepsilon \le \rho$, $\rho _0:=\max \{2,1+\delta ^{-M}\varepsilon \}\rho$ for some large universal constant *M*. If $u_\varepsilon \in C^2(B_{\rho _0}), f_\varepsilon \in C^0(B_{\rho _0})$ satisfies (1.1) in $B_{\rho _0}(0)$ then the positive part of the discrepancy measure satisfies

$$\begin{aligned}&\rho ^{-n}\int _{B_\rho }\left( \frac{\varepsilon |\nabla u_\varepsilon |^2}{2}-\frac{W(u_\varepsilon )}{\varepsilon }\right) _+\\&\quad \le C\delta ^{p_3}\rho ^{-n}\int _{B_{2\rho }}\left( \frac{\varepsilon |\nabla u_\varepsilon |^2}{2}+\frac{W(u_\varepsilon )}{\varepsilon }\right) +C\delta ^{-M}\varepsilon \rho ^{-n}\int _{B_{\rho _0}}|f_\varepsilon |^2\\&\qquad +C\delta ^{-M}\rho ^{-n}\int _{B_{\rho _0}\cap \{|u_\varepsilon |\ge 1\}}\frac{W'(u_\varepsilon )^2}{\varepsilon }+C\left( \frac{\varepsilon }{\rho }\right) \delta . \end{aligned}$$

### Proof

We prove the case $0<\varepsilon \le \rho =1$. The case for other $\rho >0$ follows by rescaling to $\rho =1$. For $0<\delta \le \delta _1$ we choose $R(\delta )=\frac{1}{\delta ^{p_1}}$ and $\omega (\delta )=C_\omega \delta ^{p_2}$ as in Lemma 3.4. Let $\{x_i\}_{i\in {\textbf{I}}}\subset B_1, {\textbf{I}}\subset {\mathbb {N}}$ be a maximal collection of points satisfying

$$\begin{aligned} \min _{i\ne j}|x_i-x_j|\ge \frac{\varepsilon }{2}. \end{aligned}$$

Since $\varepsilon \le 1$, we have

$$\begin{aligned}&B_1(0)\subset \cup _{i\in {\textbf{I}}}\bar{B}_{\frac{\varepsilon }{2}}(x_i)\subset B_{\frac{3}{2}}(0),\\&\sum _{i\in {\textbf{I}}}\chi _{B_\varepsilon (x_i)}\le C_n\chi _{B_2(0)},\\&\sum _{i\in {\textbf{I}}}\chi _{B_{2R\varepsilon }(x_i)}\le C_nR^{n+1}\chi _{B_{1+2R\varepsilon }(0)}. \end{aligned}$$

For $i\in {\textbf{I}}$ and $x\in B_{2R}$, we define the rescaled and translated functions as

$$\begin{aligned} {\tilde{u}}_i(x)&:=u_\varepsilon (x_i+\varepsilon x),\\ {\tilde{f}}_i(x)&:=\varepsilon f_\varepsilon (x_i+\varepsilon x), \end{aligned}$$

which satisfy the rescaled equation

3.51 $$\begin{aligned} -\Delta {\tilde{u}}_i+W'({\tilde{u}}_i)={\tilde{f}}_i,\quad \text { in } B_{2R}(0). \end{aligned}$$

For ${\tilde{u}}_i, {\tilde{f}}_i$ to be well-defined, we choose $M\ge 5n+6$ and $\delta _1\le \frac{1}{2}$ so that

$$\begin{aligned} x_i+\varepsilon x\in B_{1+2R\varepsilon }(0)\subset B_{1+\delta ^{-M}\varepsilon }(0)\subset B_{\rho _0}(0). \end{aligned}$$

We decompose the index set ${\textbf{I}}$ into

$$\begin{aligned} {\textbf{I}}_1&:=\left\{ i\in {\textbf{I}}:\Vert f_\varepsilon \Vert _{L^{\frac{n+1}{2}+\delta _0}(B_{2R\varepsilon }(x_i))}<\varepsilon ^{\frac{n+1}{2}-1+\delta _0}\omega , \Vert (|u_\varepsilon |-1)_+\Vert _{L^1(B_{2R\varepsilon }(x_i))}<C_\omega \varepsilon ^{n+1}\right\} ,\\ {\textbf{I}}_2&:={\textbf{I}}\setminus {\textbf{I}}_1. \end{aligned}$$

For $i\in {\textbf{I}}_1$, we have

$$\begin{aligned}&\Vert {\tilde{f}}_i\Vert _{L^{\frac{n+1}{2}+\delta _0}(B_{2R}(0))}=\varepsilon ^{-\frac{n+1}{2}-\delta _0}\Vert \varepsilon f_\varepsilon \Vert _{L^{\frac{n+1}{2}+\delta _0}(B_{2R\varepsilon }(x_i))}<\omega \le C_\omega ,\\&\Vert (|{\tilde{u}}_i|-1)_+\Vert _{L^1(B_{2R}(x_i))}=\varepsilon ^{-n-1}\Vert (| u_\varepsilon |-1)_+\Vert _{L^1(B_{2R\varepsilon }(x_i))}<C_\omega . \end{aligned}$$

By the condition $\Vert u\Vert _{L^\infty }\le c_0$ in the condition of Theorem 1.1, and choosing $C_\omega$ sufficiently small, we have

$$\begin{aligned} \Vert {\tilde{u}}_i\Vert _{L^\infty (B_R)}\le 1+C\cdot C_\omega \le 2. \end{aligned}$$

Applying Lemma 3.4 to ${\tilde{u}}_i$ gives (with $p_3$ from Lemma 3.4)

$$\begin{aligned} \int _{B_\frac{1}{2}}\left( \frac{|\nabla {\tilde{u}}_i|^2}{2}-W({\tilde{u}}_i)\right) _+&\le C{\delta ^{p_3}}\int _{B_\frac{1}{2}}\left( \frac{|\nabla {\tilde{u}}_i|^2}{2}+W({\tilde{u}}_i)\right) \\&\quad +\int _{B_\frac{1}{2}\cap \{|{\tilde{u}}_i|\ge 1-\delta \}}\frac{|\nabla {\tilde{u}}_i|^2}{2}. \end{aligned}$$

Rescaling back, we get

$$\begin{aligned} \int _{B_\frac{\varepsilon }{2}(x_i)}\left( \frac{\varepsilon |\nabla u_\varepsilon |^2}{2}-\frac{W( u_\varepsilon )}{\varepsilon }\right) _+&\le C{\delta ^{p_3}}\int _{B_\frac{\varepsilon }{2}(x_i)}\left( \frac{\varepsilon |\nabla u_\varepsilon |^2}{2}+\frac{W( u_\varepsilon )}{\varepsilon }\right) \\ {}&\quad +\int _{B_\frac{\varepsilon }{2}(x_i)\cap \{| u_\varepsilon |\ge 1-\delta \}}\frac{\varepsilon |\nabla u_\varepsilon |^2}{2}. \end{aligned}$$

Summing over $i\in {\textbf{I}}_1$ and noticing $B_{\frac{\varepsilon }{2}}(x_i)$ are disjoint, we get

3.52 $$\begin{aligned} \sum _{i\in {\textbf{I}}_1}\int _{B_\frac{\varepsilon }{2}(x_i)}\left( \frac{\varepsilon |\nabla u_\varepsilon |^2}{2}-\frac{W( u_\varepsilon )}{\varepsilon }\right) _+&\le C\delta ^{p_3}\int _{B_\frac{3}{2}(0)}\left( \frac{\varepsilon |\nabla u_\varepsilon |^2}{2}+\frac{W( u_\varepsilon )}{\varepsilon }\right) \nonumber \\&\quad +C\int _{B_\frac{3}{2}(0)\cap \{|u_\varepsilon |\ge 1-\delta \}}\frac{\varepsilon |\nabla u_\varepsilon |^2}{2}\nonumber \\&\le C\delta ^{p_3}\int _{B_2(0)}\left( \frac{\varepsilon |\nabla u_\varepsilon |^2}{2}+\frac{W( u_\varepsilon )}{\varepsilon }\right) \nonumber \\&\quad +C\varepsilon \int _{B_2(0)}|f_\varepsilon |^2\nonumber \\&\quad +C\varepsilon \left( \delta +\int _{B_2(0)\cap \{|u_\varepsilon |\ge 1\}}W'(u_\varepsilon )^2\right) , \end{aligned}$$

where we used Proposition 3.5 in the last line. Since for $n\ge 3$ (the $n=2$ case requires $\delta _0\ge \frac{1}{2}$, but has already been addressed in [8])

$$\begin{aligned} W'(t)^2\ge 4t^2(1+t)^2(1-t)^2\ge C_Wt^2(|t|-1)^2\ge C_W(|t|-1)_+^{\frac{n+1}{2}+\delta _0}. \end{aligned}$$

Thus for $i\in {\textbf{I}}_2$ (at least one of the bounds in ${\textbf{I}}_1$ does not hold), we have

$$\begin{aligned} C_\omega&\le \int _{B_{2R}(0)}(|{\tilde{u}}_i|-1)_+^{\frac{n+1}{2}+\delta _0}+\omega ^{-2}\int _{B_{2R}}{\tilde{f}}_i^2\\&\le C\int _{B_{2R}(0)\cap \{|{\tilde{u}}_i|\ge 1\}}W'({\tilde{u}}_i)^2+\omega ^{-2}\int _{B_{2R}}{\tilde{f}}_i^2. \end{aligned}$$

By elliptic estimates applied to the rescaled Eq. (3.51), we get

$$\begin{aligned} \int _{B_{\frac{1}{2}}}|\nabla {\tilde{u}}_i|^2&\le {\tilde{C}}\int _{B_1}\left( W'({\tilde{u}}_i)^2+{\tilde{u}}_i^2+{\tilde{f}}_i^2\right) \\&\le {\tilde{C}}\int _{B_{2R}}\left( W'({\tilde{u}}_i)^2+{\tilde{f}}_i^2\right) +{\tilde{C}}\omega _n c_0^2C_\omega ^{-1}C_\omega \\&\le C\int _{B_{2R}}\left( W'({\tilde{u}}_i)^2+\omega ^{-2}{\tilde{f}}_i^2\right) , \end{aligned}$$

where we used $\Vert {\tilde{u}}_i\Vert _{L^\infty }\le c_0$. Rescaling back gives

$$\begin{aligned} \int _{B_\frac{\varepsilon }{2}(x_i)}\varepsilon |\nabla u_\varepsilon |^2\le C\int _{B_{2R\varepsilon }(x_i)}\left( \frac{W'(u_\varepsilon )^2}{\varepsilon }+\varepsilon \omega ^{-2}|f_\varepsilon |^2\right) . \end{aligned}$$

Then summing over $i\in {\textbf{I}}_2$ we get

3.53 $$\begin{aligned} \sum _{i\in {\textbf{I}}_2}\int _{B_\frac{\varepsilon }{2}(x_i)}\varepsilon |\nabla u_\varepsilon |^2&\le \sum _{i\in {\textbf{I}}_2}C\int _{B_{2R\varepsilon }(x_i)}\left( \frac{W'(u_\varepsilon )^2}{\varepsilon }+\varepsilon \omega ^{-2}|f_\varepsilon |^2\right) \nonumber \\&\le CR^{n+1}\int _{B_{1+2R\varepsilon }(0)}\left( \frac{W'(u_\varepsilon )^2}{\varepsilon }+\varepsilon \omega ^{-2}|f_\varepsilon |^2\right) \nonumber \\&\le C\delta ^{-M}\int _{B_{1+\delta ^{-M}\varepsilon }(0)}\left( \frac{W'(u_\varepsilon )^2}{\varepsilon }+\varepsilon |f_\varepsilon |^2\right) , \end{aligned}$$

for large enough *M* since both $R=\delta ^{-p_1}$ and $\omega =\delta ^{p_2}$ are fixed powers of $\delta$. Combining (3.52) and (3.53) we get

$$\begin{aligned}&\int _{B_1}\left( \frac{\varepsilon |\nabla u_\varepsilon |^2}{2}-\frac{W(u_\varepsilon )}{\varepsilon }\right) _+\\&\quad \le \sum _{i\in {\textbf{I}}_1}\int _{B_\frac{\varepsilon }{2}(x_i)}\left( \frac{\varepsilon |\nabla u_\varepsilon |^2}{2}-\frac{W( u_\varepsilon )}{\varepsilon }\right) _++\sum _{i\in {\textbf{I}}_2}\int _{B_\frac{\varepsilon }{2}(x_i)}\frac{\varepsilon |\nabla u_\varepsilon |^2}{2}\\&\quad \le C\delta ^{p_3}\int _{B_2(0)}\left( \frac{\varepsilon |\nabla u_\varepsilon |^2}{2}+\frac{W( u_\varepsilon )}{\varepsilon }\right) \\&\qquad +C\varepsilon \int _{B_2(0)}|f_\varepsilon |^2+C\varepsilon \left( \delta +\int _{B_2(0)\cap \{|u_\varepsilon |\ge 1\}}W'(u_\varepsilon )^2\right) \\&\qquad +C\delta ^{-M}\int _{B_{1+\delta ^{-M}\varepsilon }(0)}\left( \frac{W'(u_\varepsilon )^2}{\varepsilon }+\varepsilon |f_\varepsilon |^2\right) \\&\quad \le C\delta ^{p_3}\int _{B_2(0)}\left( \frac{\varepsilon |\nabla u_\varepsilon |^2}{2}+\frac{W( u_\varepsilon )}{\varepsilon }\right) +C\varepsilon \delta +C\varepsilon \delta ^{-M}\int _{B_{\max \{2,1+\delta ^{-M}\varepsilon \}}(0)}|f_\varepsilon |^2\\&\qquad +C\delta ^{-M}\int _{B_{\max \{2,1+\delta ^{-M}\varepsilon \}}(0)}\frac{W'(u_\varepsilon )^2}{\varepsilon }. \end{aligned}$$

This completes the proof for $\rho =1$ and rescaling gives the cases for other $\rho >0$. $\square$

As a result of these, we have the $L^1$ convergence of the positive part of the discrepancy measure as $\varepsilon \rightarrow 0$.

### Lemma 3.8

If we consider $\xi _\varepsilon =\xi _{\varepsilon ,+} - \xi _{\varepsilon ,-}$ the decomposition of $\xi _\varepsilon$ into positive and negative variations then

$$\begin{aligned} \xi _{\varepsilon ,+}\rightarrow 0 \quad \text { as }\varepsilon \rightarrow 0. \end{aligned}$$

Furthermore this shows $\xi \le 0$.

### Proof

For $B_{2\rho }=B_{2\rho }(x) \subset \Omega '\subset \subset \Omega , 0< \delta <\delta _0$ and $0<\varepsilon \le \delta ^M$ then applying Lemma 3.7 we have

3.54 $$\begin{aligned} \begin{aligned} \int _{B_\rho }\left( \frac{\varepsilon |\nabla u_\varepsilon |^2}{2}-\frac{W(u_\varepsilon )}{\varepsilon }\right) _+&\le C\delta ^{p_3}\int _{B_{2\rho }}\left( \frac{\varepsilon |\nabla u_\varepsilon |^2}{2}+\frac{W(u_\varepsilon )}{\varepsilon }\right) \\&\quad +C\delta ^{-M}\varepsilon \int _{B_\rho }|f_\varepsilon |^2\\&\quad +C\delta ^{-M}\int _{B_\rho \cap \{|u_\varepsilon |\ge 1\}}\frac{W'(u_\varepsilon )^2}{\varepsilon }\\&\quad +C\left( \frac{\varepsilon }{\rho }\right) \delta \rho ^n. \end{aligned}\nonumber \\ \end{aligned}$$

Proposition 3.6 gives us

$$\begin{aligned} \int _{\{|u_\varepsilon |\ge 1\} \cap B_\rho } W'(u_\varepsilon )^2&\le C_k( 1 + \rho ^{-2k}\varepsilon ^{2k}) \varepsilon ^2 \int _{B_{2\rho }} |f_\varepsilon |^2 \\ {}&\quad + C_k \rho ^{-2k} \varepsilon ^{2k} \int _{\{|u_\varepsilon | \ge 1\} \cap B_{2\rho }} W'(u_\varepsilon )^2 \end{aligned}$$

for all $k \in {\mathbb {N}}_0$. Choosing $k=2$ and applying the bound

$$\begin{aligned} \int _{\{|u_\varepsilon | \ge 1\} \cap B_{2\rho }} W'(u_\varepsilon )^2 \le C(\Omega ') \end{aligned}$$

and inserting these estimates into (3.54), we get

$$\begin{aligned} \int _{B_\rho }\left( \frac{\varepsilon |\nabla u_\varepsilon |^2}{2}-\frac{W(u_\varepsilon )}{\varepsilon }\right) _+&\le C\delta ^{p_3}\int _{B_{2\rho }}\left( \frac{\varepsilon |\nabla u_\varepsilon |^2}{2}+\frac{W(u_\varepsilon )}{\varepsilon }\right) \\ {}&\quad +C(\delta ^{-M}\varepsilon +\varepsilon ^2)\int _{B_\rho }|f_\varepsilon |^2\\&\quad +C\delta ^{-M}\varepsilon ^3+C\left( \frac{\varepsilon }{\rho }\right) \delta \rho ^n. \end{aligned}$$

By the Hölder inequality with exponent $q_0/2$, we estimate

3.55 $$\begin{aligned} \varepsilon \int _{B_{r/2}} |f_\varepsilon |^2&= \varepsilon ^2 \int _{B_{r/2}}\left( \frac{f_\varepsilon }{|\varepsilon |\nabla u_\varepsilon |} \right) ^2 \varepsilon |\nabla u_\varepsilon |^2\nonumber \\ {}&\le \varepsilon ^2 \left( \int _{B_{r/2}}\left| \frac{f_\varepsilon }{|\varepsilon |\nabla u_\varepsilon |} \right| ^{q_0} \varepsilon |\nabla u_\varepsilon |^2\right) ^{2/q_0}\left( \int _{B_{r/2}} \varepsilon |\nabla u_\varepsilon |^2 \right) ^\frac{q_0}{q_0-2}\nonumber \\ {}&\le \varepsilon ^2 C(\Lambda _0, E_0), \end{aligned}$$

and obtain

$$\begin{aligned} \int _{B_\rho }\left( \frac{\varepsilon |\nabla u_\varepsilon |^2}{2}-\frac{W(u_\varepsilon )}{\varepsilon }\right) _+&\le {\tilde{C}}\delta ^{p_3}+{\tilde{C}}\delta ^{-M}\varepsilon ^2+{\tilde{C}}\varepsilon ^2 +{\tilde{C}}\delta ^{-M}\varepsilon ^3+{\tilde{C}}\delta \varepsilon \\&\le {\tilde{C}} \delta . \end{aligned}$$

Letting $\varepsilon \rightarrow 0$ we get $\xi _{\varepsilon ,+}(B_\rho )\rightarrow 0$. $\square$

## Rectifiability

We will proceed by proving upper and lower density bounds for the energy measure. Combining the estimates obtained in the previous section, we get an upper bound on the density ratio of the limit energy measure.

### Theorem 4.1

If we consider $\Omega '\subset \subset \Omega$ and $r_0(\Omega '):=\min \left\{ 1,\frac{d(\Omega ',\partial \Omega )}{2}\right\}$ then for all $x_0\in \Omega ', 0<r<r_0$ there exists a function $\phi (\varepsilon )$ with $\lim _{\varepsilon \rightarrow 0} \phi (\varepsilon )=0$ such that

4.1 $$\begin{aligned} r^{-n}\mu _\varepsilon (B_r(x_0))\le C(\Lambda _0,\Omega ') + \frac{\phi (\varepsilon )}{r^n}. \end{aligned}$$

Letting $\varepsilon \rightarrow 0$ we get

$$\begin{aligned} r^{-n}\mu (B_r(x_0))\le C(\Lambda _0,\Omega '), \end{aligned}$$

where $\mu =\lim _{\varepsilon \rightarrow 0} \mu _\varepsilon$ is the weak-* limit of $\mu _\varepsilon =\left( \frac{\varepsilon |\nabla u_\varepsilon |^2}{2}+\frac{W(u_\varepsilon )}{\varepsilon }\right) dx$ in the sense of Radon measures.

### Proof

For the sake of simplicity we set $$ x_0=0$$ and set $$ B_\rho (0)=B_\rho $$. By the almost monotonicity formula (3.1), Lemma 3.8 and Holder’s inequality

4.2 $$\begin{aligned} \frac{d}{d\rho }\left( \frac{\mu _\varepsilon (B_\rho )}{\rho ^n}\right)&=-\frac{1}{\rho ^{n+1}}\xi _\varepsilon (B_{\rho })+\frac{\varepsilon }{\rho ^{n+2}}\int _{\partial B_\rho }\langle x,\nabla u\rangle ^2\nonumber \\ {}&\quad -\frac{1}{\rho ^{n+1}}\int _{B_\rho }\langle x,\nabla u\rangle f_\varepsilon . \end{aligned}$$

We estimate the last term above as follows

4.3 $$\begin{aligned} \frac{1}{\rho ^{n+1}}\left| \int _{B_\rho }\langle x,\nabla u\rangle f_\varepsilon \right|&\le \frac{1}{\rho ^{n+1}}\int _{B_{\rho }}|\langle x,\nabla u\rangle |\left| \frac{f_\varepsilon }{\varepsilon |\nabla u|}\right| \varepsilon |\nabla u|\nonumber \\ {}&\le \frac{1}{\rho ^n}\int _{B_{\rho }}\left| \frac{f_\varepsilon }{\varepsilon |\nabla u|}\right| \varepsilon |\nabla u|^2\nonumber \\&\le \frac{1}{\rho ^n}\left( \int _{B_{\rho }}\left| \frac{f_\varepsilon }{\varepsilon |\nabla u|}\right| ^{q_0}\varepsilon |\nabla u|^2\right) ^\frac{1}{q_0}\left( \int _{B_\rho }\varepsilon |\nabla u|^2\right) ^\frac{q_0-1}{q_0}\nonumber \\ {}&\le \left( \frac{1}{\rho ^n}\right) ^\frac{1}{q_0}\left( \int _{B_{\rho }}\left| \frac{f_\varepsilon }{\varepsilon |\nabla u|}\right| ^{q_0}\varepsilon |\nabla u|^2\right) ^\frac{1}{q_0}\nonumber \\&\quad \left[ \frac{1}{\rho ^n}\right] ^\frac{q_0-1}{q_0}\left( 2\mu _\varepsilon (B_\rho )\right) ^\frac{q_0-1}{q_0}\nonumber \\&\le C(\Lambda _0)\rho ^{-\frac{n}{q_0}}\left( \frac{\mu _\varepsilon (B_\rho )}{\rho ^n}\right) ^\frac{q_0-1}{q_0}\nonumber \\&\le C(\Lambda _0)\rho ^{-\frac{n}{q_0}}\left( 1+\frac{\mu _\varepsilon (B_\rho )}{\rho ^n}\right) \end{aligned}$$

where we used the inequality $a^{1-\frac{1}{q_0}} \le 1+a$ which holds for all $a \ge 0$. Inserting this inequality into (4.2) and discarding the positive second term on the right had side, we get

4.4 $$\begin{aligned} \frac{d}{d\rho }\left( 1+\frac{\mu _\varepsilon (B_\rho )}{\rho ^n}\right)&=\frac{d}{d\rho }\left( \frac{\mu _\varepsilon (B_\rho )}{\rho ^n}\right) \ge -\frac{1}{\rho ^{n+1}}\xi _\varepsilon (B_{\rho })\nonumber \\ {}&\quad - C(\Lambda _0)\rho ^{-\frac{n}{q_0}}\left( 1+\frac{\mu _\varepsilon (B_\rho )}{\rho ^n}\right) . \end{aligned}$$

Multiplying both sides by $\exp \left( \int C(\Lambda _0)\rho ^{-\frac{n}{q_0}} d\rho \right) = \exp \left( \frac{q_0}{q_0-n} C(\Lambda _0)\rho ^{1-\frac{n}{q_0}}\right)$ we have

$$\begin{aligned}&\frac{d}{d\rho }\left[ \exp \left( \frac{q_0}{q_0-n} C(\Lambda _0)\rho ^{1-\frac{n}{q_0}}\right) \left( 1+\frac{\mu _\varepsilon (B_\rho )}{\rho ^n}\right) \right] \\&\quad \ge - \exp \left( \frac{q_0}{q_0-n} C(\Lambda _0)\rho ^{1-\frac{n}{q_0}}\right) \frac{\xi _\varepsilon (B_{\rho })}{\rho ^{n+1}}. \end{aligned}$$

Integrating from *r* to $r_0$ gives

$$\begin{aligned}&\exp \left( \frac{q_0}{q_0-n} C(\Lambda _0)r_0^{1-\frac{n}{q_0}}\right) \left( 1+\frac{\mu _\varepsilon (B_{r_0})}{r_0^n}\right) - \exp \left( \frac{q_0}{q_0-n} C(\Lambda _0)r^{1-\frac{n}{q_0}}\right) \left( 1+\frac{\mu _\varepsilon (B_r)}{r^n}\right) \\&\quad \ge -\int _r^{r_0} \exp \left( \frac{q_0}{q_0-n} C(\Lambda _0)\rho ^{1-\frac{n}{q_0}}\right) \frac{\xi _{\varepsilon ,+}(B_{\rho })}{\rho ^{n+1}}\\&\quad \ge -\exp \left( \frac{q_0}{q_0-n} C(\Lambda _0)r_0^{1-\frac{n}{q_0}}\right) \int _r^{r_0} \frac{\xi _{\varepsilon ,+}(B_{\rho })}{\rho ^{n+1}}. \end{aligned}$$

Namely

4.5 $$\begin{aligned} \exp \left( \frac{q_0}{q_0-n} C(\Lambda _0)r_0^{1-\frac{n}{q_0}}\right) \left( 1+\frac{\mu _\varepsilon (B_{r_0})}{r_0^n}\right) -\frac{\mu _\varepsilon (B_r)}{r^n}&\ge - C(\Lambda _0,\Omega ')\int _r^{r_0} \frac{\xi _{\varepsilon ,+}(B_{\rho })}{\rho ^{n+1}}\nonumber \\ {}&\ge - C(\Lambda _0,\Omega ')\int _r^{r_0} \frac{\xi _{\varepsilon ,+}(B_{r_0})}{\rho ^{n+1}}, \end{aligned}$$

where we used $\exp \left( \frac{q_0}{q_0-n} C(\Lambda _0)r^{1-\frac{n}{q_0}}\right) >1$ for $r>0$. Passing to the limit as $\varepsilon \rightarrow 0$ and using Lemma 3.8, we have

$$\begin{aligned} \frac{\mu (B_r)}{r^n}\le C(\Lambda _0,\Omega ',n,q_0). \end{aligned}$$

$$\square $$

Next, we obtain estimates of the discrepancy measure for each $\varepsilon$.

### Proposition 4.2

Let $\delta =\rho ^\gamma ,\varepsilon \le \rho \le r$ for $0<\gamma <\frac{1}{M}\le \frac{1}{2}$, we have $\delta ^{-M}\varepsilon \le \rho ^{1-M\gamma }\le 1$. For $B_{3\rho ^{1-\beta }}(x)\subset \subset \Omega$, we have

4.6 $$\begin{aligned}&\rho ^{-n-1} \xi _{\varepsilon ,+}(B_\rho (x))\le C\rho ^{p_3\gamma -n-1}\mu _\varepsilon (B_{2\rho }(x)) +{\tilde{C}}_k\varepsilon \rho ^{-M\gamma -n-1}\int _{B_{3\rho ^{1-\beta }}(x)}|f_\varepsilon |^2 \nonumber \\&\quad +{\tilde{C}}_\beta \varepsilon \rho ^{\gamma -2}\left( 1+\int _{\{|u_\varepsilon | \ge 1\} \cap B_{3r^{1-\beta }}(x)} W'(u_\varepsilon )^2\right) . \end{aligned}$$

### Proof

For $0<\gamma <\frac{1}{M}\le \frac{1}{2}$, by choosing $\delta ^{-M}\varepsilon \le \rho ^{1-M\gamma }\le 1$ we get $\max \{2, 1+ \delta ^{-M}\varepsilon \} =2$. Therefore substituting $\delta = \rho ^\gamma$ into Lemma 3.7 we have

$$\begin{aligned} \begin{aligned} \rho ^{-n-1}\xi _{\varepsilon ,+}(B_\rho )&= \rho ^{-n-1}\int _{B_\rho (x)}\left( \frac{\varepsilon |\nabla u_\varepsilon |^2}{2}-\frac{W(u_\varepsilon )}{\varepsilon }\right) _+\nonumber \\&\le C\rho ^{p_3\gamma -n-1}\int _{B_{2\rho }(x)}\left( \frac{\varepsilon |\nabla u_\varepsilon |^2}{2}+\frac{W(u_\varepsilon )}{\varepsilon }\right) \\&\quad +C\varepsilon \rho ^{-M\gamma -n-1}\int _{B_{2\rho }(x)}|f_\varepsilon |^2\\&\quad +C\varepsilon ^{-1}\rho ^{-M\gamma -n-1}\int _{B_{2\rho }(x)\cap \{|u_\varepsilon |\ge 1\}}W'(u_\varepsilon )^2+C\varepsilon \rho ^{\gamma -2}. \end{aligned} \end{aligned}$$

On the other hand we have by Proposition 3.6 with $r:=d(B_{2\rho }(x),\partial B_{3\rho ^{1-\beta }}(x))=3 \rho ^{1-\beta } - 2 \rho \ge \rho ^{1-\beta }$

$$\begin{aligned} \int _{\{|u_\varepsilon |\ge 1\} \cap B_{2\rho }} W'(u_\varepsilon )^2&\le C_k( 1 + \rho ^{-2k(1-\beta )}\varepsilon ^{2k}) \varepsilon ^2 \int _{B_{3\rho ^{1-\beta }} } |f_\varepsilon |^2 \\&\quad + C_k \rho ^{-2k(1-\beta )} \varepsilon ^{2k} \int _{\{|u_\varepsilon | \ge 1\} \cap B_{3\rho ^{1-\beta }}} W'(u_\varepsilon )^2. \end{aligned}$$

Substituting this into our above estimate, we get

$$\begin{aligned} \rho ^{-n-1}\xi _{\varepsilon ,+}(B_\rho )&\le C\rho ^{p_3\gamma -n-1}\int _{B_{2\rho }(x)}\left( \frac{\varepsilon |\nabla u_\varepsilon |^2}{2}+\frac{W(u_\varepsilon )}{\varepsilon }\right) \\&\quad +{\tilde{C}}_k\varepsilon \rho ^{-M\gamma -n-1}\int _{B_{3\rho ^{1-\beta }}(x)}|f_\varepsilon |^2\nonumber \\&\quad +C\varepsilon ^{-1}\rho ^{-M\gamma -n-1}{\tilde{C}}_k\rho ^{2k\beta -2k}\varepsilon ^{2k} \int _{\{|u_\varepsilon | \ge 1\} \cap B_{3\rho ^{1-\beta }}(x)} W'(u_\varepsilon )^2\\&\quad +C\varepsilon \rho ^{\gamma -2}\nonumber \\&\le C\rho ^{p_3\gamma -n-1}\mu _\varepsilon (B_{2\rho }(x))+{\tilde{C}}_k\varepsilon \rho ^{-M\gamma -n-1}\int _{B_{3\rho ^{1-\beta }}(x)}|f_\varepsilon |^2\\&\quad +C\left( \varepsilon \rho ^{\gamma -2}+\varepsilon ^{-1}\rho ^{-M\gamma -n-1}\varepsilon ^{2k\beta }\int _{\{|u_\varepsilon | \ge 1\} \cap B_{3\rho ^{1-\beta }}(x)} W'(u_\varepsilon )^2\right) \nonumber \\&\le C\rho ^{p_3\gamma -n-1}\mu _\varepsilon (B_{2\rho }(x))+{\tilde{C}}_{k,\beta }\varepsilon \rho ^{-M\gamma -n-1}\int _{B_{3\rho ^{1-\beta }}(x)}|f_\varepsilon |^2\\&\quad +{\tilde{C}}_\beta \varepsilon \rho ^{\gamma -2}\left( 1+\int _{\{|u_\varepsilon | \ge 1\} \cap B_{3\rho ^{1-\beta }}(x)} W'(u_\varepsilon )^2\right) , \end{aligned}$$

where we have chosen $-M\gamma -n+2k\beta +1 \ge \gamma -2$ or $k>\frac{\gamma -2+M\gamma +n+1}{2\beta }$ sufficiently large. $\square$

In the following theorem we prove the density lower bound for the limit measure.

### Theorem 4.3

There exists ${\bar{\theta }}>0$ such that for any $\Omega '\subset \subset \Omega$ and $r_1(\Omega ')\le \frac{d(\Omega ',\partial \Omega )}{2}$ sufficiently small, we have

$$\begin{aligned} r^{-n}\mu (B_r(x))\ge {\bar{\theta }}-Cr^\gamma , \end{aligned}$$

for some $\gamma >0$, and all $x\in {{\,\textrm{spt}\,}}\mu \cap \Omega '$ and $0<r\le r_1$. In particular,

$$\begin{aligned} \theta ^n_*(\mu )\ge \frac{{\bar{\theta }}}{\omega _n} \end{aligned}$$

for $$\mu $$-a.e. in $$\Omega $$.

### Proof

Without loss of generality, we assume $0\in {{\,\textrm{spt}\,}}\mu \cap \Omega '$ and want to prove a density lower bound at 0. We first integrate (4.4) from *s* to *r*.

4.7 $$\begin{aligned} \begin{aligned} \frac{\mu _\varepsilon (B_r(x))}{r^n}-\frac{\mu _\varepsilon (B_s(x))}{s^n}&\ge -\int _s^r\frac{1}{\rho ^{n+1}}\xi _{\varepsilon ,+}(B_{\rho }(x))d\rho \\&\quad -\int _s^rC(\Lambda _0)\rho ^{-\frac{n}{q_0}}\left( \frac{\mu _\varepsilon (B_\rho (x))}{\rho ^n}\right) ^\frac{q_0-1}{q_0}. \end{aligned} \end{aligned}$$

By (4.6) in Proposition 4.2, the discrepancy term

4.8 $$\begin{aligned} \begin{aligned} -\int _s^r \rho ^{-n-1} \xi _{\varepsilon ,+}(B_\rho (x))&\ge -\int _s^rC\rho ^{p_3\gamma -n-1}\mu _\varepsilon (B_{2\rho }(x))\\&\quad -\int _s^r{\tilde{C}}_k\varepsilon \rho ^{-M\gamma -n-1}\int _{B_{3\rho ^{1-\beta }}(x)}|f_\varepsilon |^2\\&\quad -\int _s^r{\tilde{C}}_\beta \varepsilon \rho ^{\gamma -2}\left( 1+\int _{\{|u_\varepsilon | \ge 1\} \cap \Omega } W'(u_\varepsilon )^2\right) . \end{aligned}\nonumber \\ \end{aligned}$$

By the $\varepsilon$-Upper Density Bound (4.1) we get

$$\begin{aligned} -\int _s^r\rho ^{p_3\gamma -n-1}\mu _\varepsilon (B_{2\rho }(x))&= - \int _s^r2^n\rho ^{p_3\gamma -1} \frac{\mu _\varepsilon (B_{2\rho }(x)) }{( 2 \rho )^n} \\&\ge - \int _s^r2^n\rho ^{p_3\gamma -1} \left( C(\Lambda _0,\Omega ') + \frac{\phi (\varepsilon )}{\rho ^n} \right) \\&\ge - C(\Lambda _0,\Omega ') \left( r^{p_3\gamma } - s^{p_3\gamma }\right) \\&\quad - \frac{\phi (\varepsilon )}{p_3\gamma -n+1}\left( r^{p_3\gamma -n} - s^{p_3\gamma -n } \right) . \end{aligned}$$

The last term in (4.8) may be estimated as follows

$$\begin{aligned} -\int _s^r{\tilde{C}}_\beta \varepsilon \rho ^{\gamma -2}\left( 1+\int _{\{|u_\varepsilon | \ge 1\} \cap \Omega } W'(u_\varepsilon )^2\right) \ge -\tilde{C}_\beta \int _s^t \rho ^{\gamma -1} d \rho \le -\tilde{C}_\beta ( r^\gamma -s^\gamma ). \end{aligned}$$

Using the bound

$$\begin{aligned} \left( \frac{\mu _\varepsilon (B_\rho (x))}{\rho ^n}\right) ^\frac{q_0-1}{q_0} \le \left( 1+\frac{\mu _\varepsilon (B_\rho (x))}{\rho ^n}\right) \end{aligned}$$

and the $\varepsilon$-Upper Density Bound (4.1), we get

$$\begin{aligned} -\int _s^rC(\Lambda _0)\rho ^{-\frac{n}{q_0}}\left( \frac{\mu _\varepsilon (B_\rho (x))}{\rho ^n}\right) ^\frac{q_0-1}{q_0}&\ge -\int _s^r C(\Lambda _0,\Omega ')\rho ^{-\frac{n}{q_0}} \left( 1+\frac{\mu _\varepsilon (B_\rho (x))}{\rho ^n}\right) \\&\ge -\int _s^r C(\Lambda _0,\Omega ')\rho ^{-\frac{n}{q_0}} \left( 1+C(\Lambda _0,\Omega ') + \frac{\phi (\varepsilon )}{\rho ^n}\right) \\&\ge -C(\Lambda _0,\Omega ')\left( r^{1 - \frac{n}{q_0}}-s^{1-\frac{n}{q_0}}\right) \\&\quad - C(\Lambda _0,\Omega ')\phi (\varepsilon ) \left( r^{1 -n - \frac{n}{q_0}}-s^{1-n-\frac{n}{q_0}}\right) . \end{aligned}$$

Thus, plug all the above estimates of terms in (4.7), we get

4.9 $$\begin{aligned} \frac{\mu _\varepsilon (B_r(x))}{r^n}-\frac{\mu _\varepsilon (B_s(x))}{s^n}&\ge - C(\Lambda _0,\Omega ') \left( r^{p_3\gamma } - s^{p_3\gamma }\right) \nonumber \\&\quad - \frac{\phi (\varepsilon )}{p_3\gamma -n+1}\left( r^{p_3\gamma -n} - s^{p_3\gamma -n } \right) \nonumber \\&\quad -\int _s^r{\tilde{C}}_{\beta }\varepsilon \rho ^{-M\gamma -n-1}\left( \int _{B_{3\rho ^{1-\beta }}(x)}|f_\varepsilon |^2\right) d\rho -\tilde{C}_\beta (r^\gamma -s^\gamma ) \nonumber \\&\quad -C(\Lambda _0,\Omega ')\left( r^{1 - \frac{n}{q_0}}-s^{1-\frac{n}{q_0}}\right) \nonumber \\&\quad - C(\Lambda _0,\Omega ')\phi (\varepsilon ) \left( r^{1-n - \frac{n}{q_0}}-s^{1-n-\frac{n}{q_0}}\right) . \end{aligned}$$

Next, we estimate the term $\int _s^r{\tilde{C}}_\beta \varepsilon \rho ^{-M\gamma -n-1}\left( \int _{B_{3\rho ^{1-\beta }}(x)}|f_\varepsilon |^2\right) d\rho$ in the following claim. $\square$

### Claim

There exists $x\in B_\frac{r}{2}$ such that

4.10 $$\begin{aligned} \varepsilon ^{-n}\mu _\varepsilon (B_\varepsilon (x))\ge 2{\bar{\theta }}_0>{\bar{\theta }}_0\ge \int _\varepsilon ^{\frac{r}{4}}{\tilde{C}}_\beta \varepsilon \rho ^{-M\gamma -n-1}\left( \int _{B_{3\rho ^{1-\beta }}(x)}|f_\varepsilon |^2\right) d\rho , \end{aligned}$$

for some universal constant ${\bar{\theta }}_0>0$.

### Proof of Claim

Consider a point $x\in B_\frac{r}{2}$ with $|u_\varepsilon (x)|\le 1-\tau$, for some $0<\tau <1$. We can assume $\varepsilon ^{-n}\mu _\varepsilon (B_\varepsilon (x))\le 1$(otherwise the conclusion automatically follows), and so

$$\begin{aligned} \varepsilon ^{-n-1}\int _{B_\varepsilon (x)}u_\varepsilon ^p\le \varepsilon ^{-n-1}\int _{B_\varepsilon (x)}c_0^p\le c_0^p\omega _{n+1}, \forall p>1. \end{aligned}$$

From Theorem 3.2 we have

$$\begin{aligned} \varepsilon ^\frac{1}{2}\Vert u\Vert _{C^{0,\frac{1}{2}}(B_{1-\varepsilon }(x))}\le C, \end{aligned}$$

and thus

$$\begin{aligned} |u_\varepsilon |\le 1-\frac{\tau }{2}, \quad \text { in } B_{\frac{\tau ^2\varepsilon }{4C^2}}(x). \end{aligned}$$

So since $W(t) =(1-t^2)^2 =(1+t)^2(1-t)^2$ we find in $B_{\frac{\tau ^2\varepsilon }{4C^2}}(x)$

$$\begin{aligned} W(u_\varepsilon )=(1+|u_\varepsilon |)^2(1-|u_\varepsilon |)^2 \ge \frac{\tau ^2}{4} \end{aligned}$$

4.11 $$\begin{aligned} \varepsilon ^{-n}\mu _\varepsilon (B_\varepsilon (x))\ge \varepsilon ^{-n}\int _{B_{\frac{\tau ^2\varepsilon }{4C^2}}(x)}\frac{W(u_\varepsilon )}{\varepsilon }&\ge \varepsilon ^{-n-1}\omega _{n+1}\left( \frac{\tau ^2\varepsilon }{4C^2}\right) ^{n+1}\frac{\tau ^2}{4}\nonumber \\&\ge C_n\tau ^{2n+4}. \end{aligned}$$

Denote

$$\begin{aligned} 2{\bar{\theta }}_0:=\min \{1,C_n\tau ^{2n+4}\}, \end{aligned}$$

then for $x\in B_{\frac{r}{2}}\cap \{|u_\varepsilon |\le 1-\tau \}$ the first inequality in the conclusion of the claim holds. Applying the error estimates Proposition 3.5 with the choice $\Omega '=B_\frac{r}{4}$ and $\Omega =B_\frac{r}{2}$, for sufficiently small $\tau$

$$\begin{aligned} \mu _\varepsilon (B_\frac{r}{4})&=\mu _\varepsilon \left( B_\frac{r}{4}\cap \{|u_\varepsilon |<1-\tau \}\right) +\mu _\varepsilon \left( B_\frac{r}{4}\cap \{|u_\varepsilon |\ge 1-\tau \}\right) \\&\le C\mu _\varepsilon \left( B_\frac{r}{4}\cap \{|u_\varepsilon |<1-\tau \}\right) +C\varepsilon \int _{B_{\frac{r}{2}}}|f_\varepsilon |^2+C\varepsilon (\tau r^n+\tau ^2 r^{n-1})\\ {}&\quad +Cr^{-2}\varepsilon . \end{aligned}$$

Notice by (3.55), the second term $\varepsilon \int _{B_{r/2}} |f_\varepsilon |^2 \le \varepsilon ^2 C(\Lambda _0, E_0)$. So the last three terms are at most of order $O(\varepsilon )$. Hence, as $0\in {{\,\textrm{spt}\,}}\mu$, by passing to limit $\varepsilon \rightarrow 0$ we have

$$\begin{aligned} 0<\mu (B_{\frac{r}{4}})\le \liminf _{\varepsilon \rightarrow 0}\mu _\varepsilon (B_\frac{r}{4})\le \liminf _{\varepsilon \rightarrow 0}\mu _\varepsilon \left( B_\frac{r}{4}\cap \{|u_\varepsilon |<1-\tau \}\right) . \end{aligned}$$

And in the set $\{|u_\varepsilon |\le 1-\tau \}$, we get by Lemma 3.8 that

4.12 $$\begin{aligned}&\liminf _{\varepsilon \rightarrow 0}\varepsilon ^{-1}{\mathcal {L}}^{n+1}(B_\frac{r}{2}\cap \{|u_\varepsilon |\le 1-\tau \})\nonumber \\&\quad \ge \liminf _{\varepsilon \rightarrow 0}\varepsilon ^{-1}\int _{B_\frac{r}{2}\cap \{|u_\varepsilon |\le 1-\tau \}}\frac{W(u_\varepsilon )}{\tau ^2}\nonumber \\&\quad =\liminf _{\varepsilon \rightarrow 0}\frac{1}{\tau ^2}\left( \mu _\varepsilon -\xi _\varepsilon \right) (B_\frac{r}{2}\cap \{|u_\varepsilon |\le 1-\tau \})\nonumber \\&\quad \ge \frac{1}{\tau ^2}\liminf _{\varepsilon \rightarrow 0}\mu _\varepsilon \left( B_\frac{r}{4}\cap \{|u_\varepsilon |<1-\tau \}\right) {-}\liminf _{\varepsilon \rightarrow 0}\frac{1}{\tau ^2}\xi _{\varepsilon ,+}(B_\frac{r}{2}\cap \{|u_\varepsilon |\le 1-\tau \})\nonumber \\&\quad \ge \frac{\mu (B_\frac{r}{4})}{\tau ^2}>0. \end{aligned}$$

(This guarantees we can always choose such a point $x\in B_\frac{r}{2}$ with $|u_\varepsilon (x)|\le 1-\tau$ if $0\in {{\,\textrm{spt}\,}}\mu$.) To complete the proof, we define for $0<\rho <r_1$ the convolution

$$\begin{aligned} \omega _{\varepsilon ,\rho }(x):=\rho ^{-n-1}\left( \chi _{B_\rho }*\frac{1}{\varepsilon }|f_\varepsilon |^2\right) (x)=\rho ^{-n-1}\int _{B_\rho (x)}\frac{1}{\varepsilon }|f_\varepsilon |^2, \end{aligned}$$

with

$$\begin{aligned} \Vert \omega _{\varepsilon ,\rho }(x)\Vert _{L^1(B_\frac{r_1}{2})}\le \int _{B_{\frac{r_1}{2}+r_1}}\frac{1}{\varepsilon }|f_\varepsilon |^2\le C(\Lambda _0,E_0)<\infty , \end{aligned}$$

by (3.55). Denote by $\omega _\varepsilon (x):=\int _0^{r_1}\omega _{\varepsilon ,\rho }(x)d\rho$, we have

$$\begin{aligned} \Vert \omega _\varepsilon (x)\Vert _{L^1(B_\frac{r_0}{2})}\le r_1 C(\Lambda _0,E_0)<\infty . \end{aligned}$$

Now we can estimate the term on the right hand side in the claim, by a change of variables $t=3\rho ^{1-\beta }$. Here $\beta :=\beta (r_1)$ is chosen small enough such that $3\left( \frac{r_1}{4}\right) ^{1-\beta }\le r_1$. We calculate, setting $t = 3 \rho ^{1-\beta }$

$$\begin{aligned}&\int _\varepsilon ^{\frac{r}{4}}\rho ^{-M\gamma -n-1}\left( \int _{B_{3\rho ^{1-\beta }}(x)}\frac{1}{\varepsilon }|f_\varepsilon |^2\right) d\rho \\&\quad =\int _{3\varepsilon ^{1-\beta }}^{3\left( \frac{r}{4}\right) ^{1-\beta }}\left( \frac{t}{3}\right) ^\frac{-M\gamma -n-1}{1-\beta }\left( \int _{B_t(x)}\frac{1}{\varepsilon }|f_\varepsilon |^2\right) d\left( \frac{t}{3}\right) ^\frac{1}{1-\beta }\\&\quad \le C_\beta \int _{3\varepsilon ^{1-\beta }}^{3\left( \frac{r}{4}\right) ^{1-\beta }} t^\frac{-M\gamma -n-1+\beta }{1-\beta }\left( \int _{B_t}\frac{1}{\varepsilon }|f_\varepsilon |^2\right) dt\\&\quad \le C_\beta \int _{3\varepsilon ^{1-\beta }}^{3\left( \frac{r_1}{4}\right) ^{1-\beta }} t^{\frac{-M\gamma -n-1+\beta }{1-\beta }+(n+1)}\omega _{\varepsilon ,t}(x)dt. \end{aligned}$$

We find

$$\begin{aligned} \frac{-M\gamma -n-1+\beta }{1-\beta }+(n+1)= \frac{- M \gamma - n \beta }{1-\beta }< 0 \end{aligned}$$

so that $t^{\frac{- M \gamma - n \beta }{1-\beta } }$ is a decreasing function. Hence we get the bound

4.13 $$\begin{aligned} \begin{aligned} \int _\varepsilon ^{\frac{r}{4}}\rho ^{-M\gamma -n-1}\left( \int _{B_{3\rho ^{1-\beta }}(x)}\frac{1}{\varepsilon }|f_\varepsilon |^2\right) d\rho&\le C_\beta \int _{3\varepsilon ^{1-\beta }}^{3\left( \frac{r_1}{4}\right) ^{1-\beta }} \left( 3\varepsilon ^{1-\beta }\right) ^\frac{-M\gamma -n\beta }{1-\beta }\omega _{\varepsilon ,t}(x)dt\\&\le C_\beta \varepsilon ^{-M\gamma -n\beta }\int _0^{r_1}\omega _{\varepsilon ,t}(x)dt\\&\le C_\beta \varepsilon ^{-M\gamma -n\beta }\omega _\varepsilon (x). \end{aligned} \end{aligned}$$

Choosing $M\gamma <\frac{1}{2}$ and $\beta$ sufficiently small so that $M\gamma +n\beta <\frac{1}{2}$, and applying the weak $L^1$ inequality for the distribution function and (4.13), we get for some ${\tilde{C}}_\beta$ depending on $\beta$

4.14 $$\begin{aligned}&{\mathcal {L}}^{n+1}\left( B_{\frac{r}{2}}\cap \left\{ \int _\varepsilon ^{\frac{r}{4}}{\tilde{C}}_\beta \varepsilon \rho ^{-M\gamma -n-1}\left( \int _{B_{3\rho ^{1-\beta }}(x)}|f_\varepsilon |^2\right) d\rho \ge {\bar{\theta }}_0\right\} \right) \nonumber \\&\quad \le {\mathcal {L}}^{n+1}\left( B_{\frac{r}{2}}\cap \left\{ C_\beta \varepsilon ^2\varepsilon ^{-M\gamma -n\beta }\omega _\varepsilon (x)\ge {\bar{\theta }}_0\right\} \right) \nonumber \\&\quad \le C_\beta \varepsilon ^{2-(M\gamma +n\beta )}{\bar{\theta }}_0^{-1}\Vert \omega _\varepsilon \Vert _{L^1(B_\frac{r}{2})}\nonumber \\&\quad \le C_\beta \varepsilon ^{2-(M\gamma +n\beta )}{\bar{\theta }}_0^{-1}\Vert \omega _{\varepsilon ,\rho }(x)\Vert _{L^1(B_\frac{r_1}{2})}\nonumber \\&\quad \le C_\beta \varepsilon ^{2-(M\gamma +n\beta )}{\bar{\theta }}_0^{-1} C(\Lambda _0,E_0)\nonumber \\&\quad \rightarrow 0, \end{aligned}$$

as $\varepsilon \rightarrow 0$. This guarantees we can always choose such a point $x'\in B_\frac{r}{2}$ with

$$\begin{aligned} \left\{ \int _\varepsilon ^{\frac{r}{4}}{\tilde{C}}_\beta \varepsilon \rho ^{-M\gamma -n-1}\left( \int _{B_{3\rho ^{1-\beta }}(x')}|f_\varepsilon |^2\right) d\rho \le {\bar{\theta }}_0\right\} . \end{aligned}$$

We can thus combine (4.12) with (4.14) to find an $x\in B_\frac{r}{2}$ so that the upper bound and lower bound in the claim holds. $\square$

With this claim, we proceed with the proof of the density lower bound. For the ${\bar{\theta }}_0$ obtained from the claim, we denote by $s:=\sup \{0\le \rho \le \frac{r}{4}:\frac{\mu _\varepsilon (B_\rho (x))}{\rho ^n}\ge 2{\bar{\theta }}_0\}$. And it is obvious from (4.11)

$$\begin{aligned} s\ge \varepsilon . \end{aligned}$$

By this choice of *s*, we have

$$\begin{aligned} \frac{\mu _\varepsilon (B_s(x))}{s^n}&\ge 2{\bar{\theta }}_0,\\ \frac{\mu _\varepsilon (B_\rho (x))}{\rho ^n}&\le 2{\bar{\theta }}_0, \forall \rho \in \left[ s,\frac{r}{4}\right] . \end{aligned}$$

Substituting $\frac{r}{4}$ for *r* in the integral form of the almost monotonicity formula (4.9), we get from (4.10) the following density lower bound

$$\begin{aligned} 2^n\left[ \frac{\mu _\varepsilon (B_\frac{r}{2}(x))}{\left( \frac{r}{2}\right) ^n}\right]&\ge \frac{\mu _\varepsilon (B_\frac{r}{4}(x))}{\left( \frac{r}{4}\right) ^n}\\&\ge \frac{\mu _\varepsilon (B_s(x))}{s^n} - C(\Lambda _0,\Omega ') \left( \left( \frac{r}{4}\right) ^{p_3\gamma } - s^{p_3\gamma }\right) - \frac{\phi (\varepsilon )}{p_3\gamma -n+1}\\&\quad \times \left( \left( \frac{r}{4}\right) ^{p_3\gamma -n} - s^{p_3\gamma -n} \right) -\int _s^{r/4}{\tilde{C}}_{\beta }\varepsilon \rho ^{-M\gamma -n-1}\left( \int _{B_{3\rho ^{1-\beta }}(x)}|f_\varepsilon |^2\right) d\rho \\&\quad -\tilde{C}_\beta (\left( \frac{r}{4}\right) ^\gamma -s^\gamma )\\&\quad -C(\Lambda _0, \Omega ')\left( \left( \frac{r}{4}\right) ^{1 - \frac{n}{q_0}}-s^{1-\frac{n}{q_0}}\right) \\&\quad - C(\Lambda _0,\Omega ')\phi (\varepsilon ) \left( \left( \frac{r}{4}\right) ^{1 -n - \frac{n}{q_0}}-s^{1-n-\frac{n}{q_0}}\right) \\&\ge 2 \overline{\theta }_0 - C(\Lambda _0,\Omega ')r^{\gamma _n} - C(\Lambda _0, \Omega ') \phi (\varepsilon ) r^{-n-\frac{n}{q_0} }\\&\quad -C(\Lambda _0,\Omega ') \phi (\varepsilon ) r^{p_3\gamma -n+1}-\overline{\theta }_0 \\&\ge \overline{\theta }_0 - C(\Lambda _0,\Omega ')r^{\gamma _n} - C(\Lambda _0, \Omega ') \phi (\varepsilon ) r^{-n-\frac{n}{q_0} }-C(\Lambda _0,\Omega ') \phi (\varepsilon ) r^{p_3\gamma -n+1}, \end{aligned}$$

where $\gamma _n:=\min \{p_3\gamma ,\gamma ,1-\frac{n}{q_0}\}>0$, and $\phi (\varepsilon )\rightarrow$ as $\varepsilon \rightarrow 0$ by Theorem 4.1. As $B_{\frac{r}{2}}(x) \subseteq B_r(0)$ we let $\varepsilon \rightarrow 0$ and get for some $\gamma _n>0$

$$\begin{aligned} \frac{\mu (\overline{B_r})}{r^n}\ge \limsup _{\varepsilon \rightarrow 0}\frac{\mu _\varepsilon (B_r)}{r^n}\ge \limsup _{\varepsilon \rightarrow 0}\frac{\mu _\varepsilon (B_{\frac{r}{2}}(x))}{r^n} \ge C_n{\bar{\theta }}_0- C_nr^{\gamma _n}. \end{aligned}$$

Approximating $r'\nearrow r$ we get for $0< r< r_1(\Omega ')$

$$\begin{aligned} \frac{\mu (B_r(0))}{r^n } \ge c_0 \overline{\theta }_0 \end{aligned}$$

and hence

$$\begin{aligned} \theta _*^n(\mu ) \ge \frac{\overline{\theta }}{\omega _n} \quad \mu \text {-a.e. in }\Omega . \end{aligned}$$

which completes the proof. $\square$

Before proving the rectifiability of the limit measure, we need to show that the full discrepancy vanishes as the limit $\varepsilon \rightarrow 0$.

### Proposition 4.4

$$ \begin{aligned} |\xi _\varepsilon |\rightarrow 0 \quad { \& } \quad |\xi |=0. \end{aligned}$$

### Proof

We first prove the lower *n*-dimensional density of the discrepancy measure vanishes. Namely

$$\begin{aligned} \theta _*^n(|\xi |)=\liminf _{\rho \rightarrow 0}\frac{|\xi |(B_\rho )}{\rho ^n}=0. \end{aligned}$$

If not, there exists $0<\rho _0,\delta <1$ and $B_{\rho _0}\subset \Omega$ such that

$$\begin{aligned} \frac{|\xi |(B_\rho (x))}{\rho ^n}\ge \delta ,\quad \forall 0<\rho \le \rho _0. \end{aligned}$$

Multiplying both sides of (4.2) by an integrating factor and integrating from *r* to $\rho _0$ as in the proof of Theorem 4.1 we get

$$\begin{aligned} C(\Lambda _0,\Omega ')\left( \frac{\mu _\varepsilon (B_{\rho _0})}{\rho _0^n}\right) -C(\Lambda _0,\Omega ')\left( \frac{\mu _\varepsilon (B_r)}{r^n}\right)&\ge -C(\Lambda _0,\Omega ')\int _r^{\rho _0}\frac{\xi _\varepsilon (B_{r_0})}{\rho ^{n+1}}d\rho . \end{aligned}$$

Using Lemma 3.8, that is $\xi _+=0$ and Theorem 4.1, we have when passing to the limit $\varepsilon \rightarrow 0$

$$\begin{aligned} {\tilde{C}}(\Lambda _0,\Omega ')&\ge C(\Lambda _0,\Omega ')\int _r^{\rho _0}\frac{\xi _-(B_\rho )}{\rho ^{n+1}}d\rho = C(\Lambda _0,\Omega ')\int _r^{\rho _0}\frac{\xi _-(B_\rho )+\xi _+(B_\rho )}{\rho ^{n+1}}d\rho \\&= C(\Lambda _0,\Omega ')\int _r^{\rho _0}\frac{|\xi |(B_\rho )}{\rho ^{n+1}}d\rho \\&\ge C(\Lambda _0,\Omega ')\int _r^{\rho _0}\frac{\delta }{\rho }d\rho \\&= C(\Lambda _0,\Omega ')\delta \ln \left( \frac{\rho _0}{r}\right) . \end{aligned}$$

This gives a contradiction by letting $r\rightarrow 0$. By the density lower bound Theorem 4.3 and differentiation theorem for measures, we have

$$\begin{aligned} D_\mu |\xi |(x)=\liminf _{\rho \rightarrow 0}\frac{|\xi |(B_\rho (x))}{\mu (B_\rho (x))}&\le \frac{\liminf _{\rho \rightarrow 0}\frac{|\xi |(B_\rho (x))}{\rho ^n}}{\limsup _{\rho \rightarrow 0}\frac{\mu (B_\rho (x))}{\rho ^n}}\\&\le \frac{\theta _*^n(|\xi |,x)\omega _n}{{\bar{\theta }}}=0 \end{aligned}$$

and this shows

$$\begin{aligned} |\xi |=D_\mu |\xi |\cdot \mu =0. \end{aligned}$$

$$\square $$

### Proposition 4.5

We choose a Borel measurable function $\nu _\varepsilon : \Omega \rightarrow \partial B_1(0)$ extending $\frac{\nabla u_\varepsilon }{| \nabla u_\varepsilon |}$ on $\nabla u_\varepsilon \ne 0$ and consider the varifold $V_\varepsilon =\mu _\varepsilon \otimes \nu _\varepsilon$ that is

4.15 $$\begin{aligned} \int _{\{| \nabla u |\ne 0\}} \phi \left( x,I - \frac{\nabla u(x)}{|\nabla u(x)|}\otimes \frac{\nabla u(x)}{|\nabla u(x)|}\right) d\mu _i(x), \quad \phi \in C_c(G_n(\Omega )). \end{aligned}$$

The first variation is given by

4.16 $$\begin{aligned} \delta V_\varepsilon ( \eta )&=-\int f_\varepsilon \langle \nabla u_\varepsilon ,\eta \rangle dx+\int \nabla \eta \left( \frac{\nabla u_\varepsilon }{|\nabla u_\varepsilon |},\frac{\nabla u_\varepsilon }{|\nabla u_\varepsilon |}\right) d\xi _\varepsilon , \nonumber \\&\qquad \forall \eta \in C_c^1(\Omega \times {\mathbb {R}}^{n+1}). \end{aligned}$$

### Proof

By Eq. (2.1), we have

$$\begin{aligned} \delta V_\varepsilon ( \eta )&= \int _{\Omega \times G(n+1,n)} {{\,\textrm{div}\,}}_S \eta (x) dV_\varepsilon (x,S) \\&= \int _{\Omega }( {{\,\textrm{div}\,}}\eta - \nabla \eta (\nu _\varepsilon ,\nu _\varepsilon ))d \mu _\varepsilon \\&= \int _{\Omega }( {{\,\textrm{div}\,}}\eta - \nabla \eta (\nu _\varepsilon ,\nu _\varepsilon )) \left( \frac{\varepsilon |\nabla u_\varepsilon |^2}{2} + \frac{W(u_\varepsilon )}{\varepsilon } \right) d {\mathcal {L}}^{n+1}. \end{aligned}$$

The Stress-Energy tensor for the Allen–Cahn equation is given by

$$\begin{aligned} T_{i j}&=\varepsilon \frac{\left| \nabla u_\varepsilon \right| ^2}{2} \delta _{i j}-\varepsilon \nabla _i u_\varepsilon \nabla _j u_\varepsilon +W\left( u_\varepsilon \right) \delta _{i j},\\ \nabla _i T_{i j}&=\varepsilon \nabla _i \nabla _k u_\varepsilon \nabla _k u_\varepsilon \delta _{ij}-\varepsilon \Delta u_\varepsilon \nabla _j u_\varepsilon -\varepsilon \nabla _i u_\varepsilon \nabla _i \nabla _k u_\varepsilon \\&\quad +W^{\prime }\left( u_\varepsilon \right) \nabla _i u_\varepsilon \delta _{ij}\\&=\left( -\varepsilon \Delta u_\varepsilon +W^{\prime }\left( u_\varepsilon \right) \right) \nabla _j u_\varepsilon . \end{aligned}$$

Now

$$\begin{aligned} T_{i j} \nabla _i \eta _j&=\left( \frac{\varepsilon | \nabla u_\varepsilon |^2}{2}+W\left( u_\varepsilon \right) \right) {{\,\textrm{div}\,}}\eta -\varepsilon \nabla \eta \left( \nabla u_\varepsilon , \nabla u_\varepsilon \right) \\&=\left( \frac{\varepsilon | \nabla u_\varepsilon |^2}{2}+W\left( u_\varepsilon \right) \right) {{\,\textrm{div}\,}}\eta -\nabla \eta (\nu _\varepsilon ,\nu _\varepsilon )\varepsilon |\nabla u_\varepsilon |^2. \end{aligned}$$

Integrating by parts, we get

$$\begin{aligned}&\int _{\Omega } \left( \frac{\varepsilon | \nabla u_\varepsilon |^2}{2}+W\left( u_\varepsilon \right) \right) {{\,\textrm{div}\,}}\eta -\nabla \eta (\nu _\varepsilon ,\nu _\varepsilon )\varepsilon |\nabla u_\varepsilon |^2 \\ {}&\quad = - \int _{\Omega } \nabla _i T_{i j} \eta _j\\&\quad = \int _\Omega \left( -\varepsilon \Delta u_\varepsilon +W^{\prime }\left( u_\varepsilon \right) \right) \langle \nabla u_\varepsilon , \eta \rangle . \end{aligned}$$

Hence inserting this into our expression for the first variation we get

$$\begin{aligned} \delta V_\varepsilon ( \eta )&= - \int _\Omega \left( - \varepsilon \Delta u_\varepsilon + \frac{W'(u_\varepsilon )}{\varepsilon } \right) \langle \nabla u_\varepsilon , \eta \rangle d {\mathcal {L}}^{n+1} + \int _{\Omega } \nabla \eta (\nu _\varepsilon ,\nu _\varepsilon ) d \xi _\varepsilon . \end{aligned}$$

$$\square $$

Combining Theorem 4.1, Theorem 4.3 and Proposition 4.4, we obtain

### Theorem 4.6

After passing to a subsequence, the associated varifolds $V_\varepsilon \rightarrow V$ where *V* is a rectifiable *n*-varifold with the weak mean curvature in $L_{loc}^{q_0}(\mu _V)$.

### Proof

We first compute the first variation of the associated varifolds $V_\varepsilon$ to the energy measure $\mu _\varepsilon$(c.f. [8, Proposition 4.10], [11, Equation 4.3]). For any $\eta \in C_0^1(\Omega ;{\mathbb {R}}^{n+1})$, using Proposition 4.5 and Proposition 4.4

4.17 $$\begin{aligned} \begin{aligned} |(\delta V)(\eta )|&=|\lim _{\varepsilon \rightarrow 0}(\delta V_\varepsilon )(\eta )|\\&=\left| \lim _{\varepsilon \rightarrow 0}\left( -\int f_\varepsilon \langle \nabla u_\varepsilon ,\eta \rangle dx+\int \nabla \eta \left( \frac{\nabla u_\varepsilon }{|\nabla u_\varepsilon |},\frac{\nabla u_\varepsilon }{|\nabla u_\varepsilon |}\right) d\xi _\varepsilon \right) \right| \\&\le \lim _{\varepsilon \rightarrow 0}\int |f_\varepsilon | |\nabla u_\varepsilon ||\eta | dx+\lim _{\varepsilon \rightarrow 0}\int |\nabla \eta |d|\xi _\varepsilon |\\&\le \lim _{\varepsilon \rightarrow 0}\int \left| \frac{f_\varepsilon }{\varepsilon |\nabla u|}\right| |\eta | \varepsilon |\nabla u_\varepsilon |^2dx\\&\le \lim _{\varepsilon \rightarrow 0}\left( \int \left| \frac{f_\varepsilon }{\varepsilon |\nabla u_\varepsilon |}\right| ^{q_0}\varepsilon |\nabla u_\varepsilon |^2\right) ^\frac{1}{q_0}\left( \int |\eta |^\frac{q_0}{q_0-1}\varepsilon |\nabla u_\varepsilon |^2\right) ^\frac{q_0-1}{q_0}\\&\le \Lambda _0^\frac{1}{q_0}\Vert \eta \Vert _{L^\frac{q_0}{q_0-1}(\mu _V)}\left( \le C(\Lambda _0,E_0)|\eta |\right) . \end{aligned}\nonumber \\ \end{aligned}$$

So we see the limit varifold has locally bounded first variation, combining with the density lower bound Theorem 4.3 we conclude the limit varifold is rectifiable by Allard’s rectifiability theorem. Moreover, the above calculation shows $\delta V$ is a bounded linear functional on $L_{loc}^\frac{q_0}{q_0-1}(\mu _V)$ and thus itself is in $L_{loc}^{q_0}(\mu _V)$. $\square$

## Integrality

In this section, we prove the integrality of the limit varifold.

### Theorem 5.1

Let $\mu$ be defined by (4.15). Then $\frac{1}{\alpha }\mu$ is an integral *n*-varifold where $\alpha =\int _{-\infty }^\infty (\tanh ' x)^2dx$ is the total energy of the heteroclinic 1-d solution.

From the previous section, we have already shown the limiting varifold *V* is rectifiable. And thus for a.e. $x_0\in {{\,\textrm{spt}\,}}\mu _V$, we have for any sequence $\rho _i\rightarrow 0$

$$\begin{aligned} {\mathcal {D}}_{\rho _i,\#}\circ {\mathcal {T}}_{x_0,\#}(\mu _V)\rightarrow \theta _{x_0} P_0,\quad \text { for some }P_0\in G(n+1,n), \end{aligned}$$

where ${\mathcal {D}}_{\rho _i}(x)=\rho _i^{-1}x$ and ${\mathcal {T}}_{x_0}(x)=x-x_0$ represent dilations and translations in ${\mathbb {R}}^{n+1}$ and $\theta _{x_0}$ is the density of $\mu _V$ at $x_0$. By choosing a sequence of rescaling factors $\rho _i$ such that

5.1 $$\begin{aligned} {\tilde{\varepsilon }}_i:=\frac{\varepsilon _i}{\rho _i}\rightarrow 0, \end{aligned}$$

the new sequence ${\tilde{u}}_{{\tilde{\varepsilon }}_i}(x):=u_{\varepsilon _i}(\rho _i x+x_0), {\tilde{f}}_{{\tilde{\varepsilon }}_i}(x):=\rho _i{\tilde{f}}_i(\rho _ix+x_0)$ satisfies

$$\begin{aligned} {\tilde{\varepsilon }}_i\Delta {\tilde{u}}_{{\tilde{\varepsilon }}_i}-\frac{W'({\tilde{u}}_{{\tilde{\varepsilon }}_i})}{{\tilde{\varepsilon }}_i}={\tilde{f}}_{{\tilde{\varepsilon }}_i} \end{aligned}$$

and the associated varifold ${\tilde{V}}_i$ of this new sequence ${\tilde{u}}_{{\tilde{\varepsilon }}_i}$ converges to $\theta _{x_0} P_0$. By (3.55), we also have

$$\begin{aligned} \frac{1}{{\tilde{\varepsilon }}_i}\int _{B_\rho } f_{{\tilde{\varepsilon }}_i}^2&\le C\left( \int _{B_\rho }\left( \frac{f_{{\tilde{\varepsilon }}_i}}{{\tilde{\varepsilon }}_i|\nabla u_{{\tilde{\varepsilon }}_i}|}\right) ^{q_0}{\tilde{\varepsilon }}_i|\nabla u_{{\tilde{\varepsilon }}_i}|^2\right) ^\frac{2}{q_0}\\&= C\left( \rho _i^{q_0+1-(n+1)}\int _{B_{\rho _i\rho }}\left( \frac{f_{\varepsilon _i}}{\varepsilon _i|\nabla u_{\varepsilon _i}|}\right) ^{q_0}\varepsilon _i|\nabla u_{\varepsilon _i}|^2\right) ^\frac{2}{q_0}\\&\le C \rho _i^\frac{2(q_0-n)}{q_0}\rightarrow 0, \end{aligned}$$

as $q_0>n$. Furthermore, by choosing more carefully so that $\rho _i:={\tilde{\varepsilon }}_i^\frac{(n-1)q_0}{2(q_0-n)}=\varepsilon _i^\frac{1}{1+\frac{2(q_0-n)}{(n-1)q_0}}$, we have

$$\begin{aligned} \frac{1}{{\tilde{\varepsilon }}_i}\int _{B_\rho } f_{{\tilde{\varepsilon }}_i}^2\le {\tilde{\varepsilon }}_i^{n-1},\quad \text { for } \rho >{\tilde{\varepsilon }}_i \end{aligned}$$

and thus

5.2 $$\begin{aligned} \frac{1}{{\tilde{\varepsilon }}_i}\int _{B_\rho } f_{{\tilde{\varepsilon }}_i}^2\le \rho ^{n-1}. \end{aligned}$$

Therefore we have reduced Theorem 5.1 to the following proposition

### Proposition 5.2

If the limit varifold is $\theta _0{\mathcal {H}}^n\lfloor P_0$ for some $P_0\in G(n+1,n)$ and $\theta _0>0$, then $\alpha ^{-1}\theta _0$ is a nonnegative integer, where $\alpha =\int _{-\infty }^\infty (\tanh ' x)^2dx$ is the total energy of the heteroclinic 1-d solution.

In order to prove Proposition 5.2, we need two lemmas. The first Lemma 5.5 is a multi-sheet monotonicity formula (c.f. [1, Theorem 6.2] for the version for integral varifolds, which is used to prove the integrality of the limits of sequences of integral varifolds). The second Lemma 5.7 says at small scales, the energy of each layers are almost integer multiple of the 1-d solution. We first gather some apriori bounds on energy ratio for $\mu _\varepsilon$.

### Proposition 5.3

Let $\delta =\rho ^\gamma ,\varepsilon \le \rho \le r$ for $0<\gamma <\frac{1}{M}\le \frac{1}{2}$, we have $\delta ^{-M}\varepsilon \le \rho ^{1-M\gamma }\le 1$. Furthermore we choose $r:=d(B_{2\rho }(x),\partial B_{3\rho ^{1-\beta }}(x))\ge \rho ^{1-\beta }$. Then

5.3 $$\begin{aligned} \begin{aligned} Cr^{-n}\mu _\varepsilon (B_r(x))&\ge s^{-n}\mu _\varepsilon (B_s(x))-C\int _s^r\rho ^{p_3\gamma -n-1}\mu _\varepsilon (B_{2\rho }(x))d\rho \\&\quad -C_\beta \varepsilon \int _s^r\rho ^{-M\gamma -n-1}\left( \int _{B_{3\rho ^{1-\beta }}(x)}|f_\varepsilon |^2\right) d\rho \\&\quad -{\tilde{C}}_\beta \left( 1+\int _{\{|u_\varepsilon | \ge 1\} \cap B_{3\rho ^{1-\beta }}(x)} W'(u_\varepsilon )^2\right) \int _s^r\rho ^{\gamma -1}d\rho -C. \end{aligned} \end{aligned}$$

### Proof

Substitute (4.6) into the Eq. (4.5) in the proof of Theorem 4.1, we have for $\varepsilon \le s\le \rho \le r\le 1$

5.4 $$\begin{aligned} C(\Lambda _0,q_0)\left( \frac{\mu _\varepsilon (B_r)}{r^n}\right)&\ge \left( \frac{\mu _\varepsilon (B_s)}{s^n}\right) -C(\Lambda _0,q_0)-C\int _s^r\frac{\xi _+(B_\rho )}{\rho ^{n+1}}\nonumber \\&\ge \left( \frac{\mu _\varepsilon (B_s)}{s^n}\right) -C(\Lambda _0,q_0)-C\int _s^r\rho ^{p_3\gamma -n-1}\mu _\varepsilon (B_{2\rho }(x))d\rho \nonumber \\&\quad -C_\beta \varepsilon \int _s^r\rho ^{-M\gamma -n-1}\left( \int _{B_{3\rho ^{1-\beta }}(x)}|f_\varepsilon |^2\right) d\rho \end{aligned}$$

5.5 $$\begin{aligned}&\quad -\int _s^r{\tilde{C}}_\beta \varepsilon \rho ^{\gamma -2}\left( 1+\int _{\{|u_\varepsilon | \ge 1\} \cap B_{3\rho ^{1-\beta }}(x)} W'(u_\varepsilon )^2\right) d\rho . \end{aligned}$$

Noticing $\varepsilon \le \rho$ in the last term, we then conclude the desired energy ratio bound. $\square$

As a corollary, we have

### Corollary 5.4

If in addition to the conditions in Proposition 5.3, we assume

5.6 $$\begin{aligned} \frac{1}{\varepsilon }\int _{B_\rho } f_\varepsilon ^2\le \rho ^{n-1},\quad \text { for } \rho \ge \varepsilon , \end{aligned}$$

and

$$\begin{aligned} \beta \in \left( 0,\frac{1-M\gamma }{2(n-1)}\right) , \end{aligned}$$

then the following upper bound for the energy ratio for $\mu _\varepsilon$ holds

5.7 $$\begin{aligned} \frac{\mu _\varepsilon (B_s(x))}{s^n}\le C\frac{\mu _\varepsilon (B_r(x))}{r^n}+C(\Lambda _0,E_0,q_0,n), \end{aligned}$$

for $$\varepsilon \le s\le r$$.

### Proof

We have

$$\begin{aligned} p_3\gamma -1,-M\gamma +\beta (n-1),\gamma -1>-1. \end{aligned}$$

Thus by Proposition 5.3 and $\varepsilon \le \rho$, we have

$$\begin{aligned} C\left( \frac{\mu _\varepsilon (B_r(x))}{r^n}\right)&\ge \left( \frac{\mu _\varepsilon (B_s(x))}{s^n}\right) -C\int _s^r\rho ^{p_3\gamma -1}\left( \frac{\mu _\varepsilon (B_{2\rho }(x))}{\rho ^n}\right) d\rho \\&\quad -C_\beta \varepsilon ^2\int _s^r\rho ^{-M\gamma -2-\beta (n-1)}\left( \frac{\int _{B_{3\rho ^{1-\beta }}(x)}|f_\varepsilon |^2}{\varepsilon \rho ^{(1-\beta )(n-1)}}\right) d\rho \\&\quad -{\tilde{C}}_\beta \left( 1+\int _{\{|u_\varepsilon | \ge 1\} \cap \Omega } W'(u_\varepsilon )^2\right) \int _s^r\rho ^{\gamma -1}d\rho -C\\&\ge \left( \frac{\mu _\varepsilon (B_s(x))}{s^n}\right) -C\int _s^r\rho ^{p_3\gamma -1}\left( \frac{\mu _\varepsilon (B_{2\rho }(x))}{\rho ^n}\right) d\rho -C. \end{aligned}$$

The conclusion then follows by substituting in (5.6) and applying Gronwall’s inequality to the above differential inequality. $\square$

### Lemma 5.5

For any $N\in {\mathbb {N}}$, $\delta >0$ small, $\Lambda >0$ large and $\beta \in (0,\frac{1-M\gamma }{2(n-1)})$ where $M,\gamma$ are from Proposition 4.2, there exists $\omega >0$ such that the following holds: Suppose $u_\varepsilon$ satisfies (1.1) and the conditions(1)-(3) in Theorem 1.1 are satisfied, then for any finite set $X\subset \{0^n\}\times {\mathbb {R}}\subset {\mathbb {R}}^{n+1}$, and the number of elements in *X* is no more than *N*. If moreover for some $0<\varepsilon \le d\le R\le \omega$, the followings are satisfied

5.8 $$\begin{aligned}&\textrm{diam}(X)<\omega R, \end{aligned}$$

5.9 $$\begin{aligned}&|x-y|>3d, \quad \text { for }x,y\in X\text { and }x\ne y, \end{aligned}$$

5.10 $$\begin{aligned}&|\xi _\varepsilon |(B_\rho (x))+\int _{B_{\rho }(x)}\varepsilon |\nabla u_\varepsilon |^2\sqrt{1-\nu ^2_{\varepsilon ,n+1}}\le \omega \rho ^n,\quad \text { for } x\in X\text { and }d\le \rho \le R, \end{aligned}$$

5.11 $$\begin{aligned}&\frac{1}{\varepsilon }\int _{B_{\rho }(x)}|f_\varepsilon |^2\le \Lambda \rho ^{n-1},\quad \text { for }3d^{1-\beta }\le \rho \le 3R^{1-\beta }. \end{aligned}$$

Then we have

5.12 $$\begin{aligned} \sum _{x\in X}d^{-n}\mu _\varepsilon (B_d(x))\le (1+\delta )R^{-n}\mu _\varepsilon (\cup _{x\in X}B_R(x))+\delta . \end{aligned}$$

The proof of the lemma is based on an inductive application of the sheets-separation proposition, along with appropriate choices of parameters $\gamma$ and $\omega$. To simplify notation in the remainder of this section, we introduce a shorthand for the sheets-separation term

5.13 $$\begin{aligned} {\mathcal {S}}_{y,x}=:(y_{n+1}-x_{n+1})\left( \frac{\varepsilon |\nabla u_\varepsilon |^2}{2}+\frac{W(u_\varepsilon )}{\varepsilon }\right) -\varepsilon \frac{\partial u_\varepsilon }{\partial x_{n+1}}\langle y-x,\nabla u_\varepsilon \rangle , \end{aligned}$$

for any pair of points $x,y\in {\mathbb {R}}^{n+1}$.

### Proposition 5.6

Suppose the conditions in Theorem 1.1 are satisfied and let $X\subset \{0^n\}\times [t_1+d,t_2-d]\subset {\mathbb {R}}^{n+1}$ consist of no more than $N\in {\mathbb {N}}$ elements and $\cup _{x\in X}B_{3R^{1-\beta }}\subset \Omega \subset {\mathbb {R}}^{n+1}$. Furthermore suppose for $-\infty \le t_1<t_2\le \infty , 0<\varepsilon \le d\le R\le \frac{1}{2},\beta \in (0,\frac{1-M\gamma }{2(n-1)})$ the following are satisfied:

5.14 $$\begin{aligned}&(\Gamma +1)\textrm{diam}(X)<R, \quad \text { for some }\Gamma \ge 1, \end{aligned}$$

5.15 $$\begin{aligned}&|x-y|>3d, \quad \text { for }x\ne y\in X, \end{aligned}$$

5.16 $$\begin{aligned}&\int _d^R\rho ^{-n-1}\left| \int _{B_\rho (x)\cap \{y_{n+1}=t_j\}}{\mathcal {S}}_{y,x}d{\mathcal {H}}^n_y\right| d\rho \le \omega \end{aligned}$$

for any $x\in X$, $j=1,2$ and for some $\omega >0$,

5.17 $$\begin{aligned}&|\xi _\varepsilon |(B_\rho (x))+\int _{B_{\rho }(x)}\varepsilon |\nabla u_\varepsilon |^2\sqrt{1-\nu _{\varepsilon ,n+1}^2}\le \omega \rho ^n, \quad \text { for } d\le \rho \le R \end{aligned}$$

5.18 $$\begin{aligned}&\frac{1}{\varepsilon }\int _{B_\rho (x)}|f_\varepsilon |^2\le \Lambda \rho ^{n-1}, \quad \text { for }3d^{1-\beta }\le \rho \le 3R^{1-\beta }, \end{aligned}$$

5.19 $$\begin{aligned}&\frac{\mu _\varepsilon (B_{2R}(x))}{R^n}\le \Lambda , \quad \forall x\in X \quad \text {(this is implied by Corollary}~ 5.4~\text {as}~R\ge \varepsilon \text {)}. \end{aligned}$$

Then by denoting $S_t^{t'}:=\{t\le y_{n+1}\le t'\}$, we have

5.20 $$\begin{aligned} d^{-n}\mu _\varepsilon (B_d(x))\le R^{-n}\mu _\varepsilon (B_R(x)\cap S_{t_1}^{t_2})+ CR^{\gamma _0}+2\omega , \end{aligned}$$

for some $\gamma _0>0$ and for all $x\in X$. Furthermore, if *X* consists of more than one point, then there exists $t_3\in (t_1,t_2)$ such that $\forall x\in X$

5.21 $$\begin{aligned}&|x_{n+1}-t_3|>d, \end{aligned}$$

5.22 $$\begin{aligned}&\int _d^{{\tilde{R}}}\rho ^{-n-1}\int _{B_\rho (x)\cap \{y_{n+1}=t_3\}}\left| {\mathcal {S}}_{y,x}\right| d{\mathcal {H}}^n_yd\rho \le 3N\Gamma \omega , \end{aligned}$$

where ${\tilde{R}}:=\Gamma \textrm{diam}(X)$ and ${\mathcal {S}}_{y,x}$ as defined in (5.13). Moreover, both $X\cap X_{t_1}^{t_3}$ and $X\cap X_{t_3}^{t_2}$ are non-empty and

$$\begin{aligned}&{\tilde{R}}^{-n}\left( \mu _\varepsilon (\cup _{x\in X\cap X_{t_1}^{t_3}}B_{{\tilde{R}}}(x)\cap S_{t_1}^{t_3})+\mu _\varepsilon (\cup _{x\in X\cap X_{t_3}^{t_2}}B_{{\tilde{R}}}(x)\cap S_{t_3}^{t_2})\right) \\&\quad \le \left( 1+\frac{1}{\Gamma }\right) ^nR^{-n}\mu _\varepsilon \left( \cup _{x\in X}B_R(x)\cap S_{t_1}^{t_2}\right) +CR^{\gamma _0}+2\omega . \end{aligned}$$

### Proof

First we choose $\phi$ to be a non-increasing function satisfying

$$\begin{aligned} \phi _{\delta ,\rho }= {\left\{ \begin{array}{ll} 1,&{}\text { on }[0,\rho ]\\ 0,&{}\text { on }[\rho +\delta ,\infty ), \end{array}\right. } \end{aligned}$$

and $$\chi _{\delta }$$ satisfying

$$\begin{aligned}&\chi _\delta \equiv {\left\{ \begin{array}{ll} 1,&{}\text { on }[t_1+\delta , t_2-\delta ],\\ 0,&{}\text { on }(-\infty ,t_1]\cup [t_2,\infty ), \end{array}\right. } \end{aligned}$$

with $\chi _\delta '\ge 0$ on $[t_1,t_1+\delta ]$ and $\chi _\delta '\le 0$ on $[t_2-\delta ,t_2]$. Then we multiply (1.1) on both sides by $\langle \nabla u,\eta \rangle$, where $\eta \in C_0^1(\Omega ,{\mathbb {R}}^{n+1})$ is defined by $\eta (y):=(y-x)\phi _{\delta ,\rho }(|y-x|)\chi _\delta (y_{n+1})$. Using integration by parts, we have

$$\begin{aligned}&\int f_\varepsilon \langle y-x,\nabla u_\varepsilon \rangle \phi _{\delta ,\rho }(|y-x|)\chi _{\delta }(y_{n+1})\\&\quad =\int f_\varepsilon \langle \nabla u,\eta \rangle \\&\quad =\int \left( \frac{\varepsilon |\nabla u_\varepsilon |^2}{2}+\frac{W(u_\varepsilon )}{\varepsilon }\right) \textrm{div}\eta -\varepsilon \nabla u\otimes \nabla u:\nabla \eta \\&\quad =\int \left( |y-x|\phi '_{\delta ,\rho }\chi _{\delta }+(n+1)\phi _{\delta ,\rho }\chi _{\delta }+(y_{n+1}-x_{n+1})\phi _{\delta ,\rho }\chi _{\delta }'\right) d\mu _\varepsilon \\&\qquad -\int \varepsilon \frac{\phi _{\delta ,\rho }'\chi _{\delta }}{|y-x|}\langle y-x,\nabla u_\varepsilon \rangle ^2-\int \varepsilon |\nabla u_\varepsilon |^2\phi _{\delta ,\rho }\chi _{\delta }\\&\qquad -\int \varepsilon \frac{\partial u}{\partial x_{n+1}}\langle y-x,\nabla u_\varepsilon \rangle \phi _{\delta ,\rho }\chi _{\delta }'. \end{aligned}$$

Letting $\delta \rightarrow 0$, we have

$$\begin{aligned}&\int _{B_\rho (x)\cap S_{t_1}^{t_2}}f_\varepsilon \langle y-x,\nabla u_\varepsilon \rangle \\&\quad =-\int _{\partial B_\rho \cap S_{t_1}^{t_2}}\rho d\mu _\varepsilon +(n+1)\int _{B_\rho \cap S_{t_1}^{t_2}}d\mu _\varepsilon \\&\qquad +\int _{B_\rho \cap \{y_{n+1}=t_2\}}(y_{n+1}-x_{n+1})d\mu _\varepsilon -\int _{B_\rho \cap \{y_{n+1}=t_1\}}(y_{n+1}-x_{n+1})d\mu _\varepsilon \\&\qquad +\int _{\partial B_\rho \cap S_{t_1}^{t_2}}\varepsilon \rho ^{-1}\langle y-x,\nabla u_\varepsilon \rangle ^2-\int _{B_\rho \cap S_{t_1}^{t_2}}\varepsilon |\nabla u_\varepsilon |^2\\&\qquad +\int _{B_\rho \cap \{y_{n+1}=t_2\}}\varepsilon \frac{\partial u}{\partial x_{n+1}}\langle y-x,\nabla u_\varepsilon \rangle -\int _{B_\rho \cap \{y_{n+1}=t_1\}}\varepsilon \frac{\partial u}{\partial x_{n+1}}\langle y-x,\nabla u_\varepsilon \rangle . \end{aligned}$$

Dividing both sides by $\rho ^{n+1}$ and rearranging gives the following weighted monotonicity formula

5.23 $$\begin{aligned}&\frac{d}{d\rho }\left( \rho ^{-n}\mu _\varepsilon (B_\rho (x)\cap S_{t_1}^{t_2})\right) \nonumber \\&\quad =-n\rho ^{-n-1}\mu _\varepsilon (B_\rho (x)\cap S_{t_1}^{t_2})+\rho ^{-n}\mu _\varepsilon (\partial B_\rho \cap S_{t_1}^{t_2})\nonumber \\&\quad =-(n+1)\rho ^{-n-1}\int _{B_\rho (x)\cap S_{t_1}^{t_2}}d\mu _\varepsilon +\rho ^{-n}\int _{\partial B_\rho (x)\cap S_{t_1}^{t_2}}d\mu _\varepsilon \nonumber \\&\qquad +\rho ^{-n-1}\int _{B_\rho (x)\cap S_{t_1}^{t_2}}\varepsilon |\nabla u_\varepsilon |^2-\rho ^{-n-1}\int _{B_\rho (x)\cap S_{t_1}^{t_2}}d\xi _\varepsilon \nonumber \\&\quad =\rho ^{-n-1}\int _{B_\rho \cap \{y_{n+1}=t_2\}}(y_{n+1}-x_{n+1})d\mu _\varepsilon \nonumber \\&\qquad -\rho ^{-n-1}\int _{B_\rho \cap \{y_{n+1}=t_1\}}(y_{n+1}-x_{n+1})d\mu _\varepsilon \nonumber \\&\qquad +\rho ^{-n-1}\int _{B_\rho \cap \{y_{n+1}=t_2\}}\varepsilon \frac{\partial u}{\partial x_{n+1}}\langle y-x,\nabla u_\varepsilon \rangle \nonumber \\&\qquad -\rho ^{-n-1}\int _{B_\rho \cap \{y_{n+1}=t_1\}}\varepsilon \frac{\partial u}{\partial x_{n+1}}\langle y-x,\nabla u_\varepsilon \rangle \nonumber \\&\qquad -\rho ^{-n-1}\int _{B_\rho (x)\cap S_{t_1}^{t_2}}d\xi _\varepsilon -\rho ^{-n-1}\int _{B_\rho (x)\cap S_{t_1}^{t_2}}f_\varepsilon \langle y-x,\nabla u_\varepsilon \rangle \nonumber \\&\qquad +\rho ^{-n-1}\int _{\partial B_\rho \cap S_{t_1}^{t_2}}\varepsilon \rho ^{-1}\langle y-x,\nabla u_\varepsilon \rangle ^2. \end{aligned}$$

By the condition given by (5.16), the sum of norms of the first fours terms are bounded by $2\omega$. And by (4.6) and (5.18), the discrepancy term is bounded by

$$\begin{aligned}&\rho ^{-n-1}\int _{B_\rho (x)\cap S_{t_1}^{t_2}}d\xi _{\varepsilon ,+}\\&\quad \le C\rho ^{p_3\gamma -n-1}\mu _\varepsilon (B_{2\rho }(x))+{\tilde{C}}_k\varepsilon \rho ^{-M\gamma -n-1}\int _{B_{3\rho ^{1-\beta }}(x)}|f_\varepsilon |^2\\&\qquad +{\tilde{C}}_\beta \varepsilon \rho ^{\gamma -2}\left( 1+\int _{\{|u_\varepsilon | \ge 1\} \cap \Omega } W'(u_\varepsilon )^2\right) \\&\quad \le C\rho ^{p_3\gamma -1}\left( \frac{\mu _\varepsilon (B_{2\rho }(x)\cap S_{t_1}^{t_2})}{\rho ^n}\right) +C\varepsilon \rho ^{-M\gamma -n-1}\Lambda \varepsilon \rho ^{(1-\beta )(n-1)}+C\varepsilon \rho ^{\gamma -2}\\&\quad \le C\rho ^{p_3\gamma -1}\left( \frac{\mu _\varepsilon (B_{2\rho }(x)\cap S_{t_1}^{t_2})}{\rho ^n}\right) +C\rho ^{2-M\gamma -n-1+(n-1)-\beta (n-1)}+C\rho ^{\gamma -1}\\&\quad \le C\rho ^{p_3\gamma -1}+C\rho ^{-1+\frac{1-M\gamma }{2}}+C\rho ^{\gamma -1}, \end{aligned}$$

where we used (5.7) and $\varepsilon \le \rho$ in the last line. By (4.3) in the proof of Theorem 4.1 and (5.7), we have

$$\begin{aligned} \rho ^{-n-1}\left| \int _{B_\rho (x)\cap S_{t_1}^{t_2}}f_\varepsilon \langle y-x,\nabla u_\varepsilon \rangle \right| \le C\rho ^{-\frac{n}{q_0}}\left( 1+\frac{\mu _\varepsilon (B_{\rho }\cap S_{t_1}^{t_2})}{\rho ^n}\right) \le {\tilde{C}}\rho ^{-\frac{n}{q_0}}. \end{aligned}$$

By integrating (5.23) from *d* to *R* and noting $B_d(x)\subset S_{t_1}^{t_2}$, we obtain the following upper bound of energy density for $\mu _\varepsilon$,

$$\begin{aligned} d^{-n}\mu _\varepsilon (B_d(x))=d^{-n}\mu _\varepsilon (B_d(x)\cap S_{t_1}^{t_2})\le R^{-n}\mu _\varepsilon (B_R(x)\cap S_{t_1}^{t_2})+ CR^{\gamma _0}+2\omega , \end{aligned}$$

where $\gamma _0=\min \{\frac{q_0-n}{q_0},p_3\gamma ,\frac{1-M\gamma }{2},\gamma \}>0$. This proves (5.20).

Next, if *X* contains more than one point, then we can choose $x_\pm \in X$ such that $x_{+,n+1}-x_{-,n+1}>\frac{\textrm{diam} X}{N}$ (where $x_{\pm ,n+1}$ denotes the $(n+1)$-th coordinate of $x_\pm$) and there is no other element of *X* in $\{0\}\times (x_{-,n+1},x_{+,n+1})$. Let ${\tilde{t}}_1:=x_{-,n+1}+\frac{x_{+,n+1}-x_{-,n+1}}{3}$ and ${\tilde{t}}_2:=x_{+,n+1}-\frac{x_{+,n+1}-x_{-,n+1}}{3}$. For $x\in X, y\in B_\rho (x), d\le \rho \le {\tilde{R}}$, we have

$$\begin{aligned} |{\mathcal {S}}_{y,x}|&=\left| (y_{n+1}-x_{n+1})\left( \frac{\varepsilon |\nabla u_\varepsilon |^2}{2}+\frac{W(u_\varepsilon )}{\varepsilon }\right) -\varepsilon \frac{\partial u_\varepsilon }{\partial x_{n+1}}\langle y-x,\nabla u_\varepsilon \rangle \right| \\&=\left| (y_{n+1}-x_{n+1})\left( \frac{W(u_\varepsilon )}{\varepsilon }-\frac{\varepsilon |\nabla u_\varepsilon |^2}{2}\right) +|(y_{n+1}-x_{n+1})\varepsilon |\nabla u_\varepsilon |^2\right. \\&\quad \left. -\varepsilon \frac{\partial u_\varepsilon }{\partial x_{n+1}}\langle y-x,\nabla u_\varepsilon \rangle \right| \\&\le \rho \left| \frac{\varepsilon |\nabla u_\varepsilon |^2}{2}-\frac{W(u_\varepsilon )}{\varepsilon }\right| +\varepsilon |\nabla u_\varepsilon |^2|\langle y-x,e_{n+1}\rangle -\langle y-x,\nu _\varepsilon \rangle \langle e_{n+1},\nu _\varepsilon \rangle |\\&\le \rho \left| \frac{\varepsilon |\nabla u_\varepsilon |^2}{2}-\frac{W(u_\varepsilon )}{\varepsilon }\right| +\varepsilon |\nabla u_\varepsilon |^2|y-x|\sqrt{1-\nu _{\varepsilon ,n+1}^2}\\&\le \rho \left| \frac{\varepsilon |\nabla u_\varepsilon |^2}{2}-\frac{W(u_\varepsilon )}{\varepsilon }\right| +\rho \varepsilon |\nabla u_\varepsilon |^2\sqrt{1-\nu _{\varepsilon ,n+1}^2}. \end{aligned}$$

And thus by condition (5.17), we have

$$\begin{aligned}&\int _{{\tilde{t}}_1}^{{\tilde{t}}_2}\int _d^{{\tilde{R}}}\rho ^{-n-1}\int _{B_\rho (x)\cap \{y_{n+1}=t\}}\left| {\mathcal {S}}_{y,x}\right| d{\mathcal {H}}^n_{\{y_{n+1}=t\}}d\rho dt\\&\quad =\int _d^{{\tilde{R}}}\rho ^{-n-1}\int _{B_\rho (x)\cap S_{{\tilde{t}}_1}^{{\tilde{t}}_2}}\left| (y_{n+1}-x_{n+1})\left( \frac{\varepsilon |\nabla u_\varepsilon |^2}{2}+\frac{W(u_\varepsilon )}{\varepsilon }\right) \right. \\&\qquad \left. -\varepsilon \frac{\partial u_\varepsilon }{\partial x_{n+1}}\langle y-x,\nabla u_\varepsilon \rangle \right| dyd\rho \\&\quad \le \int _d^{{\tilde{R}}}\rho ^{-n}\int _{B_\rho (x)\cap S_{{\tilde{t}}_1}^{{\tilde{t}}_2}}\left| \frac{\varepsilon |\nabla u_\varepsilon |^2}{2}-\frac{W(u_\varepsilon )}{\varepsilon }\right| +\varepsilon |\nabla u_\varepsilon |^2\sqrt{1-\nu _{\varepsilon ,n+1}^2}dyd\rho \\&\quad \le \int _d^{{\tilde{R}}}\rho ^{-n}\omega \rho ^nd\rho \\&\quad \le \omega {\tilde{R}}. \end{aligned}$$

So there must exist a $t_3\in [{\tilde{t}}_1,{\tilde{t}}_2]$ such that

$$\begin{aligned}&\int _d^{{\tilde{R}}}\rho ^{-n-1}\int _{B_\rho (x)\cap \{y_{n+1}=t_3\}}\left| {\mathcal {S}}_{y,x}\right| d{\mathcal {H}}^n_{\{y_{n+1}=t\}}d\rho \\&\quad \le \frac{\omega {\tilde{R}}}{({\tilde{t}}_2-{\tilde{t}}_1)}\le \frac{3N\omega {\tilde{R}}}{\textrm{diam}(X)}=3N\Gamma \omega . \end{aligned}$$

By the choice of $t_3\in [{\tilde{t}}_1,{\tilde{t}}_2]$, we automatically have $|x_{n+1}-t_3|>d$ for all $x\in X$. Finally, by denoting

$$\begin{aligned} X_+:=\{x\in X,x_n\ge t_3\}, X_-:=\{x\in X,x_n< t_3\}, \end{aligned}$$

we have $X_\pm \ne \emptyset$ and

$$\begin{aligned} \left( \cup _{x\in X_-}B_{{\tilde{R}}}(x)\cap S_{t_1}^{t_3}\right) \cup \left( \cup _{x\in X_+}B_{{\tilde{R}}}(x)\cap S_{t_3}^{t_2}\right) \subset B_{{\tilde{R}}+\textrm{diam}(X)}(x_0)\cap S_{t_1}^{t_2}, \end{aligned}$$

for any $x_0\in X$. By (5.20)(with ${\tilde{R}}+\textrm{diam}(X)$ in place of *d*), we then have

$$\begin{aligned}&{\tilde{R}}^{-n}\left( \mu _\varepsilon \left( \cup _{x\in X_-}B_{{\tilde{R}}}(x)\cap S_{t_1}^{t_3}\right) +\mu _\varepsilon \left( \cup _{x\in X_+}B_{{\tilde{R}}}(x)\cap S_{t_3}^{t_2}\right) \right) \\&\quad \le {\tilde{R}}^{-n}\mu _\varepsilon (B_{{\tilde{R}}+\textrm{diam}(X)}(x_0)\cap S_{t_1}^{t_2})\\&\quad =\left( 1+\frac{1}{\Gamma }\right) ^n({\tilde{R}}+\textrm{diam}(X))^{-n}\mu _\varepsilon (B_{{\tilde{R}}+\textrm{diam}(X)}(x_0)\cap S_{t_1}^{t_2})\\&\quad \le \left( 1+\frac{1}{\Gamma }\right) ^n\left( R^{-n}\mu _\varepsilon (B_R(x_0)\cap S_{t_1}^{t_2})+CR^{\gamma _0}+2\omega \right) \\&\quad \le \left( 1+\frac{1}{\Gamma }\right) ^n\left( R^{-n}\mu _\varepsilon (\cup _{x\in X}B_R(x)\cap S_{t_1}^{t_2})\right) +CR^{\gamma _0}+2\omega . \end{aligned}$$

$$\square $$

The next Lemma taken from [8] shows the energy ratio at small scales are very close to the 1-d solution.

### Lemma 5.7

(Lemma 5.5 of [8]) Suppose the conditions in Theorem 1.1 are satisfied. For any $\tau \in (0,1$),$\delta >0$ small, $\Lambda >0$ large, there exists $\omega >0$ sufficiently small and $L>1$ sufficiently large such that the following holds: Suppose $u_\varepsilon$ satisfies condition of Theorem 1.1 in $B_{4L\varepsilon }(0)\subset {\mathbb {R}}^{n+1}$ and

5.24 $$\begin{aligned}&|u_\varepsilon (0)|\le 1-\tau \end{aligned}$$

5.25 $$\begin{aligned}&|\xi _\varepsilon (B_{4L\varepsilon }(0))|+\int _{B_{4L\varepsilon }(0)}\varepsilon |\nabla u_\varepsilon |^2\sqrt{1-\nu _{\varepsilon ,n+1}^2}\le \omega (4L\varepsilon )^n \end{aligned}$$

5.26 $$\begin{aligned}&\frac{1}{\varepsilon }\int _{B_{4L\varepsilon }(0)}|f_\varepsilon |^2\le \Lambda (4L\varepsilon )^{n-2} \end{aligned}$$

5.27 $$\begin{aligned}&\mu _\varepsilon ({B_{4L\varepsilon }(0)})\le \Lambda (4L\varepsilon )^n. \end{aligned}$$

Then by denoting $(0,t)\in {\mathbb {R}}^{n+1}$ to be the point with first *n*-th coordinate functions being 0 and the $(n+1)$-th coordinate functions being *t*, we have

5.28 $$\begin{aligned}&|u(0,t)|\ge 1-\frac{\tau }{2},\quad \text { for all }L\varepsilon \le |t|\le 3L\varepsilon \end{aligned}$$

5.29 $$\begin{aligned}&\left| \frac{1}{\omega _n(L\varepsilon )^n}\mu _\varepsilon (B_{L\varepsilon }(0))-\alpha \right| \le \delta \end{aligned}$$

5.30 $$\begin{aligned}&\left| \int _{-L\varepsilon }^{L\varepsilon }W(u_\varepsilon (0,t))dt-\frac{\alpha }{2}\right| \le \delta . \end{aligned}$$

### Proof

First we consider the 1-dimensional solution

$$\begin{aligned} q_0'(t)&= \sqrt{W(q_0(t))}\quad \forall t \in {\mathbb {R}}, \\ q_0(0)&= u(0). \end{aligned}$$

We will use $$q_0$$ to choose *L* depending on $$ \tau , \delta > 0$$. On $$ {\mathbb {R}}^{n+1}$$ we write $$q(x) = q_0(x_{n+1})$$ and choose $$ L >1$$ large enough depending on $$ \tau , \delta $$ so that

5.31 $$\begin{aligned} \begin{aligned}&|q(0,t)|\ge 1 - \frac{\tau }{3}, \quad \text { for all } L \le |t| \le 3 L,\\&\left| \frac{1}{\omega _n L^{n-1}}\int _{B_L(0)} \left( \frac{|\nabla q|^2}{2} + W(q) \right) - \alpha \right| \le \frac{\delta }{2}\\&\left| \int _{-L}^L W(q(0,t)) dt - \frac{\alpha }{2} \right| \le \frac{\delta }{2} \end{aligned} \end{aligned}$$

whenever $|q(0)| \le 1 - \tau$. The function *u* satisfies the Allen–Cahn equation

$$\begin{aligned} -\Delta u + W'(u) = f , \end{aligned}$$

and by our condition (2) in Theorem 1.1 we get $\Vert u_\varepsilon \Vert _{L^\infty (B_{1/2}(x))}\le c_0$. Hence by Calderon–Zygmund estimates we get uniform $W^{2,\frac{n+1}{2} + \delta _0}$ estimates on $B_{3\,L}(0)$ of the form

5.32 $$\begin{aligned} \Vert u\Vert _{W^{2,\frac{n+1}{2} + \delta _0}(B_{3L}(0))} \le C(\Lambda ,L). \end{aligned}$$

If there is no such $\omega >0$ such that (5.28), (5.29) and (5.30) holds then this implies there exists $\omega _j\rightarrow 0$ and $u_j, f_j$ satisfying the above estimates but that do not satisfy (5.28), (5.29) and (5.30). By (5.32), we get after passing to a suitable subsequence that $u_j \rightharpoonup u$ weakly in $W^{2,\frac{n+1}{2} + \delta _0}(B_{3\,L}(0))$ and $f_j \rightharpoonup f$ weakly in $L^{\frac{n+1}{2} + \delta _0}(B_{3\,L}(0))$. By the Sobolev embedding we have $W^{2,\frac{n+1}{2} + \delta _0}(B_{3L}(0)) \hookrightarrow C^0$ for $\delta _0>0$ and hence we get $u_j\rightarrow u$ uniformly in $C^0(B_{3L}(0))$. $\square$

### Claim

The functions $u_j\rightarrow u = q$ strongly in $W^{1,2}(B_{3L}(0))$.

### Proof

Writing $\nabla =(\nabla ', \partial _{n+1})$ we get (5.25)

$$\begin{aligned} \int _{B_{3L(0)}}\left| \frac{|\nabla u|^2}{2} - W(u)\right|&\le \liminf _{j\rightarrow \infty } \int _{B_{3L}(0)} \left| \frac{|\nabla u_j|^2}{2} - W(u_j)\right| \\&\le \liminf _{j\rightarrow \infty }|\xi _j |(B_{3L}(0)) = 0 \end{aligned}$$

and

$$\begin{aligned} \int _{B_{3L}(0)} |\nabla ' u |&\le \liminf _{j\rightarrow \infty } \int _{B_{3L}(0)} |\nabla ' u_j | \le C(L) \left( \int _{B_{3L}(0)} |\nabla u_j|^2 \sqrt{1 - \nu _{j,n}^2} \right) ^{1/2} =0, \end{aligned}$$

where $$ \nu _j = \frac{\nabla u_j}{|\nabla u|}$$ for $$ \nabla u_j \ne 0$$. Therefore $$ |\nabla u|^2 = 2 W(u)$$ and $$ u(y,t) = u_0(t)$$ for some $$ u_0 \in W^{2,\frac{n+1}{2} + \delta _0}((-L,L))\hookrightarrow C^{1,\alpha }((-L,L))$$ and $$ | u'_0| = 2 \sqrt{2W(u_0)}$$. As $$ |u_0(0)| \le 1 - \tau $$ by uniform convergence, we see $$ |u_0| < 1$$ and $$ |u'_0| > 0$$. After a reflection of the form $$(y,x_n)\mapsto (y,-x_n)$$ if necessary, we may assume $$u_0'>0$$ and hence $$ u_0'= \sqrt{2W(u_0)}$$. This gives us $$ u_0 = q_0$$ and $$ u = q$$. This shows $$ u_j\rightarrow u = q $$ strongly in $$W^{1,2}(B_{3L}(0))$$. $$\square $$

From this claim and (5.31) we conclude $u_j$ satisfies (5.28), (5.29) and (5.30) for sufficiently large *j* which is a contradiction. $\square$

Now we prove Proposition 5.2.

### Proof of Proposition 5.2

Without loss of generality, we assume $P_0=\{x\in {\mathbb {R}}^{n+1},x_{n+1}=0\}$ and let $\pi :{\mathbb {R}}^{n+1}\rightarrow P_0$ denote the associated orthogonal projection. Furthermore we know

$$\begin{aligned} V_\varepsilon = \mu _\varepsilon \otimes \nu _\varepsilon \rightarrow V \end{aligned}$$

is rectifiable and

$$\begin{aligned} \mu _V&= \mu \\ V&= \theta _0 {\mathcal {H}}^n \llcorner P_0 \otimes \delta _{P_0} \end{aligned}$$

and

5.33 $$\begin{aligned} \lim _{\varepsilon \rightarrow 0}\int _{B_4(0)} \varepsilon |\nabla u_\varepsilon |^2 \sqrt{1 - \nu _{\varepsilon , n+1}^2} = 0. \end{aligned}$$

Let $N\in {\mathbb {N}}$ be the smallest integer with

$$\begin{aligned} N > \frac{\theta _0}{\alpha } \end{aligned}$$

and let $0 < \delta \le 1$ be small. By Proposition 3.5 and the $L^\infty$ bound condition of $u_\varepsilon$ in Theorem 1.1, we can fix $\tau >0$ such that $\forall \varepsilon (\delta )>0$ sufficiently small we have

$$\begin{aligned} \int _{\{|u_\varepsilon |\ge 1 -\tau \} \cap B_4(0)}\frac{W'^2(u_\varepsilon )}{\varepsilon }+\frac{W(u_\varepsilon )}{\varepsilon }\le \delta . \end{aligned}$$

We have by Lemma 3.8,

5.34 $$\begin{aligned} \mu _\varepsilon (\{|u_\varepsilon |\ge 1 -\tau \cap B_4(0)\})&\le | \xi _\varepsilon (B_4(0))|\nonumber \\&\quad +2\int _{\{|u_\varepsilon |\ge 1 -\tau \} \cap B_4(0)}\frac{W(u_\varepsilon )}{\varepsilon } \le 3 \delta . \end{aligned}$$

We want to apply Lemma 5.5 and Lemma 5.7. We choose $0 < \omega =\omega (N,\delta , \frac{1}{2},\frac{1}{2}, C)$ and $\omega (\delta , \tau , C) \le 1$ where $L = L(\delta , \tau )$ which are the parameters that appear in Lemma 5.5 and Proposition 5.6 and *C* is the constant so that

$$\begin{aligned} \mu _\varepsilon (\Omega ) + \frac{1}{\varepsilon }\int _{\Omega }|f_\varepsilon |^2 \le C \quad \Omega = B_4(0). \end{aligned}$$

We define $A_\varepsilon$ to be the set where the hypotheses for our Propositions hold, that is

$$\begin{aligned} A_\varepsilon = \left\{ x \in B_1(0) \left| \begin{array}{l} |u_\varepsilon (x)|\le 1 - \tau , \\ \forall \varepsilon \le \rho \le 3 : |\xi _\varepsilon (B_{\rho }(x))|+\int _{B_{\rho }(x)}\varepsilon |\nabla u_\varepsilon |^2\sqrt{1-\nu _{\varepsilon ,n+1}^2} \le \omega \rho ^n, \\ \forall \varepsilon \le \rho \le 3 : \frac{1}{\varepsilon }\int _{B_\rho (x)}|f_\varepsilon |^2 \le \omega \rho ^{n-1}. \end{array} \right. \right\} . \end{aligned}$$

We show the complement of the set $A_\varepsilon$ has small measure. By Besicovitch’s covering theorem, we find a countable sub-covering $\cup _iB_{\rho _i}(x_i),\rho _i\in [\varepsilon ,3]$ of $\{|u_\varepsilon |\le 1-\tau \}\setminus A_\varepsilon$ such that every point $x\in \{|u_\varepsilon |\le 1-\tau \}\setminus A_\varepsilon$ belongs to at most ${\textbf{B}}_n$ balls in the covering, where ${\textbf{B}}_n$ depends only on the dimension *n*. For each *i*, either

$$\begin{aligned} |\xi _\varepsilon (B_{\rho _i}(x_i))|+\int _{B_{\rho _i}(x_i)}\varepsilon |\nabla u_\varepsilon |^2\sqrt{1-\nu _{\varepsilon ,n+1}^2} \ge \omega \rho _i^n, \end{aligned}$$

or

$$\begin{aligned} \frac{1}{\varepsilon }\int _{B_{\rho _i}(x_i)}|f_\varepsilon |^2 \ge \omega \rho _i^{n-1}\ge C\omega \rho _i^n. \end{aligned}$$

On the other hand, by (5.2), for sufficiently small $\varepsilon$, we have

$$\begin{aligned} \frac{1}{\varepsilon }\int _{B_{\rho }(x_i)}|f_\varepsilon |^2 \le \omega \rho ^{n-1}, \forall \rho \in [\varepsilon ,3]. \end{aligned}$$

By (5.7), for each *i*, we obtain

$$\begin{aligned} \mu _\varepsilon \left( \overline{B_{\rho _i}(x_i)}\right) \le C\rho _i^n. \end{aligned}$$

Since the overlap in the Besicovitch covering is finite and (5.34), we get

5.35 $$\begin{aligned} \begin{aligned} \mu _\varepsilon \left( B_1(0)\setminus A_\varepsilon \right)&\le 3\delta +\sum _iC\rho _i^n\\&\le 3\delta +C\omega ^{-1}\left( |\xi _\varepsilon |(B_4(0))+\int _{B_4(0)}\varepsilon |\nabla u_\varepsilon |^2\sqrt{1-\nu _{\varepsilon ,n+1}^2}\right. \\&\quad \left. +\frac{1}{\varepsilon }\int _{B_4(0)}|f_\varepsilon |^2\right) \\&\le 4\delta , \end{aligned}\nonumber \\ \end{aligned}$$

for $\varepsilon$ sufficiently small. First by Lemma 5.5 and Lemma 5.7 we have $x\in A_\varepsilon , \forall L\varepsilon \le R\le \omega$,

$$\begin{aligned} \alpha \omega _n-\delta \le (1+\delta )R^{-n}\mu _\varepsilon (B_R(x))+\delta . \end{aligned}$$

By the reduction to the conditions in Proposition 5.2, we obtain

$$\begin{aligned} \mu _\varepsilon \left( \Omega \setminus \{|x_{n+1}|\le \zeta \}\right) \rightarrow 0, \quad \text { for any fixed }\zeta >0. \end{aligned}$$

Thus, for sufficiently small $\delta >0$, we get

$$\begin{aligned} A_\varepsilon \subset \{|x_{n+1}|\le \zeta _\varepsilon \},\quad \text { with } \zeta _\varepsilon \rightarrow 0\text { as }\varepsilon \rightarrow 0. \end{aligned}$$

For any ${\hat{y}}\in B^n_1(0)\subset {\mathbb {R}}^n$, consider a maximal subset

$$\begin{aligned} X=\{y\}\times \{t_1<...<t_K\}\subset A_\varepsilon \cap \pi ^{-1}(y) \end{aligned}$$

with $|x-x'|>3\,L\varepsilon$ if $x\ne x' \in X$, where $\pi$ denotes the projection to $\{x_{n+1}=0\}$. If $K\ge N$, we apply Lemma 5.5 with $d=3L\varepsilon , R=\omega$ and Lemma 5.7 to get

$$\begin{aligned} N\alpha \omega _n-N\delta \le (1+\delta )R^{-n}\mu _\varepsilon \left( B_R(y)\right) +\delta \le (1+\delta )R^{-n}\mu _\varepsilon \left( B_{R+\zeta _\varepsilon }(y)\right) +\delta . \end{aligned}$$

As

$$\begin{aligned} \limsup _{\varepsilon \rightarrow 0}(1+\delta )R^{-n}\mu _\varepsilon \left( B_{R+\zeta _\varepsilon }(y)\right) \le R^{-n}\mu (\overline{B_R(y)})+C\delta =\theta \omega _n+C\delta , \end{aligned}$$

and $\delta >0$ is arbitrarily small, we have

$$\begin{aligned} N\alpha \le \theta , \end{aligned}$$

which is a contradiction to our definition of *N*. So we obtain

$$\begin{aligned} K\le N-1. \end{aligned}$$

Since *X* is maximal, we get

$$\begin{aligned} A_\varepsilon \cap \pi ^{-1}(y)\subset \{y\}\times \cup _{k=1}^K(t_k-3L\varepsilon ,t_k+3L\varepsilon ). \end{aligned}$$

By (5.28),

$$\begin{aligned} A_\varepsilon \cap \pi ^{-1}(y)\cap&\left( \{y\}\times \cup _{k=1}^K(t_k-3L\varepsilon ,t_k+3L\varepsilon )\right) \\&=A_\varepsilon \cap \pi ^{-1}(y)\cap \left( \{y\}\times \cup _{k=1}^K(t_k-L\varepsilon ,t_k+L\varepsilon )\right) . \end{aligned}$$

So

$$\begin{aligned} A_\varepsilon \cap \pi ^{-1}(y)\subset \{y\}\times \cup _{k=1}^K(t_k-L\varepsilon ,t_k+L\varepsilon ) \end{aligned}$$

and by (5.30),

$$\begin{aligned} \int _{t_k-L\varepsilon }^{t_k+L\varepsilon }\frac{W(u_\varepsilon (y,t))}{\varepsilon }dt\le \frac{\alpha }{2}+\delta , \quad \forall k=1,...,K. \end{aligned}$$

Hence summing over *k* gives

$$\begin{aligned} \int _{A_\varepsilon \cap \pi ^{-1}(y)}\frac{W(u_\varepsilon )}{\varepsilon }d{\mathcal {H}}^1\le \frac{(N-1)\alpha }{2}+(N-1)\delta \end{aligned}$$

and integrating over $B^n_1(0)\subset {\mathbb {R}}^n$ we obtain

$$\begin{aligned} \int _{B_1^{n+1}(0)\cap A_\varepsilon }\frac{1}{\varepsilon }W(u_\varepsilon )d{\mathcal {H}}^{n+1}&\le \int _{B_1^n(0)}\int _{A_\varepsilon \cap \pi ^{-1}(y)}\frac{W(u_\varepsilon )}{\varepsilon }d{\mathcal {H}}^1dy\\ {}&\quad \le \frac{(N-1)\alpha \omega }{2}+C\delta . \end{aligned}$$

Recalling (5.35), we get

$$\begin{aligned} \mu _\varepsilon \left( B_1(0)\right)&\le \int _{B_1^{n+1}(0)\cap A_\varepsilon }\frac{1}{\varepsilon }W(u_\varepsilon )d{\mathcal {H}}^{n+1}+|\xi _\varepsilon \left( B_1(0)\right) |+\mu _\varepsilon \left( B_1(0)\setminus A_\varepsilon \right) \\&\le (N-1)\alpha \omega _n+C\delta . \end{aligned}$$

On the other hand, since $\lim _{\varepsilon \rightarrow 0}\mu _\varepsilon \left( B_1(0)\right) =\theta \omega _n$ and $\delta >0$ is arbitrarily small, we obtain

$$\begin{aligned} \theta \le (N-1)\alpha . \end{aligned}$$

And since by definition *N* is the smallest integer such that $\theta <N\alpha$, we have

$$\begin{aligned} \theta =(N-1)\alpha . \end{aligned}$$

$$\square $$

## Proof of corollaries and applications of Theorem 1.1

In this section, we provide the proof of Corollary 1.3 and Corollary 1.2, which are applications of Theorem 1.1.

We first prove the convergence result under various other Sobolev conditions on the inhomogeneous term.

### Proof of Corollary 1.3

1. To see the first condition implies the conditions in Theorem 1.1, we choose $q_0=\frac{t(s-2)}{s}+2$ ($q_0>n$ is satisfied due to the choice of *t* and *s* above). Then we have
  $$\begin{aligned} \int _\Omega \left| \frac{f_\varepsilon }{\varepsilon |\nabla u_\varepsilon |}\right| ^{q_0}\varepsilon |\nabla u_\varepsilon |^2dx&=\int _\Omega \left| \frac{f_\varepsilon }{\varepsilon |\nabla u_\varepsilon |}\right| ^{q_0-2}\frac{|f_\varepsilon |^2}{\varepsilon }dx\\&\le \frac{1}{\varepsilon }\left( \int _\Omega \left| \frac{f_\varepsilon }{\varepsilon |\nabla u_\varepsilon |}\right| ^\frac{(q_0-2)s}{s-2}\right) ^\frac{s-2}{s}\left( \int _\Omega f_\varepsilon ^{2\frac{s}{2}}\right) ^\frac{2}{s}\\&=\frac{1}{\varepsilon }\cdot \left\| \frac{f_\varepsilon }{\varepsilon |\nabla u_\varepsilon |}\right\| _{L^t(\Omega )}^{q_0-2}\cdot \Vert f_\varepsilon \Vert _{L^s(\Omega )}^2\\&\le C_1^2C_2^{q_0-2}\le \Lambda _0 \end{aligned}$$
  where we used Hölder’s inequality in the second line with exponent $\frac{s}{s-2}$.
2. In the paper [11], assuming condition (2) above, the authors proved the same integer rectifiability and $L^{q_0}$ mean curvature bound for the limit varifold. We show this conditions implies the integral bounds in the hypothesis of Theorem 1.1 for some $q_0>n$. To see this, we compute
  $$\begin{aligned} \nabla \left( \phi ^{\frac{np}{n+1-p}} \right) = \frac{np}{n+1-p} \phi ^{\frac{(n+1)(p-1)}{n+1-p}} \nabla \phi . \end{aligned}$$
  and applying [12, 5.12.4](c.f. [11, Theorem 3.7]) and [11, Theorem 3.8], and Hölder’s inequality, with $\varphi = \phi ^{\frac{np}{n+1-p}}$ and $d \mu = \varepsilon |\nabla u_\varepsilon |^2 d{\mathcal {L}}^{n+1}$.
  $$\begin{aligned} \left| \int _{\mathbb {R}^n}\varphi d \mu \right| \le c(n) K(\mu ) \int _{\mathbb {R}^n}|\nabla \varphi | d \mathcal {L}^n \quad \forall \varphi \in C_c^1\left( \mathbb {R}^{n+1}\right) \end{aligned}$$
  which implies
  $$\begin{aligned} \left| \int _{{\mathbb {R}}^{n+1}} |\phi |^{\frac{np}{n+1-p}} \varepsilon |\nabla u_\varepsilon |^2 d {\mathcal {L}}^{n+1} \right|&\le \left| \int _{{\mathbb {R}}^{n+1}}\varphi d \mu \right| \\&\le C(n)K(\mu )\left| \int _{{\mathbb {R}}^{n+1}} \frac{np}{n+1-p} |\nabla \phi | |\phi |^{\frac{(n+1)(p-1)}{n+1-p}}d {\mathcal {L}}^{n+1}\right| \\&\le C(n,p)K(\mu )\left| \int _{{\mathbb {R}}^{n+1}} |\nabla \phi | |\phi |^{\frac{(n+1)(p-1)}{n+1-p}} d {\mathcal {L}}^{n+1}\right| \\&\le C(n,p) \left( \int _{{\mathbb {R}}^{n+1}}|\nabla \phi |^p \right) ^{1/p} \left( \int _{{\mathbb {R}}^{n+1}}|\phi |^{\frac{p(n+1)}{n+1-p}} \right) ^\frac{p-1}{p}\\&= C(n,p) \Vert \nabla \phi \Vert _{L^p({\mathbb {R}}^{n+1})} \Vert \phi \Vert _{L^{\frac{p(n+1)}{n+1-p}}({\mathbb {R}}^{n+1})}^{\frac{(p-1)(n+1)}{n+1-p}}. \end{aligned}$$
  where $C(n,p)\rightarrow \infty$ as $p\rightarrow n+1$. We apply the above inequality with $\phi =\psi \frac{f_\varepsilon }{\varepsilon |\nabla u_\varepsilon |}$ and $d \mu = \varepsilon |\nabla u_\varepsilon |^2$ together the Sobolev inequality to get for $\psi \in C_0^1(\Omega )$
  $$\begin{aligned}&\int _\Omega \left| \psi \frac{f_\varepsilon }{\varepsilon |\nabla u_\varepsilon |}\right| ^{\frac{pn}{n+1-p}}\varepsilon |\nabla u_\varepsilon |^2 d {\mathcal {L}}^{n+1}\\&\quad \le C\left\| \nabla \left( \psi \frac{f_\varepsilon }{\varepsilon |\nabla u_\varepsilon |}\right) \right\| _{L^p(\Omega )}\left\| \psi \frac{f_\varepsilon }{\varepsilon |\nabla u_\varepsilon |}\right\| _{L^\frac{p(n+1)}{n+1-p}(\Omega )}^\frac{(p-1)(n+1)}{n+1-p}\\&\quad \le C\left\| \nabla \left( \psi \frac{f_\varepsilon }{\varepsilon |\nabla u_\varepsilon |}\right) \right\| _{L^p(\Omega )}\left\| \nabla \left( \psi \frac{f_\varepsilon }{\varepsilon |\nabla u_\varepsilon |}\right) \right\| ^\frac{(p-1)(n+1)}{n+1-p}_{L^p(\Omega )}\\&\quad \le C_\psi \left\| \frac{f_\varepsilon }{\varepsilon |\nabla u_\varepsilon |}\right\| _{W^{1,p}(\Omega } \end{aligned}$$
  where we have $q_0=\frac{pn}{n+1-p}>n$ since $p>\frac{n+1}{2}$.
3. If $n+1=2$ then this is proven in [8]. For $n+1\ge 3$ it can be directly verified that the condition (3) implies the conditions in Theorem 1.1.

$$\square $$

Secondly, we prove the $\Gamma$ - convergence of the $L^{q_0}, q_0>n$ “Allen-Cahn" mean curvature functional to the $L^{q_0}$ mean curvature functional for hypersurfaces in ${\mathbb {R}}^{n+1}$.

### Proof of 1.2

The $\Gamma$ - convergence of the first term in the functional $\int _\Omega \left( \frac{\varepsilon |\nabla u|^2}{2}+\frac{W(u)}{\varepsilon }\right) dx$ to the perimeter functional $\alpha {\mathcal {H}}^n(\partial E\cap \Omega )$ was proved by Modica [5].

The limsup inequality for the $\Gamma$ - convergence of $\int _\Omega \left( \frac{|\varepsilon \Delta u -\frac{W'(u)}{\varepsilon }|}{\varepsilon |\nabla u|}^{q_0}\right) \varepsilon |\nabla u|^2dx$ follows from a similar argument as in [2], using a smooth approximation of the boundary measure and a diagonal argument (see also [7]).

The liminf inequality for the $\Gamma$ - convergence of $\int _\Omega \left( \frac{|\varepsilon \Delta u -\frac{W'(u)}{\varepsilon }|}{\varepsilon |\nabla u|}^{q_0}\right) \varepsilon |\nabla u|^2dx$ to the $L^{q_0}$ functional follows from (4.17) in the proof of Theorem 4.6. $\square$

## Data Availability

Data sharing not applicable to this article as no datasets were generated or analysed during the current study.
